# Acute Hypoxia Alters Extracellular Vesicle Signatures and the Brain Citrullinome of Naked Mole-Rats (*Heterocephalus glaber*)

**DOI:** 10.3390/ijms23094683

**Published:** 2022-04-23

**Authors:** Stefania D’Alessio, Hang Cheng, Liam Eaton, Igor Kraev, Matthew E. Pamenter, Sigrun Lange

**Affiliations:** 1 Tissue Architecture and Regeneration Research Group, School of Life Sciences, College of Liberal Arts and Sciences, University of Westminster, London W1W 6 UW, UK; w1650366@my.westminster.ac.uk; 2 Department of Biology, University of Ottawa, Ottawa, ON K1N 6N5, Canada; hchen188@uottawa.ca (H.C.); leato026@uottawa.ca (L.E.); mpamenter@uottawa.ca (M.E.P.); 3 Electron Microscopy Suite, Faculty of Science, Technology, Engineering and Mathematics, Open University, Walton Hall, Milton Keynes MK7 6AA, UK; igor.kraev@open.ac.uk; 4 Brain and Mind Research Institute, University of Ottawa, Ottawa, ON K1H 8M5, Canada

**Keywords:** *Heterocephalus glaber*, peptidylarginine deiminase (PAD), deimination/citrullination, extracellular vesicles (EVs), KEGG, central nervous system, plasma, hypoxia, in vivo

## Abstract

Peptidylarginine deiminases (PADs) and extracellular vesicles (EVs) may be indicative biomarkers of physiological and pathological status and adaptive responses, including to diseases and disorders of the central nervous system (CNS) and related to hypoxia. While these markers have been studied in hypoxia-intolerant mammals, in vivo investigations in hypoxia-tolerant species are lacking. Naked mole-rats (NMR) are among the most hypoxia-tolerant mammals and are thus a good model organism for understanding natural and beneficial adaptations to hypoxia. Thus, we aimed to reveal CNS related roles for PADs in hypoxia tolerance and identify whether circulating EV signatures may reveal a fingerprint for adaptive whole-body hypoxia responses in this species. We found that following in vivo acute hypoxia, NMR: (1) plasma-EVs were remodelled, (2) whole proteome EV cargo contained more protein hits (including citrullinated proteins) and a higher number of associated KEGG pathways relating to the total proteome of plasma-EVs Also, (3) brains had a trend for elevation in PAD1, PAD3 and PAD6 protein expression, while PAD2 and PAD4 were reduced, while (4) the brain citrullinome had a considerable increase in deiminated protein hits with hypoxia (1222 vs. 852 hits in normoxia). Our findings indicate that circulating EV signatures are modified and proteomic content is reduced in hypoxic conditions in naked mole-rats, including the circulating EV citrullinome, while the brain citrullinome is elevated and modulated in response to hypoxia. This was further reflected in elevation of some PADs in the brain tissue following acute hypoxia treatment. These findings indicate a possible selective role for PAD-isozymes in hypoxia response and tolerance.

## 1. Introduction

Naked mole-rats (*Heterocephalus glaber*) are a eusocial and subterranean mammalian species with unusual resistance to hypoxia, cancer, and ageing, among other remarkable abilities [1,2]. Indeed, naked mole-rats are one of the most hypoxia-tolerant adult mammals and can tolerate minutes of anoxia, hours at 3% O_2_, and days to weeks at 8% O_2_ [3,4,5,6]. As a result, naked mole-rats are emerging as a key model organism for the study of naturally evolved mechanisms of hypoxia tolerance, from whom insight may be gained into novel molecular pathways that are protective against hypoxia-related pathologies, including age-related diseases and neurological disorders [7,8].

Naked mole-rats respond to hypoxia with a suite of metabolic modifications to reduce energy demand and maximize the efficiency of metabolic pathways. For example, naked mole-rats exhibit a rapid and robust decrease in metabolic rate in acute hypoxia, by as much as 85% of resting metabolism [5,9], while still retaining consciousness and staying somewhat active [2]. This hypometabolic shift is supported in part by decreases in behaviour [10,11,12] and thermogenesis [13,14], and in energy-consuming cellular processes [15,16,17,18,19,20,21]. In addition, blood glucose increases in hypoxia, metabolic pathways are reorganized to favour carbohydrate metabolism [9,22], and mitochondrial respiration becomes more efficient (by increasing the coupling of ATP generation to O_2_ consumption, decreasing proton leaks, etc.) [5,23,24]. Indeed, the metabolic demands of at least cardiac and skeletal muscle and brown adipose tissue are all decreased in hypoxic naked mole-rats.

Intriguingly, the metabolic response of naked mole-rats to hypoxia may include the reorganization of energetic use to prioritize the demands of the brain, which is among the most hypoxia-sensitive organs in the body [25]. For example, AMP-activated protein kinase (AMPK) is regulated by miRNAs in a tissue-specific manner that likely downregulates glycolysis in skeletal muscle while upregulating glycolysis in the brain [18,19]. At the same time, brain Na^+^/K^+^-ATPase activity is selectively downregulated in some regions of the brain to conserve energy [26], and brain mitochondrial ATP generation becomes more tightly coupled to O_2_ consumption (i.e., becomes more efficient). As a result, ATP concentration is maintained in the naked mole-rat brain in hypoxia, and deleterious glutamate release and downstream excitotoxicity, which are hallmarks of the hypoxia-intolerant mammal brain [27,28], are avoided in the hypoxic naked mole-rat brain [9,29,30,31]. Furthermore, naked mole-rat brain mitochondria retain respiratory capacity and membrane integrity following ischaemia, indicating tolerance-related modification in respiratory pathway control compared to mouse brains [7]. Taken together, these data indicate that naked mole-rats employ a variety of cellular and systemic metabolic modifications in hypoxia to support their overall hypoxia tolerance and protect the brain when O_2_ is limited.

PADs are a family of five enzymes in mammals, which have also been identified in the naked mole-rat [32]. PADs cause post-translational citrullination/deimination, a calcium-catalysed post-translational modification that converts arginine in proteins to citrulline [33,34,35,36]. Deimination is associated with a range of pathological conditions, including cancer and inflammatory, autoimmune, and neurological diseases, among others [37,38,39,40,41]. Indeed, roles for PADs have been identified in various pathologies of the central nervous system (CNS), including in acute injury, chronic conditions, neurodegeneration, and brain cancer [42,43,44,45,46,47,48,49,50,51,52,53,54,55]. Furthermore, PADs are associated with a rise in deimination/citrulline-mediated responses in hypoxic conditions in the CNS [44,56,57,58,59,60,61], as well as in other instances of hypoxia such as haemorrhagic shock [62], myocardial infarction [63], necrotizing enterocolitis [64], autoimmunity [65], and cancer [66].

EVs are circulatory membrane vesicles in body fluids, including plasma, and play important roles in cell communication and pathological processes via transport of various EV cargo, including modified protein cargo. EVs can be indicative of various physiological and pathological responses [67], and EV signatures have been linked to a range of hypoxia-related diseases [67,68,69,70] and to various CNS pathologies in relation to protein deimination content in neurodegeneration [55] and in brain cancer [51,52]. Changes in circulating EV signatures have also been linked to a range of hypoxia-related diseases, including cardiovascular and metabolic disease, as well as in cancer [67]. Taken together, EV fingerprinting approaches may mirror health or disease status and may thus allow for a liquid biopsy approach to identifying systemic responses to various stimuli. Therefore, investigating EV signatures following acute hypoxia in hypoxia-tolerant species such as naked mole-rats may reveal roles for EVs in beneficial systemic and cellular responses to hypoxia.

Therefore, we used naked mole-rats as a model hypoxia-tolerant mammalian organism in which to study PAD-related responses in the brain and EV signatures in response to a hypoxia challenge. Recently, we assessed citrullination in plasma and plasma EVs in naked mole-rats at baseline conditions for the first time, linking this modification to a range of metabolic and immune-associated pathways. However, the roles of PADs in hypoxia, and of circulating EV signatures in response to hypoxia, have yet to be explored in this model. Important as well, as roles for PADs in regulating brain responses to hypoxic insult have previously been shown and also linked to neurodegeneration, we sought to investigate the brain citrullinome of naked mole-rats following a hypoxia challenge, as this may reveal a putatively beneficial phenotype for hypoxic injury responses in the CNS.

## 2. Results

### 2.1. Extracellular Vesicle Profiles in Naked Mole-Rat Plasma Change in Response to Hypoxia Treatment

**Figure 1:**
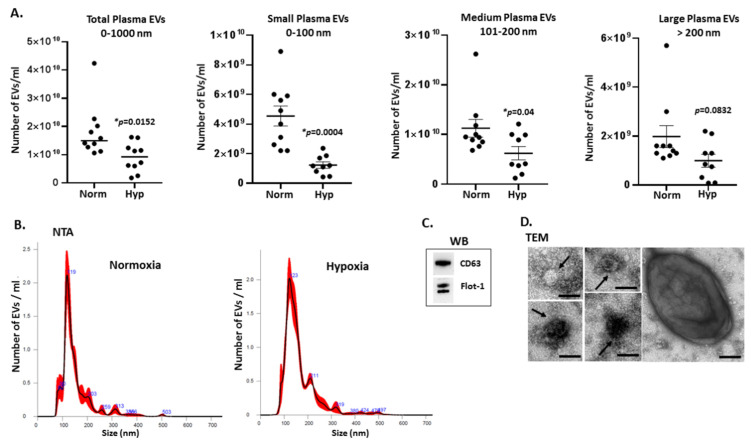
EV profile trends from plasma of naked mole-rats treated for 4 h in normoxia or hypoxia. (**A**) Number of EVs isolated from naked mole-rat plasma, comparing normoxia and hypoxia conditions. Changes were assessed in release profiles of total EVs (0–1000 nm), small EVs (<100 nm), medium-sized EVs (101–200 nm) and large EVs (201–1000 nm); based on measurement of plasma EVs from 10 animals per group; error bars represent standard error of mean (SEM); *t*-test, exact *p*-values are shown, *p* < 0.05 considered statistically significant (indicated by *). (**B**) Representative NTA curves of plasma EVs from naked mole-rats following normoxia or hypoxia treatment, respectively; (**C**) Western blotting analysis of EV markers for naked mole-rat plasma EVs, showing positive for CD63 and Flotillin-1; (**D**) Transmission electron microscopy (TEM) of plasma-EVs from naked mole-rats, showing representative images of the differently sized EVs; scale bar indicates 100 nm, black arrows highlight individual EVs.

Circulating plasma EVs from naked mole-rats treated for 4 h in normoxia or hypoxia were profiled by NTA for assessment of changes in total EV numbers, as well as for EV subgroups based on size. In addition to total EVs (0–1000 nm), EVs were also separately counted in the size ranges of 0–100 nm (“small EVs”); 101–200 nm (“medium-sized EVs”) and >200 nm (“large EVs”), based on NTA measurements (Figure 1). A statistically significant change was observed in total EV numbers between the two groups (0–1000 nm), with reduced EV levels in plasma following hypoxia. Changes in release of different EV subsets was also observed, with a significant decrease in small EVs (<100 nm) following hypoxia, significant reduction in medium sized (101–200 nm) EVs following hypoxia, while larger EVs (>200 nm) showed also a trend for reduced numbers following hypoxia, albeit not statistically significant (n=10 per group; exact *p*-values are indicated on the graphs; *p* <0.05 considered statistically significant; error bars indicate SEM).

### 2.2. Proteomic Profiles of Naked Mole-Rat Plasma EVs Change Following Hypoxia

**Figure 2:**
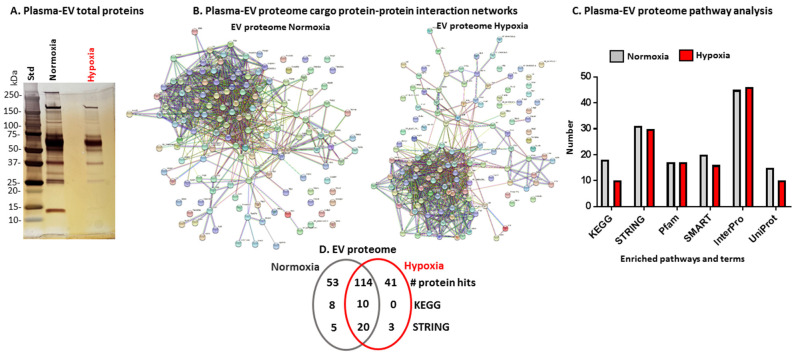
Total proteomic cargo of plasma EVs from normoxia- and hypoxia-treated naked mole-rats. (**A**) SilverGel showing total protein EV cargo that was then subjected to LC-MS/MS analysis. (**B**) Protein interaction networks for the plasma EV proteome of normoxia- and hypoxia-treated naked mole-rats. (**C**) Histogram showing the number of pathway analysis terms associated with the proteome of EVs from normoxia- and hypoxia-treated animals (*n* = 5 animals per group). (**D**) Venn diagram showing unique and shared protein hits, and KEGG and STRING pathways between EV proteomes of the normoxia and hypoxia groups (*n* = 5 animals per group).

Total proteomic content of plasma EVs from naked mole-rats treated for 4 h in normoxia or hypoxia (Figure 2A) were compared using LC-MS/MS analysis of total EV protein content (*n* = 5 per group). Protein hits identified in plasma EVs for both groups are listed in Table 1, which indicates shared and unique hits.

**Figure 3:**
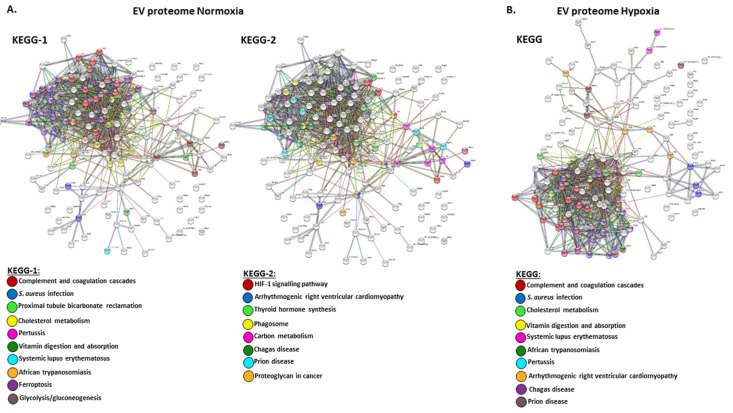
KEGG pathway analysis for protein network analysis for EV total protein cargo, showing predicted protein networks annotating associated KEGG pathways for total protein of plasma EVs from (**A**) normoxia-treated mole-rats and (**B**) hypoxia-treated mole-rats.

Protein lists for each group were then subjected to STRING analysis (Searching Tool of Retrieval of Interacting Genes/Proteins; https://string-db.org/, accessed on 10 March 2022) to generate protein-interaction networks (Figure 2B) and pathway analysis for the EV proteome (Figure 2C and Figure 3, Table 2). The PPI enrichment *p*-value for both networks was <1.0 × 10^−16^, indicating more interactions than expected for a random set of proteins of similar size drawn from the genome, indicating that the proteins are at least partially biologically connected as a group.

The protein cargo of circulating plasma EVs from naked mole-rats from normoxia and hypoxia treatment was then compared for predictive protein networks. Overlapping KEGG pathways for normoxia and hypoxia EV cargo proteomes were complement and coagulation cascades, *Staphylococcus aureus* infection, cholesterol metabolism, pertussis, vitamin digestion and absorption, systemic lupus erythematosus, African trypanosomiasis, arrhythmogenic right ventricular cardiomyopathy, Chagas disease, and prion disease. KEGG pathways specific to the normoxia EV proteome were proteoglycan in cancer, proximal tubule bicarbonate reclamation, ferroptosis, glycolysis/gluconeogenesis, HIF-1 signalling pathway, thyroid hormone synthesis, phagosome, and carbon metabolism (Figure 3 and Table 2A). No KEGG pathways were specific to the hypoxia EV proteome only.

The EV-cargo proteomes of normoxia- and hypoxia-treated animals had several overlapping local network clusters (STRING) (Table 2B), including complement and coagulation cascades, serpin, synapse pruning, fibrinolysis, fibrinogen, transferrin, hemopexin, cholesterol metabolism and efflux, regulation of lipoprotein l, triglyceride transport, glycophorin A, intermediate filament protein, and high-density lipoprotein particles. Local network clusters (STRING) specific for the normoxia EV proteome were related to carbon metabolism, apical plasma membrane urothelial plaque, pentose phosphate pathway, glycolytic process, apple domain, antithrombin-iii, kininogen, and mitochondrial glycoprotein. Three STRING pathways were specific for the hypoxia EV proteome and related to keratin type, blood coagulation, fibrin clot formation, alpha2-antiplasmin, intermediate filament protein, and keratinocyte activation (Table 2B).

Common and distinct KEGG and STRING pathways related to total proteomic content of naked mole-rat plasma EVs from normoxia and hypoxia groups, respectively, are further listed and summarised in Table 2A,B.

### 2.3. Citrullinated Protein Profiles of Plasma EVs under Normoxic Versus Hypoxic Conditions in Naked Mole-Rats

**Figure 4:**
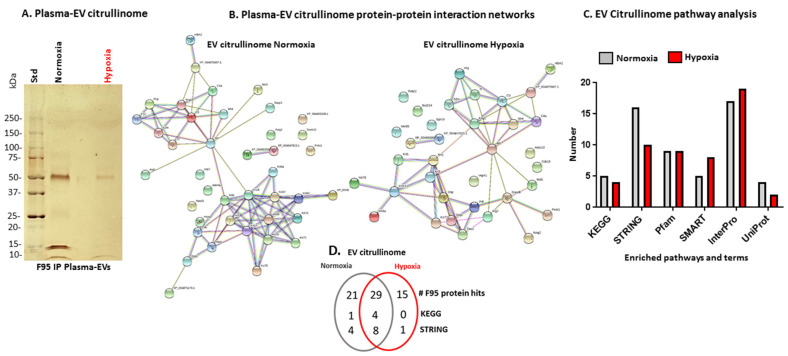
F95-enriched protein cargo of plasma EVs (EV citrullinome) from normoxia- and hypoxia-treated naked mole-rats. (**A**) SilverGel showing F95-enriched protein fractions from EVs (EV citrullinome) that were then subjected to LC-MS/MS analysis. (**B**) Protein–protein interaction networks created in STRING for the plasma-EV citrullinome of normoxia- and hypoxia-treated naked mole-rats. (**C**) Histogram showing number of pathway analysis terms associated with the EV citrullinome from normoxia- and hypoxia-treated animals. (**D**) Venn diagram summarising deimination/citrullination (F95) hits and main KEGG and STRING pathways related to these hits, indicating shared or distinct hits and pathways between the groups (*n* = 5 animals per group).

F95-enriched proteins identified in naked mole-rat plasma EVs were analysed by SDS-PAGE and silver staining (Figure 4A). To compare citrullinated proteins from EV cargo of plasma from animals under normoxic and hypoxic conditions, fractions were subjected to LC-MS/MS analysis. A total of 29 protein hits were common between both groups, whereas 21 hits were identified as deiminated only in normoxia and 15 hits were found deiminated in the hypoxia plasma EVs only (*n* = 5 per group). The protein hits are listed in Table 3, highlighting common and specific hits, and protein interaction networks were created in STRING (Figure 4B). Pathway analysis in STRING revealed a number of differences between the normoxia and hypoxia groups (Figure 4C and Table 4), as summarised in the Venn diagram in Figure 4D. The PPI enrichment *p*-value for both networks was <1.0 × 10^−16^, indicating more interactions than expected for a random set of proteins of similar size, drawn from the genome, indicating that the proteins are at least partially biologically connected as a group.

**Figure 5:**
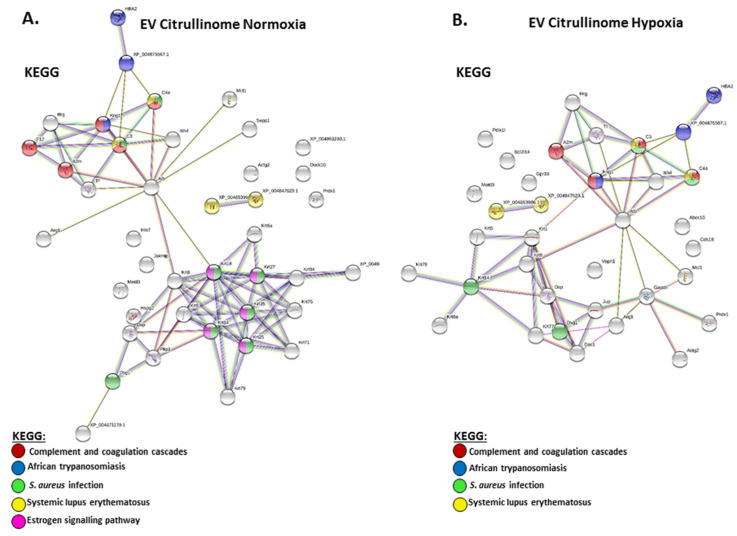
STRING analysis for the EV citrullinome, showing predicted protein networks and associated KEGG pathways for the plasma EV citrullinome from (**A**) normoxia-treated naked mole-rats and (**B**) hypoxia-treated naked mole-rats.

Furthermore, KEGG analysis of the EV citrullinome highlighted four shared pathways between the groups and one specific pathway (oestrogen signalling) for the normoxia group, as shown in Figure 5.

### 2.4. PAD Isozyme Levels Differ in Naked Mole-Rat Brains from Normoxia and Hypoxia Groups

**Figure 6:**
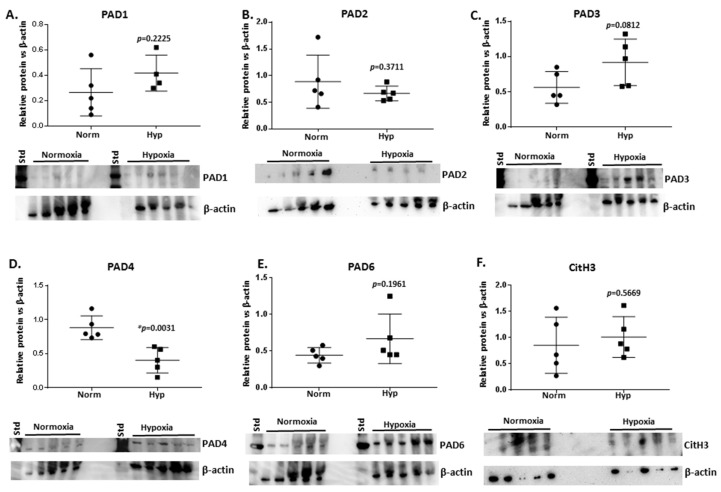
PAD isozyme and CitH3 protein levels in brains of naked mole-rats following normoxia and hypoxia treatment, showing (**A**) PAD1, (**B**) PAD2, (**C**) PAD3, (**D**) PAD4, (**E**) PAD6, and (**F**) CitH3. Protein levels were assessed in *n* = 5 brains per group and normalised against beta-actin protein levels; exact *p*-values are indicated (*t*-test; * indicates significance at *p* < 0.05; circles represent normoxia and squares hypoxia brain samples, respectively) and the error bar represents SD.

Using Western blotting analysis of brain protein lysates from normoxia- and hypoxia-treated naked mole-rats (*n* = 5 per group), protein levels of the five PAD isozymes (PAD1,2,3,4 and 6) were assessed (Figure 6A–E). Blots were carried out based on cross-reactivity with human PAD isozyme-specific antibodies (see Supplementary Figure S1 for phylogenetic analysis of naked mole-rat PAD isozymes in comparison with human PADs, confirming conservation of the corresponding PAD forms between humans and naked mole-rats). Overall, PAD2 showed no significant changes, with a possible trend for reduced levels (albeit high variability was observed between samples, not reaching statistical significance, and one outlier was observed), and PAD4 protein levels were statistically significantly reduced in the hypoxia-challenged brains (*p* = 0.0031), whereas PAD1, PAD3, and PAD6 levels showed an overall trend for elevation in the hypoxia-challenged brains (albeit not reaching statistical significance and with considerable individual variability observed). Histone H3 citrullination (CitH3) was also assessed by Western blotting as a representative marker for ETosis and epigenetic changes via this post-translational modification (using the anti-histone H3 citrulline R2 + R8 + R17 antibody), indicating no significant changes in the hypoxia brains compared to the controls when normalised against the beta-actin loading control (Figure 6F). Furthermore, the citH3 antibody showed a number of unspecific bands at higher molecular weight, whereas the approximately 17 kDa band shown in Figure 6F was used for densitometry analysis and was at the expected size reported for citH3.

### 2.5. The Brain Citrullinome of Naked Mole-Rats Is Increased Following Hypoxic Challenge

**Figure 7:**
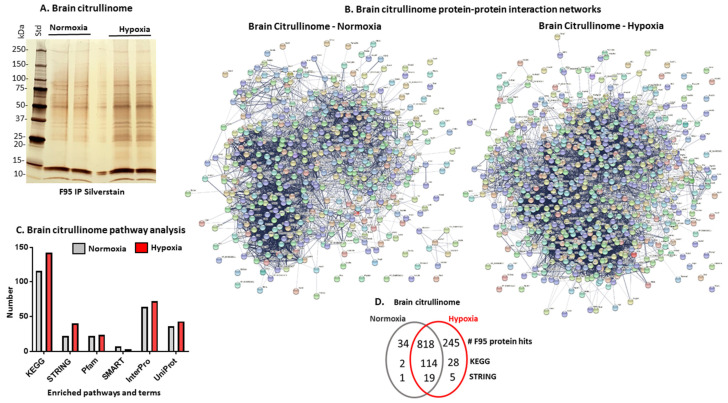
The brain citrullinome of naked mole-rats following normoxia or hypoxia treatment. (**A**) SilverGel showing F95-enriched proteins from control brains (normoxia) and brains taken from animals after a hypoxia challenge; *n* = 5 (pool of 5 brains per group; 2 experimental replicates). (**B**) Protein-interaction networks for all deiminated protein candidates identified in naked mole-rat brains following normoxia or hypoxia (brain citrullinome). (**C**) STRING pathway analysis results for KEGG and GO terms for the full brain citrullinome following normoxia or hypoxia treatment. (**D**) Venn diagram summarising deimination/citrullination hits (F95) and shared and specific pathways for the citrullinome between normoxic and hypoxic brains (*n* = 5 brains per group in all experiments).

F95-enriched proteins isolated from brains of naked mole-rats that had been exposed to hypoxia, relative to the normoxia control group, were analysed by SDS-PAGE and silver staining (Figure 7A) and by LC-MS/MS for identification of citrullinome brain signatures (Figure 7B; Supplementary Tables S1 and S2 for full protein lists per group). A total of 852 protein hits were identified in the normoxic brains, whereas 1222 hits were identified in the hypoxic brains. Therein, 34 deimination hits were specific to the normoxic brains and 245 hits were specific to the hypoxic brains (*n* = 5 brains per group). STRING analysis was used for identification of protein–protein interaction networks and associated pathways (Figure 7B,C). Local network clusters (STRING) and KEGG pathways identified for all F95-enriched hits in the normoxic brains and hypoxic brains are listed in Table 5 and Table 6, respectively; a summary of overlapping and specific protein hits and pathways is presented in Figure 7D. The PPI enrichment *p*-value for both networks was <1.0 × 10^−16^, indicating more interactions than expected for a random set of proteins of similar size drawn from the genome, indicating that the proteins are at least partially biologically connected as a group.

**Figure 8:**
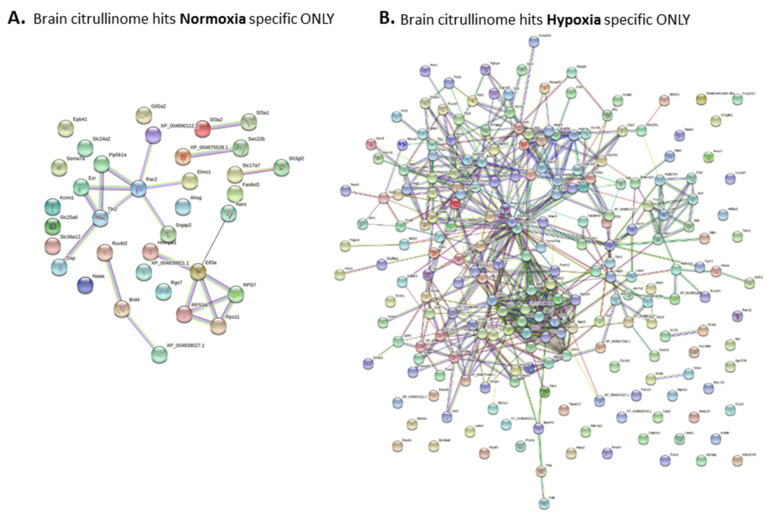
Protein networks built based on F95-enriched proteins identified in either the normoxia or hypoxia brains only. (**A**) F95 hits from normoxia brains only (not overlapping with the hypoxia group); (**B**) F95-enriched proteins identified in hypoxia brains only (not overlapping with the normoxia group).

In addition, a protein interaction network analysis was specifically performed for F95 hits identified only in the normoxia or hypoxia group. Protein networks created for these protein hits identified as deiminated/citrullinated only in the normoxia or hypoxia group therefore excluded all common identified deiminated/citrullinated proteins between the two groups (Figure 8). Using this analysis approach, no significant functional enrichments were identified for KEGG pathways in the normoxia F95-positive-hits-only network (Figure 8A; PPI enrichment *p*-value 0.0326), whereas 50 KEGG pathways were identified for the hypoxia-specific-only F95 positive hits (Figure 8B; PPI enrichment *p*-value <1.0 × 10^−16^); these are listed in Table 7, also indicating the observed gene count for each pathway.

## 3. Discussion

The current study assessed circulating EV signatures (whole proteome and citrullinated proteins) and changes relating to protein citrullination in the brains of naked mole-rats following hypoxia treatment, compared with normoxia-treated control animals. We identified large-scale changes in EV proteome signatures and the brain citrullinome between normoxia- and hypoxia-treated animals.

### 3.1. EV Proteomic Signatures in Normoxia- and Hypoxia-Treated Naked Mole-Rats

Some shift was observed in plasma-EV profiles released between normoxia and hypoxia treated animals, with an overall decrease of plasma-EVs following hypoxia, which was significant for smaller and medium sized EVs (<100 nm; 101–200 nm), and a trend for reduction in larger EVs (201–1000 nm) was also observed. Increased EV release overall is linked to hypoxia in other models, including human [69,70], while it has been suggested that an increase in larger EVs is linked to inflammatory responses in human disease [71]. Notably, we observed a trend for decrease in numbers of EVs, including larger EVs above 201 nm. The changes in naked mole-rat plasma-EVs with hypoxia may indicate changes in cargo-export and modulated inflammatory responses to hypoxia and/or protective anti-inflammatory systemic responses in this species, which shows unusual hypoxic tolerance. This possibility warrants further investigation. Indeed, an interplay between hypoxia and EVs has been reported for many inflammatory conditions, and hypoxia creates an inflammatory environment in other species [72,73]. Hypoxia-induced EVs in other species may also contribute to disease pathogenesis via cargo transfer (reviewed in [69]). However, it must be noted that cargo transfer of proteins, DNA, RNA and non-coding RNA species may vary between the different types of EVs released, and our current study assesses protein content only. Interestingly, we observe a higher protein content (including citrullinated proteins) and a higher number of KEGG pathways relating to the total proteome of EVs from normoxia, versus hypoxia treated animals. Therefore, our findings indicate that in this hypoxia tolerant animal, circulating EV proteomic content is reduced in hypoxia. This could be indicative of systemic responses and redirection of resources in response to hypoxic insult. 

To understand differences in pathobiological and physiological pathways associated with the EV proteomes of normoxia versus hypoxia, protein–protein interaction network analysis was carried out. Several KEGG pathways were identified as specific to the total EV proteome of normoxic animals, including phagosome and carbon metabolism, ferroptosis, proteoglycan in cancer, proximal tubule bicarbonate reclamation, glycolysis/gluconeogenesis, thyroid hormone synthesis, and HIF-1 signalling pathway, whereas no KEGG pathways were specific to the hypoxia EV proteome only. Overlapping KEGG pathways for normoxia and hypoxia EV proteome were complement and coagulation cascades, *Staphylococcus aureus* infection, pertussis, systemic lupus erythematosus, African trypanosomiasis, Chagas disease, prion disease, arrhythmogenic right ventricular cardiomyopathy, cholesterol metabolism, and vitamin digestion and absorption. This indicates that a number of critical immune-related pathways are influenced by EV-mediated proteome transport, and that there is furthermore a higher number of metabolic pathways influenced by EV-transported protein cargo during normoxia compared to hypoxia.

In addition, local network cluster analysis (STRING) indicated that the EV proteomes in normoxia and hypoxia had a number of overlapping STRING pathways, including complement and coagulation cascades, serpin, synapse pruning, fibrinolysis, fibrinogen, transferrin, hemopexin, cholesterol metabolism and efflux, regulation of lipoprotein l, triglyceride transport, glycophorin A, intermediate filament protein, and high-density lipoprotein particles. STRING pathways specific to the normoxia EV proteome were related to carbon metabolism, apical plasma membrane urothelial plaque, pentose phosphate pathway, glycolytic process, apple domain, antithrombin-iii, kininogen, and mitochondrial glycoprotein. Three STRING pathways were specific to the hypoxia EV proteome and related to keratin type, blood coagulation, fibrin clot formation, alpha2-antiplasmin, intermediate filament protein, and keratinocyte activation. As with the KEGG pathway analysis, this analysis indicates that critical pathways for energy and metabolism are influenced by EV protein transport in normoxia, and furthermore that EV protein cargo participates in key immune pathways, as well as metabolic pathways, in normoxia and in response to hypoxia. Furthermore, pathways linked to blood coagulation and fibrin clot formation identified here in the hypoxia EV cargo correlate with previous studies identifying coagulopathies in hypoxic events and infection [74,75].

### 3.2. EV Citrullinome Signatures in Normoxia- and Hypoxia-Treated Naked Mole-Rats

The plasma EV citrullinome was also assessed based on the enrichment of citrullinated/deiminated proteins isolated from the EVs. KEGG pathways for citrullinated EV protein cargo were found to be fewer in the hypoxia compared with the normoxia group, with four common pathways, all linked to immunity (complement and coagulation cascades, African trypanosomiasis, *S. aureus* infection, and systemic lupus erythematosus); no further pathways were identified for the hypoxia EV citrullinome, and the oestrogen signalling pathway was specific to the normoxia EV citrullinome only. Therefore, some main pathways in immune responses are influenced by deimination in circulatory EV cargo transport both in hypoxia and normoxia conditions. For example, oestrogen signalling has multifaceted functions in health and disease, and beyond endocrinal roles also regulates epigenetic mechanisms [76,77].

STRING pathways specific for the citrullinome of hypoxia EVs were desmosome and cell–cell adhesion mediated by cadherin. This related to the differences observed in target proteins of deimination in the normoxia versus hypoxia EV citrullinome. In hypoxia, several proteins with roles in cytoskeletal function were identified, including tubulin and plectin, which is a key cytoskeleton interlinking molecule with multifaceted roles in mediating intermediate filament network functions in physiological and pathobiological processes [78,79]. EV biogenesis may furthermore be affected by deimination of cytoskeletal proteins [80]. Glyceraldehyde-3-phosphate dehydrogenase, identified in the hypoxia EV citrullinome, is crucial for the glycolysis pathway and is involved in apoptosis and hypoxic responses [81,82]. It has previously been reported as deiminated, including in brain cancer [51], and such a modification may contribute to its multifaceted functions, including the hypoxic response. Apoptosis facilitator Bcl-2-like protein 14 was also identified in the hypoxia EV citrullinome, highlighting roles for deimination in regulating apoptotic processes. Serine/threonine-protein kinases PDIK1L and PCTAIRE-3 were identified in the hypoxia EV-citrullinome. PCTAIRE-3 is mainly expressed in differentiated neurones and has been linked to Alzheimer’s disease pathology, both relating to amyloid precursor protein-dependent Alzheimer’s pathology via phosphorylation [83] and by regulation of tau phosphorylation [84]. Multidrug resistance-associated protein 7 was identified in the hypoxia EV citrullinome; although deimination needs to be studied in this context, multidrug resistance is linked to hypoxia [85]. G-protein-coupled receptor 19 was identified in the EV citrullinome in hypoxia, and although effects of its deimination have not been reported, it has been linked to neonatal HI-induced brain damage [86]. Fatty acid-binding protein, identified in the EV-citrullinome in hypoxia, is linked to infection and injury in hypoxic challenge [87,88]. Ventricular zone-expressed PH domain-containing protein was identified in the hypoxia EV citrullinome. Although originally identified as having roles in neural cell differentiation, it is a multifaceted adaptor protein with roles in signal transduction in numerous physiological and pathological processes, including via TGF-beta, BMP, and SMAD signalling [89]. VEPH1 has been reported in EV cargo of various cell types, including in hypoxia and normoxia [90], but hitherto not in deiminated form.

### 3.3. Changes in PADs and the Brain Citrullinome

In the hypoxic brains, a trend for elevation in PAD isozymes PAD1, PAD3, and PAD6 was observed (albeit not reaching statistical significance), whereas PAD2 was not significantly changed (but showed some trend for reduction considering outliers) and PAD4 protein levels were significantly reduced. This modulation in PAD protein levels may link to some of the changes observed in the elevated and modified brain citrullinome in response to hypoxia. It must be considered that the PAD isozymes differ somewhat in preference of target proteins, and therefore a change in expression of the different isoforms may contribute to changes to the brain citrullinome. Upregulation of PAD1 and PAD3 has been reported in response to hypoxia in malignant glioma cells [58]. PAD1 has been strongly associated with skin physiology and skin diseases [91] and embryo development [36,92], and novel roles in breast cancer metastasis and epithelial-mesenchymal transition have been identified [93]. However, PAD1 has not received attention in relation to the CNS. PAD3 has previously been linked to CNS regeneration and remodelling, including in hypoxic injury, as well as stem-ness, neural stem cell death, and survival [43,44,51,94,95]. PAD3 has also been linked to different invasion abilities in brain cancer (glioblastoma multiforme) [52]. Interestingly, both PAD1 and PAD3 have recently been reported as upregulated by hypoxia and regulating glycolysis and cancer cell proliferation by citrullinating pyruvate kinase [96]. Glycolysis and pyruvate pathways were deimination-associated in both the normoxia and hypoxia brains. The role for PAD6 is mainly linked to developmental processes, including oocyte formation and embryo pre-implantation [97,98,99], whereas roles for PAD6 in the CNS have not been investigated. PAD2 is considered the most evolutionary conserved PAD isoform, and its elevation has been linked to neurodegenerative diseases [39,40,46,47,48,49,53,54,55]. Therefore, the observation of little change in this isozyme and possible trend for its somewhat reduced expression in the naked mole-rat brain following hypoxia is of interest. PAD2 is upregulated in vitro in astrocytes in response to hypoxia [56] and in malignant glioma cells in response to hypoxia [58]. The lack of any considerable changes in PAD2 may therefore indicate a protective mechanism in response to hypoxic insult in the naked mole-rat. Besides roles in epigenetic regulation via histone citrullination, a role for PAD4 in ETosis (NETosis and METosis) is part of a double-edged sword in responses to pathological stimuli, including in hypoxia, and may cause damage of self. PAD4 has been shown to be activated in response to hypoxia in cancer [66], linked to hypoxia in autoimmune disease [100], and upregulated in malignant glioma cells in hypoxia challenge [58] as well as in neurodegeneration [55]. Therefore, our current finding that PAD4 was significantly downregulated in the naked mole-rat brain following hypoxia indicates that this may be a protective mechanism and contribute to its unusual hypoxic tolerance.

Changes in PAD regulation may possibly be reflected in the observed changes of the brain citrullinome. The brain citrullinome was isolated from whole-brain-cell lysates using F95 enrichment in conjunction with LC-MS/MS analysis, and we report considerable increases in the number of deiminated protein hits in the hypoxia brains compared to normoxia brains. Whereas 107 KEGG pathways based on these hits were shared between the brain citrullinome of the hypoxia and normoxia brains, 28 KEGG pathways were specific for the total citrullinome of the hypoxia brains and 2 KEGG pathways to the whole citrullinome of the normoxia brains. Furthermore, a total of 50 KEGG pathways were related to protein networks created based on citrulline-specific proteins identified only in the hypoxia brains, whereas no KEGG pathways were found in similar networks created based on citrullinated proteins identified only in the control normoxia brains (this excluded any overlapping targets between the groups from the protein network analysis).

KEGG pathways relating to the hypoxia brain citrullinome related to a range of physiological and pathobiological mechanisms. This included the mRNA surveillance pathway, but also pathways related to hormonal control and metabolism, including oestrogen signalling pathway; GnRH secretion and GnRH signalling pathway; parathyroid hormone synthesis, secretion, and action; growth hormone synthesis, secretion, and action; mineral absorption; renin secretion; bile secretion; amino sugar and nucleotide sugar metabolism; fatty acid degradation and fatty acid metabolism; insulin signalling pathway; alcoholism; Cushing’s syndrome; purine metabolism; and histidine metabolism.

KEGG pathways for the hypoxia brain citrullinome relating to hemostasis and immunological/inflammatory pathways included platelet activation, apelin signalling pathway, human T-cell leukemia virus 1 infection, hepatitis B, chemokine signalling pathway, apoptosis, inflammatory mediator regulation of TRP channels, cellular senescence, renal cell carcinoma, and MAPK signalling pathway. Deimination of the apelin signalling pathway in hypoxia may be of considerable interest; deimination has also been identified in pre-motor Parkinson’s disease [55]. Although apelin signalling has multifaceted physiological functions, including in the CNS, it is associated with ischaemia as well as neovascularisation events such as retinopathies, tumours, and retinopathies [101], and a range of neurodegenerative diseases [102,103].

Analysis of local network clusters (STRING) identified 19 network clusters shared between the brain citrullinome of the hypoxia and normoxia brains, whereas five STRING network clusters were specific to the total citrullinome of the hypoxia brains (proteasome ribosome and ribosomal protein L23; ubiquinone and zinc-finger domain; mixed, incl. regulation of actin cytoskeleton and actin; septin) and one (GroEL-like equatorial domain superfamily) was specific to the whole citrullinome of the normoxic brains. The deimination of septin-related pathways may be of interest in hypoxic brains, as these play roles in many key cellular processes, regulate protein stability, and also play roles in protecting HIF-1a from degradation [104].

### 3.4. EV Signatures and the Brain Citrullinome in Relation to HIF-1 Regulation

In relation to hypoxia, it is interesting to observe that the HIF-1-signalling pathway was identified in whole EV proteome of normoxia- but not hypoxia-challenged animals. Furthermore, the HIF-1 KEGG pathway came up in the F95-enriched networks of both normoxic and hypoxic brains, although in the hypoxic brains there were 19 underlying genes associated, but only 17 for the normoxia brains. HIF-1 is a master regulator of oxygen homeostasis, contributes to hypoxia adaption of the naked mole-rat [105], and is highly expressed endogenously in naked mole-rats due to mutation in the VHL (Von Hippel–Lindau disease tumor suppressor) domain [106]. Naked mole-rats have a very high expression of HIF mRNA and protein in normoxia relative to mice [105], and interestingly, respond to acute hypoxia of 4 h by decreasing HIF expression in the brain [105]. In other models, HIF-1 signalling has been linked to regulation of EV release during hypoxia [107], including microvesicles (medium/larger EVs) [108] and small EV (exosome) release [109]. Although roles for deimination in regulating the HIF-1 pathway remain to be fully understood, including in the naked mole-rat, the KEGG HIF-1 pathway has previously been linked to deimination in other animal models of hypoxia resistance, including cetaceans [110]. In human cancer cell models, PAD4 is induced by hypoxia in a HIF-dependent manner, performing histone citrullination required for HIF-dependent transcriptional responses to hypoxia as well as for tumour vascularisation [66]. In addition, in malignant glioma cells, PAD1, 2, 3, and 4 were shown to be upregulated in a hypoxia-inducible factor-1-dependent manner at the mRNA level, albeit no verification of protein citrullination is provided in that study [5]. Collectively, a role for PADs in the hypoxic response via HIF-1 is of considerable interest, and findings from the current study point to differences in this interplay in the naked mole-rat, possibly reflecting some of the unusual hypoxic-resistance capacities of these animals.

### 3.5. Histones in EV Signatures and the Brain Citrullinome

Interestingly, in the whole EV proteome, histones H2A, H2B, and H3 were identified as EV cargo in the hypoxia group, whereas only histone H4 was identified as EV cargo in the normoxia group. When assessing deiminated/citrullinated EV cargo, deiminated histone H2A, H2B, and H3 were found in EVs of both groups, and H3.3 as deiminated in the hypoxia EVs only. In the brains, deiminated histones identified by proteomics in both groups included H2A, whereas in the hypoxia group H1.1 and H2B were also identified as deimination candidates, and H3 was identified in the normoxia group only. Using Western blotting, histone H3 deimination was further assessed in the brains of both normoxic and hypoxic brains as an indicator of ETosis, with no significant changes observed. This indicates that H3 deimination may not increase in hypoxia in naked mole-rat brains, contrary to what has been observed in other injury models of CNS and hypoxia, including in a mouse model of hypoxic ischaemic encephalopathy [44], acute ischaemic stroke [60] and spinal cord injury [43], as well as pre-motor Parkinson’s disease [55]. This also correlates with PAD4, which is considered the main driver of NETs in inflammation [63,111], being found to be significantly reduced in the hypoxic naked mole-rat brains in this current study, but having been, for example, linked with NETosis in neurological deficits following traumatic brain injury in other models [61]. PAD4 is also linked to hypoxia-induced autophagy [65]. Furthermore, PAD2, which is also linked to histone deimination [112], was observed here to not be affected by hypoxia, with a possible trend for some reduced levels (albeit not statistically significant) in hypoxic naked mole-rat brains. Conversely, PAD2 is upregulated in vitro in astrocytes of other species in response to hypoxia [56]. Roles for deimination of other histones including H1.1 and H2B may warrant further exploration in this context. In addition, whether circulatory histones in EVs observed here play systemic roles in hypoxia responses needs further assessment. The naked mole-rat has a particularly stable epigenome, which may contribute to its unusual physiological responses both to hypoxia as well as cancer resistance and longevity [113].

In summary, our findings indicate that circulating EV signatures are modified and proteomic content is reduced in response to acute hypoxic conditions in the naked mole-rat, including the circulating EV citrullinome, whereas the brain citrullinome is elevated. This is further reflected in changes of PAD isozyme expression in the hypoxic brains, which showed a trend for elevation of some PADs (PAD1, PAD3, and PAD6) and a trend for reduction in other PADs (PAD2 and PAD4) under hypoxic conditions, further indicating a possible selective role for PAD-isozymes and associated isozyme-specific deimination in hypoxia response and tolerance. The current study used acute hypoxia challenge, but longer periods of hypoxia exposure may also be of interest in the naked mole-rat model in future studies. It has to be noted that the current study focussed on EV proteome signatures only (total proteome and the citrullinome) based on the presence/absence of identified proteins in the respective experimental groups, but that EVs carry also a range of other cargo, including lipids, DNA, mRNA, miRNA, and non-coding RNA species. Such EV signatures may further contribute to hypoxia resistance and will remain subject to further investigation.

## 4. Materials and Methods

### 4.1. Animals

Naked mole-rats were group-housed in interconnected multi-cage systems at 30 °C and 21% O_2_ in 50% humidity and with a 12L:12D light cycle. Animals were fed fresh tubers, vegetables, fruit, and Pronutro cereal supplement ad libitum. Animals were not fasted prior to experimental trials. All experimental procedures were approved by the University of Ottawa Animal Care Committee (protocol #3444) in accordance with the Animals for Research Act and by the Canadian Council on Animal Care.

Animals (1–2-year-old subordinate males and females weighing 40–60 g) were exposed to either 21% O_2_ (normoxia) or 7% O_2_ (hypoxia) for 4 h. Each experimental group was comprised of 10 animals. Following treatment, the animals were sacrificed by cervical dislocation followed by rapid decapitation. Blood was collected in heparinised syringes and plasma was extracted by spinning whole blood at 1500 rpm for 15 min. Plasma aliquots were then frozen at −80 °C until analysis. Whole brains were rapidly extracted on ice and similarly frozen in liquid nitrogen, and then stored at −80 °C until analysis.

### 4.2. Extracellular Vesicle Isolation and Characterisation by NTA Analysis, Western Blotting, and Transmission Electron Microscopy

EVs were isolated from individual mole-rat plasma using differential centrifugation as previously described [32]. Per animal, 100 µL of plasma was added to 400 µL DPBS and then centrifuged at 4000× *g* for 30 min. The supernatant was collected and spun at 100,000× *g* for 1 h at 4 °C for collection of total EVs. The EV-enriched pellet was resuspended in 500 µL DPBS and centrifuged again at 100,000× *g* for 1 h at 4 °C. The supernatant was then discarded and the EV pellet was diluted in 100 µL DPBS.

For quantification of EVs by nanoparticle tracking analysis (NTA) in the individual plasma samples, 10 µL of diluted EV pellet was added to 990 µL DPBS and applied to the NS300 Nanosight (Malvern Panalytical Ltd., Malvern, UK) at syringe pump speed 50. Particles were recorded four times for 1 min per sample at camera level 9, and post-analysis was carried out at threshold level 5 with 40–60 particles per window. The four readings were then averaged per sample using the NTA software (version 3, Malvern, UK).

EVs were further analysed by Western blotting for two EV surface markers, CD63 (Abcam, 1/1000) and flotillin-1 (Abcam 1/1000; see below for further Western blotting details), and also visualised using transmission electron microscopy (TEM) according to methods previously described in [32]. Together, these approaches meet the minimum requirements for EV characterisation as per the guidelines of the International Society for Extracellular Vesicle Research [114].

### 4.3. Protein Isolation from Brain Tissue and Western Blotting

Proteins were extracted from brain tissue from treated animals (*n* = 5 per treatment group). Whole brains were homogenised in RIPA+ buffer (Sigma-Aldrich, Gillingham, UK, containing 10% protease inhibitor cocktail, Sigma-Aldrich) in 2 mL Eppendorf tubes on ice using a Mini Handheld Homogeniser (Kimble, DWK Life Sciences, VWR International). The homogenate was then gently pressed through a 23G needle into fresh Eppendorf tubes on ice, followed by gently pipetting up and down to eliminate any tissue clots. For each brain (400 mg tissue), 2 mL of RIPA+ buffer were used. The homogenates were then incubated on a roller for 1.5 h at 4 °C, pipetted up and down at regular intervals, and thereafter spun down at 16,000× *g* for 30 min at 4 °C for collection of isolated proteins. The extracted proteins were aliquoted and immediately frozen at −80 °C.

For SDS-PAGE and Western blotting, a 100 µL aliquot per sample was diluted with 100 µL 2× reducing Laemmli sample buffer (BioRad; containing 5% β-mercaptoethanol, Sigma-Aldrich) and boiled for 5 min at 100 °C. A 5 µL aliquot per sample was then applied to 4–20% TGX gels (BioRad, Watford, UK). SDS-PAGE was carried out at 165 V for 52 min. Gels were then transferred for Western blotting analysis using semi-dry transfer (1 h at 15V), and even protein transfer was assessed by PonceauS red stain (Sigma-Aldrich). The membranes were blocked in 5% bovine serum albumin (BSA, Sigma-Aldrich) in TBS-T for 1 h at room temperature (RT) and incubated in primary antibodies overnight at 4 °C on a shaking platform.

The primary antibodies used were anti-human PAD1 (ab181762, Abcam Cambridge, UK,), PAD2 (ab50257), PAD3 (ab50246), PAD4 (ab50247), PAD6 (PA5–72059, Thermo Fisher Scientific, Hemel Hempstead, UK), and pan-citrulline F95 (MABN328, Merck, Feltham UK [115], as well as citrullinated histone H3 (citH3, ab5103) antibodies, all diluted 1/1000 in TBS-T. Washing was carried out with TBS-T (3 × 10 min). Secondary antibody incubation was completed for 1 h at RT (using HRP-labelled anti-rabbit IgG or anti-mouse IgM antibodies; BioRad, diluted 1/3000 in TBS-T). Following washing (5 × 10 min in TBS-T), visualisation was carried out using ECL (Amersham Biosciences, Buckinghamshire, UK) and the UVP BioDoc-ITTM System (Thermo Fisher Scientific, Dartford, UK). Blots were re-probed for beta-actin (abcam, 1/5000 in TBS-T). Protein densitometry analysis was conducted using ImageJ [116].

### 4.4. F95-Enrichment for Deiminated Proteins from Plasma EVs and Brain Tissue

To identify deiminated/citrullinated proteins in plasma EVs and in brain tissue, deiminated proteins were enriched using the F95 pan-citrulline antibody (MABN328, Merck) in conjunction with the Catch-and-Release Immunoprecipitation Kit (Merck). For brain samples, protein extracts from 5 brains were pooled per experimental group. For EVs, EV isolates from 5 animals per group were pooled. Immunoprecipitation was carried out on agarose columns together with the F95 antibody and the affinity ligand overnight at 4 °C on a rotating platform. Proteins were eluted according to the manufacturer’s instructions (Merck, Watford, UK) and subjected either to SDS-PAGE and silver staining, or to LC-MS/MS analysis for identification of protein hits.

### 4.5. Silver Staining of Proteins and F95-Enriched Proteins

Total proteins of EVs and F95-enriched protein eluates from plasma EVs and brains were diluted 1:1 in 2× reducing Laemmli sample buffer, boiled for 5 min at 100 °C, and separated on 4–20% TGX gels (BioRad) for 52 min at 165 V. Following electrophoresis, the gels were silver stained using the BioRad Silver Stain Plus Kit according to the manufacturer’s instructions.

### 4.6. LC-MS/MS Proteomic Analysis

In-gel digestion was used for LC-MS/MS analysis, carried out by the Cambridge Centre for Proteomics (University of Cambridge, Cambridge, UK). Normoxic and hypoxic samples were subjected to LC-MS/MS analysis to determine (a) whole protein content of the plasma EVs, (b) F95-enriched protein eluates from the plasma EVs, and (c) F95-enriched protein eluates from the brains. The samples were prepared 1:1 in reducing Laemmli sample buffer, boiled and run 0.5 cm into a 10% TGX gel (BioRad), and then cut out as one whole band per sample (whole EV protein, F95-enriched EV proteins, F95-enriched brain proteins for normoxia versus hypoxia groups). Proteomic analysis was carried out by the Cambridge Centre for Proteomics (Cambridge, UK) according to previously described methods [32], and hits were assessed against the naked mole-rat protein database CCP_*Heterocephalus_glaber*_20190911 (21,449 sequences; 10,466,552 residues). In addition, a common contaminant database was also searched (cRAP 20190401; 125 sequences; 41,129 residues). Protein scores are derived from ion scores as a non-probabilistic basis for ranking protein hits; individual ion scores > 30 indicated identity or extensive homology (*p* < 0.05).

### 4.7. Protein Interaction Network Analysis

To identify local network clusters (STRING), gene ontology (GO) and Kyoto Encyclopedia of Genes and Genomes (KEGG) pathways for proteins from EVs (both total protein content and deiminated protein content), as well as deiminated hits from brain tissue, STRING analysis was used (https://string-db.org/, accessed on 10 March 2022). Predicted protein interaction networks were built based on hits identified from the LC-MS/MS analysis using the protein IDs and organism choice *Heterocephalus glaber* in the STRING software. For protein lists, “multiple proteins” was selected, confidence set at “medium,” and network interaction connecting lines were based on known and predicted interactions. Protein networks were annotated for pathway analysis and data were exported as labelled network images for KEGG and/or Excel files for KEGG pathways and STRING network clusters.

### 4.8. Statistical Analysis

To compare datasets from hypoxia- versus normoxia-treated groups, GraphPad Prism version 7 was used. T-tests were used to determine significance between groups for densitometry readings from Western blotting analysis (*n* = 5 per experimental group) and for the EV NTA count (*n* = 10 per experimental group). NTA analysis was carried out using the NTA software (version 3, Malvern Panalytical, UK), and is based on 4 reads per sample and presented as an average read from the samples (black line), with the standard deviation represented as a red line. Histograms for NTA analysis are based on NTA results from 10 samples per experimental group (normoxia vs. hypoxia). Statistical significance was regarded as *p* < 0.05. STRING analysis was carried out with medium confidence in STRING (https://string-db.org/, accessed on 10 March 2022).

## 5. Conclusions

This study used the naked mole-rat model to assess CNS-related responses of PADs in hypoxic protection/tolerance and identify whether circulating EV signatures could reveal a fingerprint for whole-body hypoxia-tolerant responses. Furthermore, citrullination-specific signatures in EVs were assessed in animals under normal versus hypoxic conditions. Our findings highlight novel roles for PADs in regulating brain-associated responses to acute hypoxia challenge and modifications in circulatory EV proteome signatures, possibly indicating a shift to re-directing resources systematically in response to acute hypoxic challenge in this hypoxia-tolerant species.

## Figures and Tables

**Table 1:** Total proteome of plasma EVs under normoxia and hypoxia conditions. Shared hits are listed at the beginning of the table, blue highlighted rows are hits identified only in normoxia EVs, and pink highlighted rows are hits only identified in hypoxia.

Protein ID	Protein Name	Protein ID	Protein Name
	**PROTEIN HITS SHARED BETWEEN NORMOXIA AND HYPOXIA EV PROTEOMES:**		
G5AZB7	Apolipoprotein B-100	G5AUT5	Ficolin-3
G5C0N5	Complement C3	G5B491	Alpha-1-antichymotrypsin
G5BPM1	Alpha-2-macroglobulin	G5B5P1	Alpha-fetoprotein
G5B5P2	Serum albumin (fragment)	G5C4R4	Complement component C7
G5C3H6	Complement C4-A	G5BNV2	Plasma kallikrein
G5BQA9	Serotransferrin	G5BM72	Coagulation factor XIII B chain
G5BM71	Complement factor H (fragment)	G5BXY1	Hemoglobin subunit alpha
G5AXS5	Complement C5	G5BHR4	Fibronectin
G5BC53	Plasminogen	G5BJ39	Keratin, type II cytoskeletal 8
G5BUN4	Inter-alpha-trypsin inhibitor heavy chain H4	G5AZM7	Alpha-amylase
G5AXS0	Gelsolin	G5AQM1	Complement factor I
G5BT86	Kininogen-1	G5BJ37	Keratin, type II cytoskeletal 79
G5ALS1	Keratin, type II cytoskeletal 6B	G5C4H7	Mannose-binding protein A
G5BSL1	Ceruloplasmin	G5AXV0	Catalase
G5BE53	Vitamin D-binding protein	G5BI78	Actin, cytoplasmic 1
G5BQB0	Inhibitor of carbonic anhydrase	G5BYJ8	Hemoglobin subunit beta
G5B0M6	Keratin, type I cytoskeletal 14	G5AV68	Vitamin K-dependent protein S
G5CBM7	Apolipoprotein E	G5BT83	Adiponectin
G5ALS3	Keratin, type II cytoskeletal 5	G5CAS5	Complement C1s subcomponent
G5C3H1	Complement factor B (fragment)	G5BYX0	Peroxiredoxin-2 isoform a
G5BQH5	Apolipoprotein A-I	G5ATW7	Carbonic anhydrase 2
G5BUN3	Inter-alpha-trypsin inhibitor heavy chain H3	G5CAP7	Glyceraldehyde-3-phosphate dehydrogenase
G5BQI5	Angiotensinogen	G5C3E9	Apolipoprotein M
G5BT87	Histidine-rich glycoprotein	G5ATW9	Carbonic anhydrase 1
G5BUN2	Inter-alpha-trypsin inhibitor heavy chain H1	G5BKR5	Thyroxine-binding globulin
G5ARS6	Antithrombin-III (fragment)	G5BJV7	Adipocyte plasma membrane-associated protein
G5B0M4	Keratin, type I cytoskeletal 17	G5BAT4	Desmoplakin
G5BQH3	Apolipoprotein A-IV	G5AYL7	Sulfhydryl oxidase
G5BBR0	Hemopexin	G5BHZ8	Complement C1q subcomponent subunit A
G5BML3	Fibrinogen beta chain	G5BIZ5	Anion exchange protein
G5AXV8	Inter-alpha-trypsin inhibitor heavy chain H2	G5C521	Mannan-binding lectin serine protease 2
G5ATC4	Prothrombin	G5AN09	Zinc-alpha-2-glycoprotein
G5BGY7	Beta-2-glycoprotein 1	G5BZF5	Hepatocyte growth factor activator
G5BML2	Fibrinogen alpha chain (fragment)	G5CBM5	Apolipoprotein C-I
G5CB46	Coagulation factor V	G5BNG0	Platelet-activating factor acetylhydrolase
G5BP10	C4b-binding protein (fragment)	G5BY64	Insulin-like growth factor-binding protein complex acid labile chain
G5BT89	Alpha-2-HS-glycoprotein	G5BHZ6	Complement C1q subcomponent subunit B
G5BT88	Fetuin-B	G5AVW1	Coagulation factor X
G5BLJ5	Plasma protease C1 inhibitor	G5BHZ7	Complement C1q subcomponent subunit C
G5BVN8	Vitronectin	G5CA61	Four and a half LIM domains protein 1
G5BML1	Fibrinogen gamma chain	G5ANE0	Complement component C9
G5BS33	Hemoglobin subunit beta	G5BWM1	Pentaxin
G5B7K8	Alpha-1B-glycoprotein	G5BHB1	Pyruvate kinase
G5B1Y4	Protein AMBP	G5BTJ5	Cysteine-rich secretory protein 3
G5B496	Alpha-1-antiproteinase S	G5BKU0	Creatine kinase M-type
G5BCV1	Alpha-2-antiplasmin	G5BX50	Putative carboxypeptidase PM20D1
G5B7P8	Phospholipid transfer protein	G5B183	Putative hexokinase HKDC1
G5BBS9	Annexin	G5B8W4	40S ribosomal protein S27a
G5BLD9	Myelin proteolipid protein	G5C5Z0	Serine/threonine-protein kinase 11
G5C9Y4	E3 ubiquitin-protein ligase HUWE1	G5BMW1	Transmembrane protein 127
G5C1U0	Protein FAM184A	G5B165	Multidrug resistance-associated protein 4
G5BQ09	Coagulation factor XII	G5BL81	ATP synthase subunit alpha
G5AU87	General transcription factor IIE subunit 1	G5AW28	ATP-binding cassette sub-family D member 3
G5AS67	Arf-GAP with Rho-GAP domain, ANK repeat and PH domain-containing protein 1	G5BZJ1	Fer3-like protein
G5BB67	ATP synthase subunit beta	G5B253	Ventricular zone-expressed PH domain-containing protein-like protein 1
G5AVU8	Growth arrest-specific protein 6	G5BR57	Leucine-rich repeat flightless-interacting protein 2
	**PROTEIN HITS IDENTIFIED IN NORMOXIA PLASMA EVS ONLY:**		
		G5BKL3	Histone H4
G5B3P5	Keratin, type II cytoskeletal 75	G5AKU3	Ataxin-2
G5CAA1	Aminopeptidase	G5C4Z8	Vacuolar protein sorting-associated protein 13D
G5BM62	Thrombospondin-1	G5C6Q5	Thyroglobulin
G5AKA3	L-lactate dehydrogenase	G5C307	Transthyretin
G5BL96	Keratin, type II cytoskeletal 7	G5BAK9	Fatty acid-binding protein, epidermal
G5B1Y7	Alpha-1-acid glycoprotein 1 (fragment)	G5C1Y6	Protein FAM161A
G5B3Z0	Xanthine dehydrogenase/oxidase	G5ANL3	Serum paraoxonase/arylesterase 1
G5C682	Phosphatidylcholine-sterol acyltransferase	G5C8M3	Malate dehydrogenase
G5AU84	Beta-actin-like protein 2	G5C311	Desmoglein-1
E3VX78	Transferrin receptor protein 1	G5B899	Cytosolic phospholipase A2 gamma (fragment)
G5BTD5	Mannan-binding lectin serine protease 1	G5CB14	Ribonucleoside-diphosphate reductase subunit M2
G5AXJ7	Protein Z-dependent protease inhibitor	G5C3J5	Aquaporin-1 isoform 1
G5CAS6	Complement C1r subcomponent	G5BJ09	Glial fibrillary acidic protein
G5BDQ8	Vascular non-inflammatory molecule 3 (fragment)	G5C4R3	Complement component C6
G5BSJ5	Lumican	G5BUL8	Dynein heavy chain 1, axonemal
G5ALX2	Catenin beta-1	G5C8Z7	Midasin
G5C0E1	Nesprin-1	G5BDF7	Secretory carrier-associated membrane protein
G5ALE6	Leucine-rich repeat-containing protein 15	G5BUP0	Transketolase
G5CAR5	Triosephosphate isomerase	G5BLQ4	Oxysterol-binding protein
G5BRD7	Sarcoplasmic/endoplasmic reticulum calcium ATPase 1	G5B5A1	Disks large homolog 4 isoform 1
G5C9X6	Flavin reductase (fragment)		
G5B6P6	Coiled-coil domain-containing protein 88B	G5C4J5	Crossover junction endonuclease EME1
G5BIX4	E3 ubiquitin-protein ligase topors	G5AZ29	Inversin
G5BJ98	Coiled-coil domain-containing protein 57		
G5AQB5	PTB domain-containing engulfment adapter protein 1	G5AKJ8	Tubulin alpha chain
G5C1E5	Carboxypeptidase B2	G5BP51	Eukaryotic translation initiation factor 4E type 1B
	**PROTEIN HITS IDENTIFIED IN HYPOXIA PLASMA EVS ONLY:**		
G5ALS8	Keratin, type II cytoskeletal 1 (fragment)	G5AQW0	Envoplakin
G5B0N0	Keratin, type I cytoskeletal 13	G5BSI2	FYVE, RhoGEF, and PH domain-containing protein 4
G5ATW7	Carbonic anhydrase 2 (fragment)	G5AKF7	Transforming acidic coiled-coil-containing protein 2
G5ALS4	Keratin, type II cytoskeletal 71	G5AZ97	Triple functional domain protein
G5BV28	Histone H3	G5C0I8	Sarcalumenin
G5AX68	Keratin, type I cytoskeletal 27	G5B7I2	Pre-mRNA-splicing factor SYF1
G5BSE8	Histone H2A	G5C2U0	Piezo-type mechanosensitive ion channel component
G5BH20	Histone H2B	G5BAY8	Lysine-specific demethylase 5C
G5B251	Heat shock cognate 71 kDa protein	G5APP5	Complement component C8 beta chain
G5AV43	Biliverdin reductase A	G5AVH0	Transient receptor potential cation channel subfamily M member 3
G5AU24	Tubulin alpha-1C chain	G5BUY9	Skin-specific protein 32
G5ALK7	Elongation factor 1-alpha	G5BJ90	Leucine-rich repeat-containing protein 45
G5B5C8	Eukaryotic initiation factor 4A-I	G5BF75	Disks large-associated protein 5
G5AMU8	Histone-lysine N-methyltransferase	G5BAS8	Coagulation factor XIII A chain (fragment)
G5BEK1	14-3-3 protein theta	G5BPP6	Aconitate hydratase, mitochondrial
G5BSY3	Zinc finger C3H1 domain-containing protein	G5BHM2	Coatomer subunit delta
G5B0M0	Junction plakoglobin	G5B9Y3	Stress-70 protein, mitochondrial
G5BN83	MAGUK p55 subfamily member 3	G5BZV1	Inosine-5’-monophosphate dehydrogenase
G5BUZ5	Uncharacterized protein	G5C0H1	E3 ubiquitin-protein ligase UBR5 (fragment)
G5B8I9	Ficolin-1	G5AMK2	Transmembrane protein 176B
G5BRJ4	Induced myeloid leukemia cell differentiation protein Mcl-1-like protein		

**Table 2:** (A.) KEGG pathways and (B.) STRING pathways for total proteomic content of plasma EVs from naked mole-rats treated in normoxic and hypoxic conditions, respectively, as assessed by LC-MS/MS analysis. A tick (V) indicates whether the pathway was identified for the EV protein cargo in the respective experimental group; the observed gene count for the pathway is indicated in brackets; blue highlighted rows indicate pathways identified in the normoxia group only, and pink highlighted pathways indicate pathways identified in the hypoxia group only.

**A. KEGG Pathways**	**Normoxia** **(Observed Gene Count)**	**Hypoxia** **(Observed Gene Count)**
Complement and coagulation cascades	V (31)	V (28)
*Staphylococcus aureus* infection	V (17)	V (16)
Cholesterol metabolism	V (7)	V (6)
Pertussis	V (8)	V (7)
Vitamin digestion and absorption	V (3)	V (3)
Systemic lupus erythematosus	V (12)	V (12)
African trypanosomiasis	V (4)	V (4)
Arrhythmogenic right ventricular cardiomyopathy	V (4)	V (4)
Chagas disease	V (5)	V (5)
Prion disease	V (10)	V (10)
Proteoglycan in cancer	V (6)	
Proximal tubule bicarbonate reclamation	V (4)	
Ferroptosis	V (4)	
Glycolysis/gluconeogenesis	V (4)	
HIF-1 signalling pathway	V (6)	
Thyroid hormone synthesis	V (4)	
Phagosome	V (7)	
Carbon metabolism	V (6)	
**B. STRING pathways**	**Normoxia** **(Observed Gene Count)**	**Hyopxia** **(Observed Gene Count)**
Complement and coagulation cascades, and serine-type endopeptidase inhibitor activity	V (46)	V (43)
Complement and coagulation cascades, and serpin (serine protease inhibitor)	V (21)	V (19)
Complement and coagulation cascades, and inter-alpha-trypsin inhibitor heavy chain, C-terminal	V (19)	V (17)
Complement activation, alternative pathway, and factor i/membrane attack complex	V (3)	V (3)
Complement activation	V (18)	V (17)
Complement activation, lectin pathway	V (4)	V (3)
Membrane attack complex	V (4)	V (4)
Synapse pruning, and complement c1r subcomponent	V (5)	V (4)
Mixed, incl. fibrinolysis and inter-alpha-trypsin inhibitor heavy chain, C-terminal	V (13)	V (11)
Mixed, incl. fibrinogen alpha/beta chain family and inter-alpha-trypsin inhibitor heavy chain, C-terminal	V (13)	V (10)
Mixed, incl. fibrinogen alpha/beta chain family and protein AMBP	V (6)	V (6)
Mixed, incl. transferrin and hemopexin	V (7)	V (7)
Mixed, incl. hemopexin and haptoglobin	V (3)	V (4)
Mixed, incl. cholesterol metabolism and regulation of lipoprotein lipase activity	V (7)	V (7)
Cholesterol metabolism and regulation of lipoprotein lipase activity	V (6)	V (6)
Cholesterol efflux and triglyceride transport	V (5)	V (5)
Intermediate filament protein, conserved site	V (4)	V (5)
Carbonic anhydrase, alpha-class, conserved site, and glycophorin A	V (3)	V (3)
Intermediate filament protein	V (5)	V (8)
High-density lipoprotein particle	V (3)	V (3)
Carbon metabolism and phosphoglycerate mutase family	V (6)	
Intermediate filament and apical plasma membrane urothelial plaque	V (6)	
Pentose phosphate pathway and glycolytic process	V (4)	
Mixed, incl. apple domain and antithrombin-iii	V (3)	
Mixed, incl. HMW kininogen and mitochondrial glycoprotein	V (3)	
Keratin type II head and keratin type I		V (3)
Mixed, incl. blood coagulation, fibrin clot formation, and alpha2-antiplasmin		V (3)
Intermediate filament protein, conserved site, and keratinocyte activation		V (6)

**Table 3:** F95-enriched proteins identified in plasma EVs from naked mole-rats treated under normoxic and hypoxic conditions, as assessed by LC-MS/MS analysis. Common and specific hits per group are indicated in the table; a tick (V) indicates whether the deiminated protein hit was identified in normoxia or hypoxia plasma EVs, or in both; blue highlighted rows indicate hits identified in the normoxia group only, and pink highlighted rows indicate hits identified in the hypoxia group only.

Protein ID	Protein Name	Normoxia	Hypoxia
G5B5P2	Serum albumin	V	V
G5BT87	Histidine-rich glycoprotein	V	V
G5ALS3	Keratin, type II cytoskeletal	V	V
G5ALS1	Keratin, type II cytoskeletal 6B	V	V
G5B3Q0	Keratin, type II cuticular Hb5	V	V
G5BPM1	Alpha-2-macroglobulin	V	V
G5BT86	Kininogen-1	V	V
G5BS33	Hemoglobin subunit beta	V	V
G5BQA9	Serotransferrin	V	V
G5B0M6	Keratin, type I cytoskeletal 14	V	V
G5BXY1	Hemoglobin subunit alpha	V	V
G5BJ37	Keratin, type II cytoskeletal 79	V	V
G5BAT4	Desmoplakin	V	V
G5C776	Arginase	V	V
G5B0M4	Keratin, type I cytoskeletal 17	V	V
G5BJ39	Keratin, type II cytoskeletal 8	V	V
G5BYJ8	Hemoglobin subunit beta	V	V
G5BUN4	Inter-alpha-trypsin inhibitor heavy chain H4	V	V
G5C0N5	Complement C3	V	V
G5BSE8	Histone H2A	V	V
G5BV28	Histone H3	V	V
G5C3H6	Complement C4-A	V	V
G5BRJ4	Induced myeloid leukemia cell differentiation protein Mcl-1-like protein	V	V
G5C311	Desmoglein-1	V	V
G5ARW1	Peroxiredoxin-1	V	V
G5AWC0	Annexin	V	V
G5AXH0	Actin, gamma-enteric smooth muscle	V	V
G5BFU9	N6-adenosine-methyltransferase 70 kDa subunit	V	V
G5BG61	SRRM2-like protein	V	V
G5B0N6	Keratin, type I cuticular Ha3-I	V	
G5B0N5	Keratin, type I cuticular Ha4	V	
G5B3P5	Keratin, type II cytoskeletal 75	V	
G5AX68	Keratin, type I cytoskeletal 27	V	
G5B0N2	Keratin, type I cuticular Ha5	V	
G5ALS4	Keratin, type II cytoskeletal 71	V	
G5B3P8	Keratin, type II cuticular Hb4	V	
G5BL99	Keratin, type II cuticular Hb6	V	
G5BJ40	Keratin, type I cytoskeletal 18	V	
G5AX70	Keratin, type I cytoskeletal 25	V	
G5AYD5	Dedicator of cytokinesis protein 10	V	
G5BQ09	Coagulation factor XII	V	
G5AYL5	Integrator complex subunit 7	V	
G5B319	Janus kinase and microtubule-interacting protein 1	V	
G5B3A4	Plakophilin-1	V	
G5BV47	Histone H3.3	V	
G5BI06	Basement membrane-specific heparan sulfate proteoglycan core protein	V	
G5BUY9	Skin-specific protein 32	V	
G5C656	Puratrophin-1	V	
G5APA7	Selenoprotein P	V	
G5BH20	Histone H2B	V	
G5BKL1	Histone H2B		V
G5ALS8	Keratin, type II cytoskeletal 1 (fragment)		V
G5ALS9	Keratin, type II cytoskeletal 1b (fragment)		V
G5B0M0	Junction plakoglobin		V
G5CAP7	Glyceraldehyde-3-phosphate dehydrogenase (fragment)		V
G5C5U7	Plectin-1		V
G5BYG4	Apoptosis facilitator Bcl-2-like protein 14		V
G5BNM3	Multidrug resistance-associated protein 7		V
G5BAK9	Fatty acid-binding protein, epidermal		V
G5BX43	Serine/threonine-protein kinase PCTAIRE-3		V
G5C312	Desmocollin-1 (fragment)		V
G5AQ00	Tubulin alpha-1C chain		V
G5AUQ5	Serine/threonine-protein kinase PDIK1L		V
G5BYF7	Putative G-protein coupled receptor 19		V
G5B253	Ventricular zone-expressed PH domain-containing protein-like protein 1		V

**Table 4:** EV citrullinome pathway analysis of normoxia- and hypoxia-treated naked mole-rats. (A.) KEGG pathways and (B.) local network cluster STRING pathways for F95-enriched (citrullinome) proteomic content of plasma EVs from of normoxia- and hypoxia-treated naked mole-rats, as assessed by LC-MS/MS analysis. A tick (V) indicates whether the pathway was identified for EV citrullinome protein cargo hits in the respective (normoxia, hypoxia) group; blue highlights pathways identified only in the normoxia, and pink highlights pathways identified only in the hypoxia group.

A. KEGG Pathways F95-Enriched EV Cargo	Normoxia (Observed Gene Count)	Hypoxia (Observed Gene Count)
Complement and coagulation cascades	V (5)	V (4)
*Staphylococcus aureus* infection	V (8)	V (4)
African trypanosomiasis	V (3)	V (3)
Systemic lupus erythematosus	V (4)	V (4)
Oestrogen signalling pathway	V (5)	NA
**B. STRING Pathways F95-Enriched EV cargo**	**Normoxia** **(Observed Gene Count)**	**Hypoxia** **(Observed Gene Count)**
Intermediate filament and apical plasma membrane urothelial plaque	V (13)	
Intermediate filament protein, conserved site, and keratinocyte activation	V (7)	
Keratin, type I cytoskeletal 18 and keratin, type II head	V (2)	
Keratin, type I and structural constituent of skin epidermis	V (2)	
Intermediate filament protein, conserved site	V (4)	V (5)
Intermediate filament protein	V (12)	V (6)
Keratin, type II head and keratin, type I	V (2)	V (4)
Complement and coagulation cascades, and serine-type endopeptidase inhibitor activity	V (6)	V (6)
Complement and coagulation cascades, and inter-alpha-trypsin inhibitor heavy chain, C-terminal	V (4)	V (3)
Mixed, incl. hmw kininogen and mitochondrial glycoprotein	V (3)	V (2)
Haemoglobin complex	V (2)	V (2)
Desmosome	V (3)	V (2)
Desmosome and cell–cell adhesion mediated by cadherin		V (3)

**Table 5:** Local network cluster STRING networks for all F95-enriched proteins identified in naked mole-rat brains following normoxia and hypoxia treatment. A tick (**V**) indicates whether the pathway was identified in the respective group; normoxia-specific pathways are highlighted in blue and hypoxia-specific pathways in pink. A full list of the protein hits underlying the network analysis is provided for both groups in Supplementary Tables S1 and S2.

STRING NETWORKS IN NORMOXIA AND HYPOXIA BRAINS (All F95 Hits)	NORMOXIA (Observed Gene Count)	HYPOXIA (Observed Gene Count)
Carbon metabolism and pyruvate	**V** (37)	**V** (43)
Carbon metabolism and phosphoglycerate mutase family	**V** (33)	**V** (39)
Mixed, incl. oxidative phosphorylation and apoptosis-multiple species	**V** (32)	**V** (33)
Citrate cycle (TCA cycle), lactate/malate dehydrogenase, NAD-binding domain	**V** (18)	**V** (22)
Citrate cycle (TCA cycle) and cysteine and methionine metabolism	**V** (19)	**V** (25)
Oxidative phosphorylation and cytochrome c oxidase subunit VIa	**V** (24)	**V** (31)
Mixed, incl. oxidative phosphorylation and uncharacterised protein family (upf0240)	**V** (26)	**V** (33)
Ubiquinone and oxidative phosphorylation	**V** (17)	**V** (22)
Citrate cycle (TCA cycle)	**V** (14)	**V** (11)
Oxidative phosphorylation and cytochrome c oxidase subunit VIIc	**V** (18)	**V** (24)
Pentose phosphate pathway and phosphoglycerate mutase family	**V** (14)	**V** (14)
Ubiquinone and MNLL subunit	**V** (12)	**V** (16)
Ubiquinone	**V** (11)	**V** (15)
Mixed, incl. spectrin repeat and immunoglobulin i-set	**V** (12)	**V** (13)
Glycolysis/gluconeogenesis and 6-phosphofructo-2-kinase	**V** (11)	**V** (11)
Mixed, incl. spectrin repeat and ankyrin, UPA domain	**V** (11)	**V** (11)
Collecting duct acid secretion and V-ATPase subunit H	**V** (7)	**V** (8)
Mixed, incl. atp1g1/plm/mat8 family, and endocrine and other factor-regulated calcium reabsorption	**V** (10)	**V** (11)
Ribosome	**V** (14)	**V** (15)
GroEL-like equatorial domain superfamily	**V** (7)	
Proteasome		**V** (14)
Ribosome and ribosomal protein L23		**V** (25)
Ubiquinone and zinc-finger domain		**V** (9)
Mixed, incl. regulation of actin cytoskeleton and actin		**V** (16)
Septin		**V** (7)

**Table 6:** KEGG pathways for all F95-enriched proteins in naked mole-rat brains following normoxia or hypoxia treatment. A tick (**V**) indicates whether the pathway was identified; the observed gene count for each pathway is indicated in brackets. Pathways identified in normoxia brains only are highlighted in blue, and those identified in hypoxia brains only in pink. A full list of the protein hits underlying the network analysis is provided for both groups in Supplementary Tables S1 and S2.

KEGG PATHWAYS IN NORMOXIA AND HYPOXIA BRAINS (All F95 Hits)	NORMOXIA (Observed Gene Count)	HYPOXIA (Observed Gene Count)
Metabolic pathways	**V** (115)	**V** (152)
Carbon metabolism	**V** (39)	**V** (47)
Prion disease	**V** (51)	**V** (71)
Parkinson’s disease	**V** (47)	**V** (65)
Huntington’s disease	**V** (48)	**V** (67)
Alzheimer’s disease	**V** (51)	**V** (72)
Oxidative phosphorylation	**V** (31)	**V** (39)
Amyotrophic lateral sclerosis	**V** (47)	**V** (64)
Synaptic vesicle cycle	**V** (22)	**V** (22)
Citrate cycle (TCA cycle)	**V** (17)	**V** (21)
Endocrine and other factor-regulated calcium reabsorption	**V** (18)	**V** (22)
Glycolysis/gluconeogenesis	**V** (19)	**V** (20)
Biosynthesis of amino acids	**V** (17)	**V** (20)
Retrograde endocannabinoid signalling	**V** (21)	**V** (30)
Thermogenesis	**V** (25)	**V** (33)
Endocytosis	**V** (26)	**V** (29)
Pyruvate metabolism	**V** (13)	**V** (14)
Non-alcoholic fatty liver disease	**V** (21)	**V** (26)
cGMP-PKG signalling pathway	**V** (21)	**V** (28)
Bacterial invasion of epithelial cells	**V** (15)	**V** (15)
Cardiac muscle contraction	**V** (15)	**V** (15)
HIF-1 signalling pathway	**V** (17)	**V** (19)
Glutamatergic synapse	**V** (17)	**V** (22)
Adrenergic signalling in cardiomyocytes	**V** (18)	**V** (21)
Spinocerebellar ataxia	**V** (18)	**V** (29)
Phagosome	**V** (17)	**V** (19)
Proximal tubule bicarbonate reclamation	**V** (8)	**V** (9)
Mineral absorption	**V** (11)	**V** (12)
Pancreatic secretion	**V** (14)	**V** (19)
Propanoate metabolism	**V** (9)	**V** (12)
Ribosome	**V** (16)	**V** (21)
Central carbon metabolism in cancer	**V** (11)	**V** (15)
Pentose phosphate pathway	**V** (8)	**V** (8)
Regulation of actin cytoskeleton	**V** (18)	**V** (19)
GABAergic synapse	**V** (12)	**V** (14)
Fc gamma R-mediated phagocytosis	**V** (12)	**V** (11)
Salivary secretion	**V** (11)	**V** (17)
Glyoxylate and dicarboxylate metabolism	**V** (8)	**V** (12)
Calcium signalling pathway	**V** (17)	**V** (26)
Cysteine and methionine metabolism	**V** (9)	**V** (10)
Thyroid hormone signalling pathway	**V** (13)	**V** (17)
Collecting duct acid secretion	**V** (7)	**V** (8)
cAMP signalling pathway	**V** (17)	**V** (20)
Adherens junction	**V** (10)	**V** (10)
2-Oxocarboxylic acid metabolism	**V** (6)	**V** (9)
Dopaminergic synapse	**V** (13)	**V** (21)
Gastric acid secretion	**V** (10)	**V** (14)
Aldosterone synthesis and secretion	**V** (11)	**V** (16)
Starch and sucrose metabolism	**V** (7)	**V** (8)
Focal adhesion	**V** (16)	**V** (18)
Long-term depression	**V** (9)	**V** (15)
Axon guidance	**V** (15)	**V** (14)
Leukocyte transendothelial migration	**V** (12)	**V** (13)
Arginine biosynthesis	**V** (6)	**V** (6)
Aldosterone-regulated sodium reabsorption	**V** (7)	**V** (7)
Alanine, aspartate, and glutamate metabolism	**V** (7)	**V** (7)
Insulin secretion	**V** (10)	**V** (13)
Valine, leucine, and isoleucine degradation	**V** (8)	**V** (13)
Butanoate metabolism	**V** (6)	**V** (6)
Circadian entrainment	**V** (10)	**V** (15)
Thyroid hormone synthesis	**V** (9)	**V** (13)
Necroptosis	**V** (12)	**V** (13)
Long-term potentiation	**V** (8)	**V** (14)
Glucagon signalling pathway	**V** (10)	**V** (17)
Salmonella infection	**V** (15)	**V** (23)
Spliceosome	**V** (11)	**V** (14)
Gap junction	**V** (9)	**V** (16)
Morphine addiction	**V** (9)	**V** (12)
Inositol phosphate metabolism	**V** (8)	**V** (7)
Oocyte meiosis	**V** (10)	**V** (17)
Tight junction	**V** (12)	**V** (15)
Phosphatidylinositol signalling system	**V** (9)	**V** (11)
VEGF signalling pathway	**V** (7)	**V** (7)
Arrhythmogenic right ventricular cardiomyopathy	**V** (8)	**V** (8)
Aminoacyl-tRNA biosynthesis	**V** (6)	**V** (9)
Tryptophan metabolism	**V** (6)	**V** (9)
Lysine degradation	**V** (7)	**V** (10)
Rap1 signalling pathway	**V** (13)	**V** (16)
Yersinia infection	**V** (10)	**V** (13)
Viral carcinogenesis	**V** (12)	**V** (17)
Proteasome	**V** (6)	**V** (14)
Arginine and proline metabolism	**V** (6)	**V** (9)
Fructose and mannose metabolism	**V** (5)	**V** (6)
Protein processing in endoplasmic reticulum	**V** (11)	**V** (16)
Human immunodeficiency virus 1 infection	**V** (12)	**V** (16)
Glutathione metabolism	**V** (6)	**V** (9)
Proteoglycans in cancer	**V** (12)	**V** (17)
Oxytocin signalling pathway	**V** (10)	**V** (18)
African trypanosomiasis	**V** (5)	**V** (7)
Synthesis and degradation of ketone bodies	**V** (3)	**V** (3)
Carbohydrate digestion and absorption	**V** (5)	**V** (7)
Serotonergic synapse	**V** (8)	**V** (15)
Hippo signalling pathway	**V** (10)	**V** (14)
Cholinergic synapse	**V** (8)	**V** (14)
Ferroptosis	**V** (5)	**V** (6)
Vasopressin-regulated water reabsorption	**V** (5)	**V** (10)
Amphetamine addiction	**V** (6)	**V** (9)
Rheumatoid arthritis	**V** (7)	**V** (8)
Cell adhesion molecules	**V** (9)	**V** (11)
Galactose metabolism	**V** (4)	**V** (5)
Legionellosis	**V** (6)	**V** (8)
Tuberculosis	**V** (10)	**V** (13)
Sphingolipid signalling pathway	**V** (8)	**V** (11)
Beta-alanine metabolism	**V** (4)	**V** (6)
Phospholipase D signalling pathway	**V** (9)	**V** (11)
Melanogenesis	**V** (7)	**V** (11)
Human cytomegalovirus infection	**V** (11)	**V** (16)
D-Glutamine and D-glutamate metabolism	**V** (2)	**V** (2)
Antigen processing and presentation	**V** (5)	**V** (7)
Pathways in cancer	**V** (20)	**V** (27)
Hepatitis C	**V** (9)	**V** (16)
Human papillomavirus infection	**V** (14)	**V** (18)
Ras signalling pathway	**V** (11)	**V** (14)
RNA transport	**V** (9)	**V** (13)
Phenylalanine, tyrosine, and tryptophan biosynthesis	**V** (2)	
Nitrogen metabolism	**V** (3)	
Mineral absorption		**V** (12)
Estrogen signalling pathway		**V** (16)
Renin secretion		**V** (9)
Platelet activation		**V** (12)
Bile secretion		**V** (10)
Apelin signalling pathway		**V** (12)
Fatty acid degradation		**V** (6)
Vascular smooth muscle contraction		**V** (11)
Amino sugar and nucleotide sugar metabolism		**V** (6)
GnRH signalling pathway		**V** (9)
mRNA surveillance pathway		**V** (9)
MAPK signalling pathway		**V** (18)
Human T-cell leukemia virus 1 infection		**V** (15)
Chemokine signalling pathway		**V** (13)
GnRH secretion		**V** (7)
Inflammatory mediator regulation of TRP channels		**V** (9)
Parathyroid hormone synthesis, secretion, and action		**V** (9)
Cellular senescence		**V** (11)
Renal cell carcinoma		**V** (7)
Apoptosis		**V** (11)
Alcoholism		**V** (11)
Insulin signaling pathway		**V** (10)
Histidine metabolism		**V** (4)
Cushing syndrome		**V** (11)
Growth hormone synthesis, secretion, and action		**V** (9)
Purine metabolism		**V** (10)
Fatty acid metabolism		**V** (6)
Hepatitis B		**V** (11)

**Table 7:** KEGG pathways for F95 hits only identified in hypoxia brains (and not overlapping with the normoxia group). The 50 KEGG pathways are listed below that relate to the protein network built based on F95 hits in hypoxia brain only (shown in Figure 8B); the observed gene count for each pathway is listed; (*) are unique to this list only.

KEGG Pathways for F95-Specific Hits Identified in Hypoxia Brains Only	Observed Gene Count	KEGG Pathways for F95-Specific Hits Identified in Hypoxia Brains Only	Observed Gene Count
Metabolic pathways	36	Renin secretion	5
Gap junction	9	Endocytosis	9
Long-term depression	8	Focal adhesion	8
Ribosome	10	Amyotrophic lateral sclerosis	11
Prion disease	13	Aminoacyl-tRNA biosynthesis	4
Oestrogen signalling pathway	9	Vasopressin-regulated water reabsorption	4
Huntington’s disease	13	Growth hormone synthesis, secretion, and action	6
Salmonella infection	11	Insulin secretion	5
Parkinson’s disease	11	Yersinia infection	6
Dopaminergic synapse	8	GnRH signaling pathway	5
Valine, leucine, and isoleucine degradation	5	Spliceosome	6
Retrograde endocannabinoid signalling	8	Melanogenesis	5
Serotonergic synapse	7	Choline metabolism in cancer *	5
Oxytocin signalling pathway	8	Beta-alanine metabolism	3
Carbon metabolism	7	Glucagon signalling pathway	5
Glyoxylate and dicarboxylate metabolism	4	Long-term potentiation	4
Protein processing in endoplasmic reticulum	8	Parathyroid hormone synthesis, secretion, and action	5
Platelet activation	7	Non-alcoholic fatty liver disease	6
Salivary secretion	6	Rap1 signalling pathway	7
Alzheimer’s disease	12	Amphetamine addiction	4
Oxidative phosphorylation	7	Propanoate metabolism	3
Circadian entrainment	6	Sulphur metabolism *	2
Vascular smooth muscle contraction	7	Glutamatergic synapse	5
GnRH secretion	5	Cholinergic synapse	5
Prostate cancer *	6	Central carbon metabolism in cancer	4

## Data Availability

The data supporting the study are enclosed in the manuscript and the Supporting Materials section.

## Supplementary Materials

The following supporting information can be downloaded at https://www.mdpi.com/article/10.3390/ijms23094683/s1.

## References

[1] Buffenstein R., Amoroso V., Andziak B., Avdieiev S., Azpurua J., Barker A.J., Bennett N.C., Brieno-Enriquez M.A., Bronner G.N., Coen C. (2022). The naked truth: A comprehensive clarification and classification of current ‘myths’ in naked mole-rat biology. Biol. Rev. Camb. Philos. Soc..

[2] Pamenter M.E. (2022). Adaptations to a hypoxic lifestyle in naked mole-rats. J. Exp. Biol..

[3] Chung D., Dzal Y.A., Seow A., Milsom W.K., Pamenter M.E. (2016). Naked mole rats exhibit metabolic but not ventilatory plasticity following chronic sustained hypoxia. Proc. Biol. Sci..

[4] Pamenter M.E., Dzal Y.A., Milsom W.K. (2015). Adenosine receptors mediate the hypoxic ventilatory response but not the hypoxic metabolic response in the naked mole rat during acute hypoxia. Proc. Biol. Sci..

[5] Pamenter M.E., Lau G.Y., Richards J.G., Milsom W.K. (2018). Naked mole rat brain mitochondria electron transport system flux and H(+) leak are reduced during acute hypoxia. J. Exp. Biol..

[6] Park T.J., Reznick J., Peterson B.L., Blass G., Omerbasic D., Bennett N.C., Kuich P.H.J.L., Zasada C., Browe B.M., Hamann W. (2017). Fructose-driven glycolysis supports anoxia resistance in the naked mole-rat. Science.

[7] Cheng H., Pamenter M.E. (2021). Naked mole-rat brain mitochondria tolerate in vitro ischaemia. J. Physiol..

[8] Pamenter M.E., Cheng H. (2022). Supermole-rat to the rescue: Does the naked mole-rat offer a panacea for all that ails us?. Comp. Biochem. Physiol. A Mol. Integr. Physiol..

[9] Pamenter M.E., Dzal Y.A., Thompson I.A., Milsom W.K. (2019). Do naked mole rats accumulate a metabolic acidosis or an oxygen debt in severe hypoxia?. J. Exp. Biol..

[10] Houlahan C.R., Kirby A.M., Dzal Y.A., Fairman G.D., Pamenter M.E. (2018). Divergent behavioural responses to acute hypoxia between individuals and groups of naked mole rats. Comp. Biochem. Physiol. B Biochem. Mol. Biol..

[11] Ilacqua A.N., Kirby A.M., Pamenter M.E. (2017). Behavioural responses of naked mole rats to acute hypoxia and anoxia. Biol. Lett..

[12] Kirby A.M., Fairman G.D., Pamenter M.E. (2018). Atypical behavioural, metabolic and thermoregulatory responses to hypoxia in the naked mole rat (*Heterocephalus glaber*). J. Zool..

[13] Cheng H., Sebaa R., Malholtra N., Lacoste B., El Hankouri Z., Kirby A., Bennett N.C., van Jaarsveld B., Hart D.W., Tattersall G.J. (2021). Naked mole-rat brown fat thermogenesis is diminished during hypoxia through a rapid decrease in UCP1. Nat. Commun..

[14] Vandewint A.L., Zhu-Pawlowsky A.J., Kirby A., Tattersall G.J., Pamenter M.E. (2019). Evaporative cooling and vasodilation mediate thermoregulation in naked mole-rats during normoxia but not hypoxia. J. Therm. Biol..

[15] Al-Attar R., Childers C.L., Nguyen V.C., Pamenter M.E., Storey K.B. (2020). Differential protein phosphorylation is responsible for hypoxia-induced regulation of the Akt/mTOR pathway in naked mole rats. Comp. Biochem. Physiol. A Mol. Integr. Physiol..

[16] Reznick J., Park T.J., Lewin G.R. (2021). A Sweet Story of Metabolic Innovation in the Naked Mole-Rat. Adv. Exp. Med. Biol..

[17] Farhat E., Devereaux M.E.M., Pamenter M.E., Weber J.M. (2020). Naked mole-rats suppress energy metabolism and modulate membrane cholesterol in chronic hypoxia. Am. J. Physiol. Regul. Integr. Comp. Physiol..

[18] Hadj-Moussa H., Chiasson S., Cheng H., Eaton L., Storey K.B., Pamenter M.E. (2021). MicroRNA-mediated inhibition of AMPK coordinates tissue-specific downregulation of skeletal muscle metabolism in hypoxic naked mole-rats. J. Exp. Biol..

[19] Hadj-Moussa H., Pamenter M.E., Storey K.B. (2021). Hypoxic naked mole-rat brains use microRNA to coordinate hypometabolic fuels and neuroprotective defenses. J. Cell Physiol..

[20] Logan S.M., Szereszewski K.E., Bennett N.C., Hart D.W., van Jaarsveld B., Pamenter M.E., Storey K.B. (2020). (2020). The brains of six African mole-rat species show divergent responses to hypoxia. J. Exp. Biol..

[21] Nguyen V.C., Deck C.A., Pamenter M.E. (2019). Naked mole-rats reduce the expression of ATP-dependent but not ATP-independent heat shock proteins in acute hypoxia. J. Exp. Biol..

[22] Dzal Y.A., Seow A., Borecky L.G., Chung D., Gill S.K.G., Milsom W.K., Pamenter M.E. (2019). Glutamatergic Receptors Modulate Normoxic but Not Hypoxic Ventilation and Metabolism in Naked Mole Rats. Front. Physiol..

[23] Cheng H., Munro D., Huynh K., Pamenter M.E. (2021). Naked mole-rat skeletal muscle mitochondria exhibit minimal functional plasticity in acute or chronic hypoxia. Comp. Biochem. Physiol. B Biochem. Mol. Biol..

[24] Lau G.Y., Milsom W.K., Richards J.G., Pamenter M.E. (2020). Heart mitochondria from naked mole-rats (*Heterocephalus glaber*) are more coupled, but similarly susceptible to anoxia-reoxygenation stress than in laboratory mice (*Mus musculus*). Comp. Biochem. Physiol. B Biochem. Mol. Biol..

[25] Siesjo B.K. (1988). Mechanisms of ischemic brain damage. Crit. Care Med..

[26] Farhat E., Devereaux M.E.M., Cheng H., Weber J.M., Pamenter M.E. (2021). Na(+)/K(+)-ATPase activity is regionally regulated by acute hypoxia in naked mole-rat brain. Neurosci. Lett..

[27] Buck L.T., Pamenter M.E. (2006). Adaptive responses of vertebrate neurons to anoxia--matching supply to demand. Respir. Physiol. Neurobiol..

[28] Choi D.W. (1992). Excitotoxic cell death. J. Neurobiol..

[29] Cheng H., Qin Y.A., Dhillon R., Dowell J., Denu J.M., Pamenter M.E. (2022). Metabolomic Analysis of Carbohydrate and Amino Acid Changes Induced by Hypoxia in Naked Mole-Rat Brain and Liver. Metabolites.

[30] Peterson B.L., Larson J., Buffenstein R., Park T.J., Fall C.P. (2012). Blunted neuronal calcium response to hypoxia in naked mole-rat hippocampus. PLoS ONE.

[31] Peterson B.L., Park T.J., Larson J. (2012). Adult naked mole-rat brain retains the NMDA receptor subunit GluN2D associated with hypoxia tolerance in neonatal mammals. Neurosci. Lett..

[32] Pamenter M.E., Uysal-Onganer P., Huynh K.W., Kraev I., Lange S. (2019). Post-Translational Deimination of Immunological and Metabolic Protein Markers in Plasma and Extracellular Vesicles of Naked Mole-Rat (*Heterocephalus glaber*). Int. J. Mol. Sci..

[33] Vossenaar E.R., Zendman A.J., van Venrooij W.J., Pruijn G.J. (2003). PAD, a growing family of citrullinating enzymes: Genes, features and involvement in disease. BioEssays.

[34] György B., Toth E., Tarcsa E., Falus A., Buzas E.I. (2006). Citrullination: A posttranslational modification in health and disease. Int. J. Biochem. Cell Biol..

[35] Alghamdi M., Al Ghamdi K.A., Khan R.H., Uversky V.N., Redwan E.M. (2018). An interplay of structure and intrinsic disorder in the functionality of peptidylarginine deiminases, a family of key autoimmunity-related enzymes. Cell Mol. Life Sci..

[36] Mondal S., Thompson P.R. (2019). Protein arginine deiminases (PADs): Biochemistry and chemical biology of protein citrullination. Acc. Chem. Res..

[37] Witalison E.E., Thompson P.R., Hofseth L.J. (2015). Protein Arginine Deiminases and Associated Citrullination: Physiological Functions and Diseases Associated with Dysregulation. Curr. Drug Targets.

[38] Wang S., Wang Y. (2013). Peptidylarginine deiminases in citrullination, gene regulation, health and pathogenesis. Biochim. Biophys. Acta.

[39] Lange S., Gallagher M., Kholia S., Kosgodage U.S., Hristova M., Hardy J., Inal J.M. (2017). Peptidylarginine Deiminases-Roles in Cancer and Neurodegeneration and Possible Avenues for Therapeutic Intervention via Modulation of Exosome and Microvesicle (EMV) Release?. Int. J. Mol. Sci..

[40] Lange S. (2021). Peptidylarginine deiminases and extracellular vesicles: Prospective drug targets and biomarkers in central nervous system diseases and repair. Neural Regen. Res..

[41] Wang L., Chen H., Tang J., Guo Z., Wang Y. (2022). Peptidylarginine Deiminase and Alzheimer’s Disease. J. Alzheimers Dis..

[42] Moscarello M.A., Pritzker L., Mastronardi F.G., Wood D.D. (2002). Peptidylarginine deiminase: A candidate factor in demyelinating disease. J. Neurochem..

[43] Lange S., Gögel S., Leung K.Y., Vernay B., Nicholas A.P., Causey C.P., Thompson P.R., Greene N.D., Ferretti P. (2011). Protein deiminases: New players in the developmentally regulated loss of neural regenerative ability. Dev. Biol..

[44] Lange S., Rocha-Ferreira E., Thei L., Mawjee P., Bennett K., Thompson P.R., Subramanian V., Nicholas A.P., Peebles D., Hristova M. (2014). Peptidylarginine deiminases: Novel drug targets for prevention of neuronal damage following hypoxic ischemic insult (HI) in neonates. J. Neurochem..

[45] Lazarus R.C., Buonora J.E., Flora M.N., Freedy J.G., Holstein G.R., Martinelli G.P., Jacobowitz D.M., Mueller G.P. (2015). Protein citrullination: A proposed mechanism for pathology in traumatic brain injury. Front. Neurol..

[46] Lange S., Wray S., Devine M., Matarin M., Hardy J., Nicholas A.P., Bhattacharya S.K. (2017). Protein deimination in protein misfolding disorders–modelled in human induced pluripotent stem cells (iPSCs). Protein Deimination in Human Health and Disease.

[47] Nicholas A.P., Lu L., Heaven M., Kadish I., van Groen T., Accavitti-Loper M.A., Wewering S., Kofskey D., Gambetti P., Brenner M., Nicholas A.P., Bhattacharya S.K. (2014). Ongoing studies of deimination in neurodegenerative diseases using the F95 antibody. Protein Deimination in Human Health and Disease.

[48] Ishigami A., Masutomi H., Handa S., Nakamura M., Nakaya S., Uchida Y., Saito Y., Murayama S., Jang B., Jeon Y.C. (2015). Mass spectrometric identification of citrullination sites and immunohistochemical detection of citrullinated glial fibrillary acidic protein in Alzheimer’s disease brains. J. Neurosci. Res..

[49] Jang B., Jeon Y.C., Shin H.Y., Lee Y.J., Kim H., Kondo Y., Ishigami A., Kim Y.S., Choi E.K. (2018). Myelin basic protein citrullination, a hallmark of central nervous system demyelination, assessed by novel monoclonal antibodies in prion diseases. Mol. Neurobiol..

[50] Attilio P.J., Flora M., Kamnaksh A., Bradshaw D.J., Agoston D., Mueller G.P. (2017). The effects of blast exposure on protein deimination in the brain. Oxid. Med. Cell Longev..

[51] Kosgodage U.S., Uysal-Onganer P., MacLatchy A., Kraev I., Chatterton N.P., Nicholas A.P., Inal J.M., Lange S. (2018). Peptidylarginine deiminases post-translationally deiminate prohibitin and modulate extracellular vesicle release and microRNAs in glioblastoma multiforme. Int. J. Mol. Sci..

[52] Uysal-Onganer P., MacLatchy A., Mahmoud R., Kraev I., Thompson P.R., Inal J.M., Lange S. (2020). Peptidylarginine deiminase isozyme-specific PAD2, PAD3 and PAD4 inhibitors differentially modulate extracellular vesicle signatures and cell invasion in two glioblastoma multiforme cell lines. Int. J. Mol. Sci..

[53] Caprariello A.V., Rogers J.A., Morgan M.L., Hoghooghi V., Plemel J.R., Koebel A., Tsutsui S., Dunn J.F., Kotra L.P., Ousman S.S. (2018). Biochemically altered myelin triggers autoimmune demyelination. Proc. Natl. Acad. Sci. USA.

[54] Faigle W., Cruciani C., Wolski W., Roschitzki B., Puthenparampil M., Tomas-Ojer P., Sellés-Moreno C., Zeis T., Jelcic I., Schaeren-Wiemers N. (2019). Brain citrullination patterns and T cell reactivity of cerebrospinal fluid-derived CD4+ T cells in multiple sclerosis. Front. Immunol..

[55] Sancandi M., Uysal-Onganer P., Kraev I., Mercer A., Lange S. (2020). Protein deimination signatures in plasma and plasma-EVs and protein deimination in the brain vasculature in a rat model of pre-motor Parkinson’s disease. Int. J. Mol. Sci..

[56] Sambandam T., Belousova M., Accaviti-Loper M.A., Blanquicett C., Guercello V., Raijmakers R., Nicholas A.P. (2004). Increased peptidylarginine deiminase type II in hypoxic astrocytes. Biochem. Biophys. Res. Commun..

[57] Lange S. (2016). Peptidylarginine Deiminases as Drug Targets in Neonatal Hypoxic-Ischemic Encephalopathy. Front. Neurol..

[58] Sase T., Arito M., Onodera H., Omoteyama K., Kurokawa M.S., Kagami Y., Ishigami A., Tanaka Y., Kato T. (2017). Hypoxia-induced production of peptidylarginine deiminases and citrullinated proteins in malignant glioma cells. Biochem. Biophys. Res. Commun..

[59] Yu R., Li C., Sun L., Jian L., Ma Z., Zhao J., Liu X. (2018). Hypoxia induces production of citrullinated proteins in human fibroblast-like synoviocytes through regulating HIF1α. Scand. J. Immunol..

[60] Valles J., Lago A., Santos M.T., Latorre A.M., Tembl J.I., Salom J.B., Nieves C., Moscardó A. (2017). Neutrophil extracellular traps are increased in patients with acute ischemic stroke: Prognostic,significance. Thromb. Haemost..

[61] Vaibhav K., Braun M., Alverson K., Khodadadi H., Kutiyanawalla A., Ward A., Banerjee C., Sparks T., Malik A., Rashid M.H. (2020). Neutrophil extracellular traps exacerbate neurological deficits after traumatic brain injury. Sci. Adv..

[62] He W., Zhou P., Chang Z., Liu B., Liu X., Wang Y., Li Y., Alam H.B. (2016). Inhibition of peptidylarginine deiminase attenuates inflammation and improves survival in a rat model of hemorrhagic shock. J. Surg. Res..

[63] Eghbalzadeh K., Georgi L., Louis T., Zhao H., Keser U., Weber C., Mollenhauer M., Conforti A., Wahlers T., Paunel-Görgülü A. (2019). Compromised Anti-inflammatory Action of Neutrophil Extracellular Traps in PAD4-Deficient Mice Contributes to Aggravated Acute Inflammation After Myocardial Infarction. Front. Immunol..

[64] Vincent D., Klinke M., Eschenburg G., Trochimiuk M., Appl B., Tiemann B., Bergholz R., Reinshagen K., Boettcher M. (2018). NEC is likely a NETs dependent process and markers of NETosis are predictive of NEC in mice and humans. Sci. Rep..

[65] Fan T., Zhang C., Zong M., Fan L. (2018). Hypoxia-induced autophagy is inhibited by PADI4 knockdown, which promotes apoptosis of fibroblast-like synoviocytes in rheumatoid arthritis. Mol. Med. Rep..

[66] Wang Y., Lyu Y., Tu K., Xu Q., Yang Y., Salman S., Le N., Lu H., Chen C., Zhu Y. (2021). Histone citrullination by PADI4 is required for HIF-dependent transcriptional responses to hypoxia and tumor vascularization. Sci. Adv..

[67] Bister N., Pistono C., Huremagic B., Jolkkonen J., Giugno R., Malm T. (2020). Hypoxia and extracellular vesicles: A review on methods, vesicular cargo and functions. J. Extracell. Vesicles.

[68] Yaghoubi S., Najminejad H., Dabaghian M., Karimi M.H., Abdollahpour-Alitappeh M., Rad F., Mahi-Birjand M., Mohammadi S., Mohseni F., Sobhani Lari M. (2020). How Hypoxia Regulate Exosomes in Ischemic Diseases and Cancer Microenvironment?. IUBMB Life.

[69] Venturella M., Criscuoli M., Carraro F., Naldini A., Zocco D. (2021). Interplay between Hypoxia and Extracellular Vesicles in Cancer and Inflammation. Biology.

[70] Zhang Y., Tan J., Miao Y., Zhang Q. (2021). The effect of extracellular vesicles on the regulation of mitochondria under hypoxia. Cell Death Dis..

[71] Słomka A., Urban S.K., Lukacs-Kornek V., Żekanowska E., Kornek M. (2018). Large extracellular vesicles: Have we found the holy grail of inflammation?. Front. Immunol..

[72] Taylor C.T., Colgan S.P. (2017). Regulation of Immunity and Inflammation by Hypoxia in Immunological Niches. Nat. Rev. Immunol..

[73] Eltzschig H.K., Carmeliet P. (2011). Hypoxia and Inflammation. N. Engl. J. Med..

[74] Iba T., Levy J.H., Levi M., Thachil J. (2020). Coagulopathy in COVID-19. J. Thromb. Haemost..

[75] Castro R.A., Frishman W.H. (2021). Thrombotic Complications of COVID-19 Infection: A Review. Cardiol. Rev..

[76] Vrtačnik P., Ostanek B., Mencej-Bedrač S., Marc J. (2014). The many faces of estrogen signaling. Biochem. Med..

[77] Rothenberger N.J., Somasundaram A., Stabile L.P. (2018). The Role of the Estrogen Pathway in the Tumor Microenvironment. Int. J. Mol. Sci..

[78] Potokar M., Jorgačevski J. (2021). Plectin in the Central Nervous System and a Putative Role in Brain Astrocytes. Cells.

[79] Wiche G. (2021). Plectin-Mediated Intermediate Filament Functions: Why Isoforms Matter. Cells.

[80] Kholia S., Jorfi S., Thompson P.R., Causey C.P., Nicholas A.P., Inal J.M., Lange S. (2015). A novel role for peptidylarginine deiminases in microvesicle release reveals therapeutic potential of PAD inhibition in sensitizing prostate cancer cells to chemotherapy. J. Extracell. Vesicles.

[81] Liberti M.V., Allen A.E., Ramesh V., Dai Z., Singleton K.R., Guo Z., Liu J.O., Wood K.C., Locasale J.W. (2020). Evolved resistance to partial GAPDH inhibition results in loss of the Warburg effect and in a different state of glycolysis. J. Biol. Chem..

[82] Mikeladze M.A., Dutysheva E.A., Kartsev V.G., Margulis B.A., Guzhova I.V., Lazarev V.F. (2021). Disruption of the Complex between GAPDH and Hsp70 Sensitizes C6 Glioblastoma Cells to Hypoxic Stress. Int. J. Mol. Sci..

[83] Chaput D., Kirouac L., Stevens S.M., Padmanabhan J. (2016). Potential role of PCTAIRE-2, PCTAIRE-3 and P-Histone H4 in amyloid precursor protein-dependent Alzheimer pathology. Oncotarget.

[84] Herskovits A.Z., Davies P. (2006). The regulation of tau phosphorylation by PCTAIRE 3: Implications for the pathogenesis of Alzheimer’s disease. Neurobiol. Dis..

[85] Shimizu Y., Nakai Y., Watanabe H., Iikuni S., Ono M., Saji H., Kuge Y., Saga T., Nakamoto Y. (2021). Increased [18F]FMISO accumulation under hypoxia by multidrug-resistant protein 1 inhibitors. EJNMMI Res..

[86] Lombardi M.S., van den Tweel E., Kavelaars A., Groenendaal F., van Bel F., Heijnen C.J. (2004). Hypoxia/ischemia modulates G protein-coupled receptor kinase 2 and beta-arrestin-1 levels in the neonatal rat brain. Stroke.

[87] Singh R.K., Bose D., Robertson E.S. (2021). HIF1α-Regulated Expression of the Fatty Acid Binding Protein Family Is Important for Hypoxic Reactivation of Kaposi’s Sarcoma-Associated Herpesvirus. J. Virol..

[88] McKenna Z.J., Fennel Z.J., Berkemeier Q.N., Nava R.C., Amorim F.T., Deyhle M.R., Mermier C.M. (2022). Exercise in hypobaric hypoxia increases markers of intestinal injury and symptoms of gastrointestinal distress. Exp. Physiol..

[89] Brown T.J., Kollara A., Shathasivam P., Ringuette M.J. (2019). Ventricular Zone Expressed PH Domain Containing 1 (VEPH1): An adaptor protein capable of modulating multiple signaling transduction pathways during normal and pathological development. Cell Commun. Signal..

[90] Salomon C., Ryan J., Sobrevia L., Kobayashi M., Ashman K., Mitchell M., Rice G.E. (2013). Exosomal signaling during hypoxia mediates microvascular endothelial cell migration and vasculogenesis. PLoS ONE.

[91] Méchin M.C., Takahara H., Simon M. (2020). Deimination and Peptidylarginine Deiminases in Skin Physiology and Diseases. Int. J. Mol. Sci..

[92] Zhang X., Liu X., Zhang M., Li T., Muth A., Thompson P.R., Coonrod S.A., Zhang X. (2016). Peptidylarginine deiminase 1-catalyzed histone citrullination is essential for early embryo development. Sci. Rep..

[93] Qin H., Liu X., Li F., Miao L., Li T., Xu B., An X., Muth A., Thompson P.R., Coonrod S.A. (2017). PAD1 promotes epithelial-mesenchymal transition and metastasis in triple-negative breast cancer cells by regulating MEK1-ERK1/2-MMP2 signaling. Cancer Lett..

[94] Cau L., Takahara H., Thompson P.R., Serre G., Méchin M.C., Simon M. (2019). Peptidylarginine Deiminase Inhibitor Cl-Amidine Attenuates Cornification and Interferes with the Regulation of Autophagy in Reconstructed Human Epidermis. J. Investig. Dermatol..

[95] Subramanian V., Nicholas A.P., Thompson P.R., Ferretti P. (2014). Modulation of calcium-induced cell death in human neural stem cells by the novel peptidylarginine deiminase-AIF pathway. Biochim. Biophys. Acta.

[96] Coassolo S., Davidson G., Negroni L., Gambi G., Daujat S., Romier C., Davidson I. (2021). Citrullination of pyruvate kinase M2 by PADI1 and PADI3 regulates glycolysis and cancer cell proliferation. Nat. Commun..

[97] Esposito G., Vitale A.M., Leijten F.P., Strik A.M., Koonen-Reemst A.M., Yurttas P., Robben T.J., Coonrod S., Gossen J.A. (2007). Peptidylarginine deiminase (PAD) 6 is essential for oocyte cytoskeletal sheet formation and female fertility. Mol Cell Endocrinol..

[98] Horibata S., Coonrod S.A., Cherrington B.D. (2012). Role for peptidylarginine deiminase enzymes in disease and female reproduction. J. Reprod. Dev..

[99] Xu Y., Shi Y., Fu J., Yu M., Feng R., Sang Q., Liang B., Chen B., Qu R., Li B. (2016). Mutations in PADI6 Cause Female Infertility Characterized by Early Embryonic Arrest. Am. J. Hum. Genet..

[100] Cheng Y., Si Y., Wang L., Ding M., Yu S., Lu L., Guo Y., Zong M., Fan L. (2021). The regulation of macrophage polarization by hypoxia-PADI4 coordination in Rheumatoid arthritis. Int. Immunopharmacol..

[101] Sorli S.C., van den Berghe L., Masri B., Knibiehler B., Audigier Y. (2006). Therapeutic potential of interfering with apelin signalling. Drug Discov. Today.

[102] Zhu J., Dou S., Jiang Y., Bai B., Chen J., Wang C., Cheng B. (2019). Apelin-36 exerts the cytoprotective effect against MPP+-induced cytotoxicity in SH-SY5Y cells through PI3K/Akt/mTOR autophagy pathway. Life Sci..

[103] Masoumi J., Abbasloui M., Parvan R., Mohammadnejad D., Pavon-Djavid G., Barzegari A., Abdolalizadeh J. (2018). Apelin, a promising target for Alzheimer disease prevention and treatment. Neuropeptides.

[104] Vagin O., Beenhouwer D.O. (2016). Septins: Regulators of Protein Stability. Front. Cell Dev. Biol..

[105] Xiao B., Wang S., Yang G., Sun X., Zhao S., Lin L., Cheng J., Yang W., Cong W., Sun W. (2017). HIF-1α contributes to hypoxia adaptation of the naked mole rat. Oncotarget.

[106] Kim E.B., Fang X., Fushan A.A., Huang Z., Lobanov A.V., Han L., Lobanov A.V., Han L., Marino S.M., Sun X. (2011). Genome sequencing reveals insights into physiology and longevity of the naked mole rat. Nature.

[107] King H.W., Michael M.Z., Gleadle J.M. (2012). Hypoxic Enhancement of Exosome Release by Breast Cancer Cells. BMC Cancer.

[108] Wang T., Gilkes D.M., Takano N., Xiang L., Luo W., Bishop C.J., Chaturvedi P., Green J.J., Semenza G.L. (2014). Hypoxia-Inducible Factors and RAB22A Mediate Formation of Microvesicles That Stimulate Breast Cancer Invasion and Metastasis. Proc. Natl. Acad. Sci. USA.

[109] Li L., Li C., Wang S., Wang Z., Jiang J., Wang W., Li X., Chen J., Liu K., Li C. (2016). Exosomes Derived from Hypoxic Oral Squamous Cell Carcinoma Cells Deliver MiR-21 to Normoxic Cells to Elicit a Prometastatic Phenotype. Cancer Res..

[110] Magnadóttir B., Uysal-Onganer P., Kraev I., Svansson V., Hayes P., Lange S. (2020). Deiminated proteins and extracellular vesicles—Novel serum biomarkers in whales and orca. Comp. Biochem. Physiol. Part D Genom. Proteom..

[111] Wong S.L., Wagner D.D. (2018). Peptidylarginine deiminase 4: A nuclear button triggering neutrophil extracellular traps in inflammatory diseases and aging. FASEB J..

[112] Zhang X., Bolt M., Guertin M.J., Chen W., Zhang S., Cherrington B.D., Slade D.J., Dreyton C.J., Subramanian V., Bicker K.L. (2012). Peptidylarginine deiminase 2-catalyzed histone H3 arginine 26 citrullination facilitates estrogen receptor α target gene activation. Proc. Natl. Acad. Sci. USA.

[113] Tan L., Ke Z., Tombline G., Macoretta N., Hayes K., Tian X., Lv R., Ablaeva J., Gibert M., Bhanu N.V. (2017). Naked Mole Rat Cells Have a Stable Epigenome that Resists iPSC Reprogramming. Stem Cell Rep..

[114] Théry C., Witwer K.W., Aikawa E., Alcaraz M.J., Anderson J.D., Andriantsitohaina R., Antoniou A., Arab T., Archer F., Atkin-Smith G.K. (2018). Minimal information for studies of extracellular vesicles 2018 (MISEV2018): A position statement of the International Society for Extracellular Vesicles and update of the MISEV2014 guidelines. J. Extracell. Vesicles.

[115] Nicholas A.P., Whitaker J.N. (2002). Preparation of a monoclonal antibody to citrullinated epitopes: Its characterization and some applications to immunohistochemistry in human brain. Glia.

[116] Schneider C.A., Rasband W.S., Eliceiri K.W. (2012). NIH Image to ImageJ: 25 years of image analysis. Nat. Methods.

