# Supplementary material for: Acute Hypoxia Alters Extracellular Vesicle Signatures and the Brain Citrullinome of Naked Mole-Rats (Heterocephalus glaber)
Source: Int J Mol Sci. 2022 Apr 23;23(9):4683. doi: 10.3390/ijms23094683 (PMC9100269; doi:10.3390/ijms23094683)
Supplement: Supplementary file 1 [file ijms-23-04683-s001.zip › Supp Table S1 Brain citrullinome normoxia all with specific hits highlights (1).pdf]

Supplementary Table S1. The brain citrullinome in normoxia. Full list of naked mole-rat protein hits from LC-MS/MS analysis of F95 enriched proteins isolated from brains of naked mole-rats (Heterocephalus glaber) following normoxia; hits specific for the normoxia group only are highlighted in blue.

Protein ID	Protein name
**G5ASE4**	Spectrin alpha chain, brain OS=Heterocephalus glaber OX=10181 GN=GW7_01914 PE=4 SV=1
**G5B8V8**	Spectrin beta chain OS=Heterocephalus glaber OX=10181 GN=GW7_15253 PE=3 SV=1
**G5AZQ6**	Sodium/potassium-transporting ATPase subunit alpha OS=Heterocephalus glaber OX=10181 GN=GW7_15424 PE=3 SV=1
**G5BFJ7**	Sodium/potassium-transporting ATPase subunit alpha OS=Heterocephalus glaber OX=10181 GN=GW7_09834 PE=3 SV=1
**G5BY99**	Dihydropyrimidinase-related protein 2 (Fragment) OS=Heterocephalus glaber OX=10181 GN=GW7_07625 PE=4 SV=1
**G5BL81**	ATP synthase subunit alpha OS=Heterocephalus glaber OX=10181 GN=ATP5A1 PE=3 SV=1
**G5BWK9**	Sodium/potassium-transporting ATPase subunit alpha-2 OS=Heterocephalus glaber OX=10181 GN=GW7_00530 PE=3 SV=1
**G5C895**	Clathrin heavy chain OS=Heterocephalus glaber OX=10181 GN=GW7_08300 PE=3 SV=1
**G5BB67**	ATP synthase subunit beta OS=Heterocephalus glaber OX=10181 GN=GW7_15746 PE=3 SV=1
**G5ASF4**	Dynamin-1 (Fragment) OS=Heterocephalus glaber OX=10181 GN=GW7_01924 PE=3 SV=1
**G5BHB1**	Pyruvate kinase OS=Heterocephalus glaber OX=10181 GN=GW7_06053 PE=3 SV=1
**G5BVT0**	Malic enzyme OS=Heterocephalus glaber OX=10181 GN=GW7_16261 PE=3 SV=1
**G5C9E0**	DmX-like protein 2 OS=Heterocephalus glaber OX=10181 GN=GW7_04224 PE=4 SV=1
**G5BLH0**	Syntaxin-binding protein 1 OS=Heterocephalus glaber OX=10181 GN=GW7_06738 PE=3 SV=1
**G5ATF9**	Glutamate dehydrogenase 1, mitochondrial OS=Heterocephalus glaber OX=10181 GN=GW7_14620 PE=3 SV=1
**G5AU27**	Tubulin alpha chain OS=Heterocephalus glaber OX=10181 GN=GW7_14459 PE=3 SV=1
**G5BS02**	Heat shock cognate 71 kDa protein OS=Heterocephalus glaber OX=10181 GN=GW7_03309 PE=3 SV=1
**G5BFZ1**	Tubulin beta chain OS=Heterocephalus glaber OX=10181 GN=GW7_03924 PE=3 SV=1
**G5B184**	Hexokinase-1 OS=Heterocephalus glaber OX=10181 GN=GW7_10633 PE=3 SV=1
**G5AKJ8**	Tubulin alpha chain OS=Heterocephalus glaber OX=10181 GN=GW7_07278 PE=3 SV=1
**G5B0C4**	Synapsin-1 OS=Heterocephalus glaber OX=10181 GN=GW7_10284 PE=4 SV=1
**G5C546**	Alpha-enolase OS=Heterocephalus glaber OX=10181 GN=GW7_09136 PE=2 SV=1
**G5APH2**	Tubulin beta chain OS=Heterocephalus glaber OX=10181 GN=GW7_12416 PE=3 SV=1
**G5BW26**	Non-POU domain-containing octamer-binding protein OS=Heterocephalus glaber OX=10181 GN=NONO PE=4 SV=1
**G5CAP7**	Glyceraldehyde-3-phosphate dehydrogenase (Fragment) OS=Heterocephalus glaber OX=10181 GN=GW7_12106 PE=3 SV=1
**G5BU67**	ADP/ATP translocase 2 OS=Heterocephalus glaber OX=10181 GN=GW7_07398 PE=3 SV=1
**G5BJ63**	Actin, cytoplasmic 2 OS=Heterocephalus glaber OX=10181 GN=GW7_04078 PE=3 SV=1
**G5C6D8**	Splicing factor, proline-and glutamine-rich OS=Heterocephalus glaber OX=10181 GN=GW7_05389 PE=4 SV=1
**G5ARR9**	Tenascin-R OS=Heterocephalus glaber OX=10181 GN=GW7_21557 PE=4 SV=1
**G5BPP6**	Aconitate hydratase, mitochondrial OS=Heterocephalus glaber OX=10181 GN=GW7_19434 PE=3 SV=1
**G5BI78**	Actin, cytoplasmic 1 OS=Heterocephalus glaber OX=10181 GN=GW7_16883 PE=3 SV=1
**G5ARA1**	Lamin-A/C OS=Heterocephalus glaber OX=10181 GN=GW7_14290 PE=3 SV=1
**G5BVP2**	ARF GTPase-activating protein GIT1 OS=Heterocephalus glaber OX=10181 GN=GW7_08203 PE=4 SV=1
**G5B5P2**	Serum albumin (Fragment) OS=Heterocephalus glaber OX=10181 GN=GW7_11087 PE=4 SV=1
**G5AMA4**	Heterogeneous nuclear ribonucleoprotein L OS=Heterocephalus glaber OX=10181 GN=GW7_16706 PE=4 SV=1
**G5ANG4**	Calcium-binding mitochondrial carrier protein Aralar1 OS=Heterocephalus glaber OX=10181 GN=SLC25A12 PE=3 SV=1
**G5BXS0**	Fructose-bisphosphate aldolase OS=Heterocephalus glaber OX=10181 GN=GW7_19061 PE=3 SV=1
**G5BJV3**	Alpha-1,4 glucan phosphorylase OS=Heterocephalus glaber OX=10181 GN=GW7_03733 PE=3 SV=1
**G5AST8**	Homer protein-like protein 1 (Fragment) OS=Heterocephalus glaber OX=10181 GN=GW7_06603 PE=4 SV=1
**G5BUP0**	Transketolase OS=Heterocephalus glaber OX=10181 GN=GW7_01625 PE=4 SV=1
**G5ARE5**	Vacuolar proton pump subunit B OS=Heterocephalus glaber OX=10181 GN=GW7_01073 PE=3 SV=1
**G5BKE5**	Tubulin beta chain OS=Heterocephalus glaber OX=10181 GN=GW7_11747 PE=3 SV=1

**G5BTH0**	Dihydropyrimidinase-related protein 3 OS=Heterocephalus glaber OX=10181 GN=DPYSL3 PE=4 SV=1
**G5AMM5**	LanC-like protein 2 OS=Heterocephalus glaber OX=10181 GN=GW7_07012 PE=4 SV=1
**G5BGJ6**	Contactin-1 OS=Heterocephalus glaber OX=10181 GN=GW7_19026 PE=4 SV=1
**G5AKA3**	L-lactate dehydrogenase OS=Heterocephalus glaber OX=10181 GN=GW7_11628 PE=2 SV=1
**G5B8Q7**	AP-2 complex subunit alpha OS=Heterocephalus glaber OX=10181 GN=GW7_15304 PE=3 SV=1
**G5BD04**	Heat shock-related 70 kDa protein 2 OS=Heterocephalus glaber OX=10181 GN=GW7_17513 PE=3 SV=1
**G5BKE6**	Tubulin beta chain OS=Heterocephalus glaber OX=10181 GN=GW7_11748 PE=3 SV=1
**G5BNN8**	Heat shock cognate protein HSP 90-beta OS=Heterocephalus glaber OX=10181 GN=GW7_19161 PE=3 SV=1
**G5BTG2**	Putative ATP-dependent RNA helicase DHX30 (Fragment) OS=Heterocephalus glaber OX=10181 GN=GW7_17258 PE=4 SV=1
**G5BXB9**	Transitional endoplasmic reticulum ATPase (Fragment) OS=Heterocephalus glaber OX=10181 GN=GW7_03434 PE=3 SV=1
**G5BXH3**	Putative proline--tRNA ligase, mitochondrial OS=Heterocephalus glaber OX=10181 GN=PARS2 PE=4 SV=1
**G5B139**	Amino acid transporter OS=Heterocephalus glaber OX=10181 GN=GW7_01386 PE=3 SV=1
**G5ATH6**	Guanine nucleotide-binding protein G(I)/G(S)/G(T) subunit beta-1 OS=Heterocephalus glaber OX=10181 GN=GNB1 PE=4 SV=1
**G5AXZ3**	2-oxoglutarate dehydrogenase E1 component-like, mitochondrial OS=Heterocephalus glaber OX=10181 GN=GW7_10398 PE=4 SV=1
**G5BM52**	Creatine kinase U-type, mitochondrial OS=Heterocephalus glaber OX=10181 GN=GW7_18774 PE=3 SV=1
**G5B750**	AP complex subunit beta OS=Heterocephalus glaber OX=10181 GN=GW7_18272 PE=3 SV=1
**G5B2W9**	ADP/ATP translocase 1 OS=Heterocephalus glaber OX=10181 GN=GW7_12487 PE=3 SV=1
**G5B318**	Dihydropyrimidinase-related protein 1 (Fragment) OS=Heterocephalus glaber OX=10181 GN=GW7_14551 PE=4 SV=1
**G5BSI9**	Calcium-transporting ATPase OS=Heterocephalus glaber OX=10181 GN=GW7_13643 PE=3 SV=1
**G5BS33**	Hemoglobin subunit beta OS=Heterocephalus glaber OX=10181 GN=GW7_03824 PE=3 SV=1
**G5BMY6**	Vesicle-fusing ATPase (Fragment) OS=Heterocephalus glaber OX=10181 GN=GW7_07669 PE=4 SV=1
**G5C4F1**	Tubulin beta chain OS=Heterocephalus glaber OX=10181 GN=GW7_15271 PE=3 SV=1
**G5AL32**	V-type proton ATPase catalytic subunit A OS=Heterocephalus glaber OX=10181 GN=GW7_03605 PE=3 SV=1
**G5APJ0**	Synapsin-2 OS=Heterocephalus glaber OX=10181 GN=GW7_10966 PE=4 SV=1
**G5ASD2**	Leucine-rich repeat-containing protein 8A OS=Heterocephalus glaber OX=10181 GN=LRRC8A PE=4 SV=1
**G5C9J0**	Synaptotagmin-1 OS=Heterocephalus glaber OX=10181 GN=GW7_11734 PE=4 SV=1
**G5BE32**	Putative pre-mRNA-splicing factor ATP-dependent RNA helicase DHX15 OS=Heterocephalus glaber OX=10181 GN=DHX15 PE=4 SV=1
**G5BV89**	Cytochrome b-c1 complex subunit 2, mitochondrial OS=Heterocephalus glaber OX=10181 GN=UQCRC2 PE=3 SV=1
**G5C3C3**	Tubulin beta chain OS=Heterocephalus glaber OX=10181 GN=GW7_04753 PE=3 SV=1
**G5AZ41**	Rho guanine nucleotide exchange factor 7 OS=Heterocephalus glaber OX=10181 GN=GW7_00283 PE=4 SV=1
**G5B212**	Microtubule-associated protein 1B OS=Heterocephalus glaber OX=10181 GN=GW7_19015 PE=4 SV=1
**G5C6E3**	Neurochondrin (Fragment) OS=Heterocephalus glaber OX=10181 GN=GW7_05394 PE=4 SV=1
**G5BW99**	Mitochondrial 2-oxoglutarate/malate carrier protein isoform 2 OS=Heterocephalus glaber OX=10181 GN=SLC25A11 PE=3 SV=1
**G5B1L2**	Band 4.1-like protein 3 OS=Heterocephalus glaber OX=10181 GN=GW7_17835 PE=4 SV=1
**G5BBZ6**	Guanine nucleotide-binding protein G(I)/G(S)/G(T) subunit beta-2 OS=Heterocephalus glaber OX=10181 GN=GNB2 PE=4 SV=1
**G5ALS1**	Keratin, type II cytoskeletal 6B OS=Heterocephalus glaber OX=10181 GN=GW7_03778 PE=3 SV=1
**G5B3C5**	Serine/threonine-protein phosphatase 2A 65 kDa regulatory subunit A alpha isoform OS=Heterocephalus glaber OX=10181 GN=GW7_06227 PE=4 SV=1
**G5C5M0**	NADH-ubiquinone oxidoreductase 75 kDa subunit, mitochondrial OS=Heterocephalus glaber OX=10181 GN=GW7_17583 PE=3 SV=1
**G5AVJ8**	Nck-associated protein 1 (Fragment) OS=Heterocephalus glaber OX=10181 GN=GW7_12399 PE=4 SV=1
**G5AMR7**	Protein ERGIC-53 OS=Heterocephalus glaber OX=10181 GN=GW7_20044 PE=4 SV=1
**G5BXY1**	Hemoglobin subunit alpha OS=Heterocephalus glaber OX=10181 GN=HBA2 PE=3 SV=1
**G5BEG2**	L-lactate dehydrogenase OS=Heterocephalus glaber OX=10181 GN=GW7_05659 PE=2 SV=1
**G5BAX8**	Septin-7 OS=Heterocephalus glaber OX=10181 GN=GW7_00168 PE=3 SV=1
**G5BVH5**	Fructose-bisphosphate aldolase OS=Heterocephalus glaber OX=10181 GN=GW7_08177 PE=3 SV=1
**G5BYJ8**	Hemoglobin subunit beta OS=Heterocephalus glaber OX=10181 GN=GW7_14163 PE=3 SV=1
**G5B0L2**	2',3'-cyclic-nucleotide 3'-phosphodiesterase (Fragment) OS=Heterocephalus glaber OX=10181 GN=GW7_06865 PE=4 SV=1
**G5C8F7**	AP-2 complex subunit alpha OS=Heterocephalus glaber OX=10181 GN=GW7_12697 PE=3 SV=1

**G5B6V7**	78 kDa glucose-regulated protein OS=Heterocephalus glaber OX=10181 GN=HSPA5 PE=3 SV=1
**G5C4U7**	Aspartate aminotransferase OS=Heterocephalus glaber OX=10181 GN=GOT1 PE=4 SV=1
**G5B0M6**	Keratin, type I cytoskeletal 14 OS=Heterocephalus glaber OX=10181 GN=GW7_06879 PE=3 SV=1
**G5AZ30**	Endoplasmic reticulum resident protein 44 OS=Heterocephalus glaber OX=10181 GN=ERP44 PE=4 SV=1
**G5C1Q0**	Splicing factor 3B subunit 3 OS=Heterocephalus glaber OX=10181 GN=GW7_01482 PE=4 SV=1
**G5BMD6**	Ankyrin-2 (Fragment) OS=Heterocephalus glaber OX=10181 GN=GW7_01307 PE=4 SV=1
**G5ATM3**	ERC protein 2 OS=Heterocephalus glaber OX=10181 GN=GW7_00199 PE=4 SV=1
**G5BWZ4**	Spectrin beta chain OS=Heterocephalus glaber OX=10181 GN=GW7_07212 PE=3 SV=1
**G5BG62**	Malate dehydrogenase OS=Heterocephalus glaber OX=10181 GN=GW7_08880 PE=3 SV=1
**G5B4D4**	Ras-related protein Rab-3A OS=Heterocephalus glaber OX=10181 GN=RAB3A PE=4 SV=1
**G5AZF9**	Ubiquitin-like modifier-activating enzyme 1 OS=Heterocephalus glaber OX=10181 GN=GW7_11282 PE=3 SV=1
**G5BMR4**	Guanine nucleotide-binding protein G(O) subunit alpha (Fragment) OS=Heterocephalus glaber OX=10181 GN=GW7_09344 PE=4 SV=1
**G5C7R8**	Constitutive coactivator of peroxisome proliferator-activated receptor gamma OS=Heterocephalus glaber OX=10181 GN=GW7_11305 PE=4 SV=1
**G5C3R7**	Septin-2 OS=Heterocephalus glaber OX=10181 GN=GW7_11070 PE=3 SV=1
**G5AKB7**	Anion exchange protein OS=Heterocephalus glaber OX=10181 GN=GW7_07691 PE=3 SV=1
**G5C1L8**	Dihydrolipoyllysine-residue succinyltransferase component of 2-oxoglutarate dehydrogenase complex, mitochondrial OS=Heterocephalus glaber OX=10181 GN=DLST PE=4 SV=1
**G5AXH0**	Actin, gamma-enteric smooth muscle OS=Heterocephalus glaber OX=10181 GN=GW7_02888 PE=3 SV=1
**G5B577**	Acylglycerol kinase, mitochondrial OS=Heterocephalus glaber OX=10181 GN=GW7_11853 PE=4 SV=1
**G5AP01**	14-3-3 protein zeta/delta OS=Heterocephalus glaber OX=10181 GN=GW7_00766 PE=3 SV=1
**G5CAA4**	Isocitrate dehydrogenase [NADP] OS=Heterocephalus glaber OX=10181 GN=GW7_08829 PE=3 SV=1
**G5BGB3**	Cullin-associated NEDD8-dissociated protein 1 OS=Heterocephalus glaber OX=10181 GN=CAND1 PE=4 SV=1
**G5B360**	Calcium-transporting ATPase OS=Heterocephalus glaber OX=10181 GN=GW7_12839 PE=3 SV=1
**G5BWB9**	ATP-dependent 6-phosphofructokinase OS=Heterocephalus glaber OX=10181 GN=GW7_07004 PE=3 SV=1
**G5B6D0**	Glutamine synthetase OS=Heterocephalus glaber OX=10181 GN=GW7_08254 PE=3 SV=1
**G5B2A3**	Protein kinase C-binding protein NELL2 OS=Heterocephalus glaber OX=10181 GN=GW7_03965 PE=4 SV=1
**G5B7T4**	Kinesin heavy chain isoform 5C OS=Heterocephalus glaber OX=10181 GN=GW7_17996 PE=3 SV=1
**G5BTE1**	AP-2 complex subunit mu-1 OS=Heterocephalus glaber OX=10181 GN=GW7_17237 PE=3 SV=1
**G5BLU3**	DnaJ-like protein subfamily C member 10 (Fragment) OS=Heterocephalus glaber OX=10181 GN=GW7_10932 PE=4 SV=1
**G5C394**	Calcium/calmodulin-dependent protein kinase type II alpha chain OS=Heterocephalus glaber OX=10181 GN=GW7_00118 PE=4 SV=1
**G5C8G6**	Mitochondrial glutamate carrier 1 OS=Heterocephalus glaber OX=10181 GN=GW7_12706 PE=3 SV=1
**G5BU71**	Septin-6 OS=Heterocephalus glaber OX=10181 GN=GW7_07402 PE=3 SV=1
**G5C9G0**	Amino acid transporter OS=Heterocephalus glaber OX=10181 GN=GW7_08002 PE=3 SV=1
**G5ALS3**	Keratin, type II cytoskeletal 5 OS=Heterocephalus glaber OX=10181 GN=GW7_03780 PE=3 SV=1
**G5BZS4**	Actin, alpha cardiac muscle 1 OS=Heterocephalus glaber OX=10181 GN=GW7_14263 PE=3 SV=1
**G5C0R6**	Dipeptidyl peptidase 9 OS=Heterocephalus glaber OX=10181 GN=GW7_20503 PE=3 SV=1
**G5BUK7**	Pyruvate dehydrogenase E1 component subunit beta OS=Heterocephalus glaber OX=10181 GN=PDHB PE=4 SV=1
**G5BLD9**	Myelin proteolipid protein (Fragment) OS=Heterocephalus glaber OX=10181 GN=GW7_06253 PE=4 SV=1
**G5B2K8**	Phosphate carrier protein, mitochondrial OS=Heterocephalus glaber OX=10181 GN=GW7_08592 PE=3 SV=1
**G5BHP2**	E3 ubiquitin-protein ligase CBL (Fragment) OS=Heterocephalus glaber OX=10181 GN=GW7_18686 PE=4 SV=1
**G5B251**	Heat shock cognate 71 kDa protein OS=Heterocephalus glaber OX=10181 GN=GW7_09579 PE=3 SV=1
**G5B6P0**	Alpha-1,4 glucan phosphorylase OS=Heterocephalus glaber OX=10181 GN=GW7_20662 PE=3 SV=1
**G5BI79**	Fascin OS=Heterocephalus glaber OX=10181 GN=GW7_16884 PE=4 SV=1
**G5C2C4**	Neuronal cell adhesion molecule (Fragment) OS=Heterocephalus glaber OX=10181 GN=GW7_15852 PE=4 SV=1
**G5C7M9**	Dynamin-3 (Fragment) OS=Heterocephalus glaber OX=10181 GN=GW7_06265 PE=3 SV=1
**G5AVL7**	Cytochrome b-c1 complex subunit 1, mitochondrial OS=Heterocephalus glaber OX=10181 GN=UQCRC1 PE=4 SV=1
**G5BH24**	Synaptic vesicle glycoprotein 2A OS=Heterocephalus glaber OX=10181 GN=SV2A PE=4 SV=1
**G5BY30**	Aminopeptidase OS=Heterocephalus glaber OX=10181 GN=GW7_00454 PE=3 SV=1
**G5B6T3**	ATP-dependent 6-phosphofructokinase OS=Heterocephalus glaber OX=10181 GN=GW7_21506 PE=3 SV=1
**G5BE96**	Synaptojanin-1 (Fragment) OS=Heterocephalus glaber OX=10181 GN=GW7_08747 PE=4 SV=1

**G5BKW4**	Paraspeckle component 1 OS=Heterocephalus glaber OX=10181 GN=GW7_03914 PE=4 SV=1
**G5C5T9**	Cytochrome c1, heme protein, mitochondrial OS=Heterocephalus glaber OX=10181 GN=GW7_19247 PE=4 SV=1
**G5BA48**	Stress-70 protein, mitochondrial OS=Heterocephalus glaber OX=10181 GN=GW7_10091 PE=3 SV=1
**G5AXS2**	Ras-related protein Rab-14 OS=Heterocephalus glaber OX=10181 GN=RAB14 PE=4 SV=1
**G5AMB0**	Alpha-actinin-4 OS=Heterocephalus glaber OX=10181 GN=GW7_16712 PE=4 SV=1
**G5BKG1**	Mitogen-activated protein kinase OS=Heterocephalus glaber OX=10181 GN=GW7_15351 PE=4 SV=1
**G5AKU7**	Aldehyde dehydrogenase, mitochondrial isoform 1 OS=Heterocephalus glaber OX=10181 GN=ALDH2 PE=3 SV=1
**G5CAF3**	Sideroflexin-3 OS=Heterocephalus glaber OX=10181 GN=GW7_15882 PE=4 SV=1
**G5B840**	Band 4.1-like protein 1 OS=Heterocephalus glaber OX=10181 GN=GW7_21265 PE=4 SV=1
**G5C7U4**	T-complex protein 1 subunit beta OS=Heterocephalus glaber OX=10181 GN=GW7_11343 PE=3 SV=1
**G5BT87**	Histidine-rich glycoprotein OS=Heterocephalus glaber OX=10181 GN=GW7_17202 PE=4 SV=1
**G5C8M3**	Malate dehydrogenase OS=Heterocephalus glaber OX=10181 GN=GW7_04884 PE=3 SV=1
**G5BLR5**	Fructose-bisphosphate aldolase A OS=Heterocephalus glaber OX=10181 GN=GW7_10395 PE=4 SV=1
**G5CAZ8**	Acetyltransferase component of pyruvate dehydrogenase complex OS=Heterocephalus glaber OX=10181 GN=DLAT PE=3 SV=1
**G5CB51**	Sodium/potassium-transporting ATPase subunit beta OS=Heterocephalus glaber OX=10181 GN=GW7_09541 PE=3 SV=1
**G5C530**	6-phosphogluconate dehydrogenase, decarboxylating OS=Heterocephalus glaber OX=10181 GN=GW7_09120 PE=3 SV=1
**G5B0M4**	Keratin, type I cytoskeletal 17 OS=Heterocephalus glaber OX=10181 GN=GW7_06877 PE=3 SV=1
**G5BN06**	Heat shock 70 kDa protein 4 OS=Heterocephalus glaber OX=10181 GN=HSPA4 PE=3 SV=1
**G5CAR8**	Gamma-enolase OS=Heterocephalus glaber OX=10181 GN=GW7_12127 PE=2 SV=1
**G5BUH8**	Dynamin-2 OS=Heterocephalus glaber OX=10181 GN=GW7_13280 PE=3 SV=1
**G5BJQ1**	Septin-11 OS=Heterocephalus glaber OX=10181 GN=GW7_19874 PE=3 SV=1
**G5BY58**	NADH dehydrogenase (Ubiquinone) 1 beta subcomplex subunit 10 OS=Heterocephalus glaber OX=10181 GN=NDUFB10 PE=4 SV=1
**G5BX71**	Calcium-transporting ATPase OS=Heterocephalus glaber OX=10181 GN=GW7_19471 PE=3 SV=1
**G5ASS3**	T-complex protein 1 subunit zeta isoform a OS=Heterocephalus glaber OX=10181 GN=CCT6A PE=3 SV=1
**G5B4U5**	Uncharacterized protein OS=Heterocephalus glaber OX=10181 GN=GW7_03212 PE=4 SV=1
**G5C7W9**	Alpha-actinin-1 OS=Heterocephalus glaber OX=10181 GN=GW7_03701 PE=4 SV=1
**G5BFK9**	Rab GDP dissociation inhibitor OS=Heterocephalus glaber OX=10181 GN=GW7_19328 PE=3 SV=1
**G5B5F3**	Vesicle-associated membrane protein 2 (Fragment) OS=Heterocephalus glaber OX=10181 GN=GW7_13855 PE=4 SV=1
**G5BVL3**	14-3-3 protein epsilon OS=Heterocephalus glaber OX=10181 GN=GW7_08232 PE=3 SV=1
**G5CAX4**	Heat shock cognate 71 kDa protein OS=Heterocephalus glaber OX=10181 GN=GW7_21369 PE=3 SV=1
**G5AKS9**	Nitric oxide synthase, brain OS=Heterocephalus glaber OX=10181 GN=GW7_10175 PE=4 SV=1
**G5BN09**	Voltage-dependent anion-selective channel protein 1 OS=Heterocephalus glaber OX=10181 GN=VDAC1 PE=4 SV=1
**G5AP69**	Serine/threonine-protein phosphatase (Fragment) OS=Heterocephalus glaber OX=10181 GN=GW7_10360 PE=3 SV=1
**G5BWX1**	Ras-related protein Rab-1B OS=Heterocephalus glaber OX=10181 GN=RAB1B PE=4 SV=1
**G5CAS2**	Prohibitin-2 OS=Heterocephalus glaber OX=10181 GN=GW7_12131 PE=4 SV=1
**G5BTW5**	NADH dehydrogenase [ubiquinone] flavoprotein 1, mitochondrial OS=Heterocephalus glaber OX=10181 GN=NDUFV1 PE=3 SV=1
**G5ARM9**	Heterogeneous nuclear ribonucleoprotein K OS=Heterocephalus glaber OX=10181 GN=HNRNPK PE=4 SV=1
**G5BHY3**	PITH domain-containing protein (Fragment) OS=Heterocephalus glaber OX=10181 GN=GW7_07493 PE=4 SV=1
**G5B230**	Intercellular adhesion molecule 5 OS=Heterocephalus glaber OX=10181 GN=GW7_08246 PE=4 SV=1
**G5C1Y4**	T-complex protein 1 subunit delta OS=Heterocephalus glaber OX=10181 GN=GW7_01322 PE=3 SV=1
**G5B2V7**	60 kDa heat shock protein, mitochondrial OS=Heterocephalus glaber OX=10181 GN=GW7_17339 PE=4 SV=1
**G5C773**	Band 4.1-like protein 2 OS=Heterocephalus glaber OX=10181 GN=GW7_21858 PE=4 SV=1
**G5ARZ9**	NADH dehydrogenase (Ubiquinone) 1 alpha subcomplex subunit 9, mitochondrial OS=Heterocephalus glaber OX=10181 GN=NDUFA9 PE=4 SV=1
**G5AKJ4**	Aspartyl aminopeptidase OS=Heterocephalus glaber OX=10181 GN=DNPEP PE=3 SV=1
**G5BSN0**	Dedicator of cytokinesis protein 8 OS=Heterocephalus glaber OX=10181 GN=GW7_01505 PE=3 SV=1
**G5BSI1**	Dynamin-1-like protein OS=Heterocephalus glaber OX=10181 GN=GW7_21637 PE=3 SV=1
**G5AU37**	ADP-ribosylation factor 3 OS=Heterocephalus glaber OX=10181 GN=GW7_14469 PE=3 SV=1

**G5C0K7**	Myelin basic protein OS=Heterocephalus glaber OX=10181 GN=GW7_03536 PE=4 SV=1
**G5AW57**	Aspartate aminotransferase OS=Heterocephalus glaber OX=10181 GN=GOT2 PE=4 SV=1
**G5AZH3**	Cytoplasmic FMR1-interacting protein 1 (Fragment) OS=Heterocephalus glaber OX=10181 GN=GW7_18826 PE=4 SV=1
**G5ALK7**	Elongation factor 1-alpha OS=Heterocephalus glaber OX=10181 GN=GW7_12808 PE=3 SV=1
**G5BAY8**	Lysine-specific demethylase 5C OS=Heterocephalus glaber OX=10181 GN=GW7_04947 PE=4 SV=1
**G5AKF3**	Myelin-oligodendrocyte glycoprotein (Fragment) OS=Heterocephalus glaber OX=10181 GN=GW7_10279 PE=4 SV=1
**G5BF93**	Trimethyllysine dioxygenase, mitochondrial OS=Heterocephalus glaber OX=10181 GN=GW7_10776 PE=4 SV=1
**G5B3J1**	Rab GDP dissociation inhibitor (Fragment) OS=Heterocephalus glaber OX=10181 GN=GW7_02321 PE=3 SV=1
**G5C8D3**	Elongation factor 1-alpha (Fragment) OS=Heterocephalus glaber OX=10181 GN=GW7_09733 PE=3 SV=1
**G5BEX6**	Neural cell adhesion molecule 1 OS=Heterocephalus glaber OX=10181 GN=GW7_02493 PE=4 SV=1
**G5BQX0**	AMP deaminase OS=Heterocephalus glaber OX=10181 GN=GW7_20134 PE=3 SV=1
**G5C5Q2**	ELKS/RAB6-interacting/CAST family member 1 OS=Heterocephalus glaber OX=10181 GN=GW7_19210 PE=4 SV=1
**G5AZ97**	Triple functional domain protein OS=Heterocephalus glaber OX=10181 GN=GW7_01985 PE=4 SV=1
**G5AXV6**	ATP synthase subunit gamma OS=Heterocephalus glaber OX=10181 GN=GW7_14036 PE=3 SV=1
**G5ALD6**	D-beta-hydroxybutyrate dehydrogenase, mitochondrial OS=Heterocephalus glaber OX=10181 GN=BDH1 PE=3 SV=1
**G5AQD6**	3,2-trans-enoyl-CoA isomerase, mitochondrial OS=Heterocephalus glaber OX=10181 GN=GW7_05021 PE=3 SV=1
**G5APX0**	AP complex subunit beta OS=Heterocephalus glaber OX=10181 GN=GW7_05313 PE=3 SV=1
**G5BQX2**	Glutathione S-transferase OS=Heterocephalus glaber OX=10181 GN=GW7_20136 PE=3 SV=1
**G5BKX2**	Cytoplasmic FMR1-interacting protein 2 OS=Heterocephalus glaber OX=10181 GN=GW7_12158 PE=4 SV=1
**G5B6W0**	Protein SCAI OS=Heterocephalus glaber OX=10181 GN=GW7_01139 PE=4 SV=1
**G5BG59**	14-3-3 protein gamma (Fragment) OS=Heterocephalus glaber OX=10181 GN=GW7_08877 PE=3 SV=1
**G5CBK9**	Ras-related protein Rab-10 OS=Heterocephalus glaber OX=10181 GN=GW7_17041 PE=4 SV=1
**G5CA17**	Septin-5 (Fragment) OS=Heterocephalus glaber OX=10181 GN=GW7_11135 PE=3 SV=1
**G5B928**	Calcium-transporting ATPase OS=Heterocephalus glaber OX=10181 GN=GW7_14600 PE=3 SV=1
**G5B9Q5**	Zinc transporter ZIP14 (Fragment) OS=Heterocephalus glaber OX=10181 GN=GW7_20731 PE=4 SV=1
**G5BBV6**	14-3-3 protein eta OS=Heterocephalus glaber OX=10181 GN=GW7_17673 PE=3 SV=1
**G5BXQ6**	Neurofascin OS=Heterocephalus glaber OX=10181 GN=GW7_15814 PE=4 SV=1
**G5C4P1**	Prohibitin OS=Heterocephalus glaber OX=10181 GN=GW7_21759 PE=4 SV=1
**G5BCQ6**	Transcriptional activator protein Pur-alpha OS=Heterocephalus glaber OX=10181 GN=GW7_08872 PE=4 SV=1
**G5BXR0**	Acetyl-CoA acetyltransferase, mitochondrial OS=Heterocephalus glaber OX=10181 GN=GW7_19747 PE=3 SV=1
**G5AMP1**	ADP/ATP translocase 3 OS=Heterocephalus glaber OX=10181 GN=GW7_12495 PE=3 SV=1
**G5ALU1**	Regulating synaptic membrane exocytosis protein 1 OS=Heterocephalus glaber OX=10181 GN=GW7_17598 PE=4 SV=1
**G5BBP4**	EF-hand calcium-binding protein 1 OS=Heterocephalus glaber OX=10181 GN=GW7_09364 PE=4 SV=1
**G5C2C6**	Dihydrolipoyl dehydrogenase OS=Heterocephalus glaber OX=10181 GN=DLD PE=3 SV=1
**G5B0J8**	V-type proton ATPase subunit a OS=Heterocephalus glaber OX=10181 GN=GW7_06851 PE=3 SV=1
**G5ALX2**	Catenin beta-1 OS=Heterocephalus glaber OX=10181 GN=GW7_09171 PE=4 SV=1
**G5BJ39**	Keratin, type II cytoskeletal 8 OS=Heterocephalus glaber OX=10181 GN=GW7_10816 PE=2 SV=1
**G5AWZ4**	Coronin OS=Heterocephalus glaber OX=10181 GN=GW7_02143 PE=3 SV=1
**G5C9N9**	Myotubularin (Fragment) OS=Heterocephalus glaber OX=10181 GN=GW7_05283 PE=4 SV=1
**G5BI06**	Basement membrane-specific heparan sulfate proteoglycan core protein OS=Heterocephalus glaber OX=10181 GN=GW7_07516 PE=4 SV=1
**G5BDV3**	Rap1 GTPase-GDP dissociation stimulator 1 (Fragment) OS=Heterocephalus glaber OX=10181 GN=GW7_10113 PE=4 SV=1
**G5AM26**	ATP synthase subunit O, mitochondrial OS=Heterocephalus glaber OX=10181 GN=GW7_16287 PE=3 SV=1
**G5BZ40**	Pyruvate dehydrogenase E1 component subunit alpha OS=Heterocephalus glaber OX=10181 GN=GW7_01970 PE=4 SV=1
**G5BN93**	Synaptic vesicle membrane protein VAT-1-like protein OS=Heterocephalus glaber OX=10181 GN=GW7_07847 PE=4 SV=1
**G5CAR5**	Triosephosphate isomerase OS=Heterocephalus glaber OX=10181 GN=GW7_12124 PE=3 SV=1

**G5BZY6**	Guanine nucleotide-binding protein G(I), alpha-2 subunit OS=Heterocephalus glaber OX=10181 GN=GW7_19941 PE=4 SV=1
**G5C3N6**	Vacuolar protein sorting-associated protein 35 OS=Heterocephalus glaber OX=10181 GN=GW7_08700 PE=3 SV=1
**G5AKP8**	Phosphatidylinositol-4-phosphate 5-kinase type-1 gamma (Fragment) OS=Heterocephalus glaber OX=10181 GN=GW7_11309 PE=4 SV=1
**G5B2P2**	Heat shock protein 105 kDa OS=Heterocephalus glaber OX=10181 GN=GW7_02038 PE=3 SV=1
**G5B3I7**	Ras-related protein Rab-5A OS=Heterocephalus glaber OX=10181 GN=RAB5A PE=4 SV=1
**G5BIL1**	2-oxoglutarate dehydrogenase E1 component, mitochondrial OS=Heterocephalus glaber OX=10181 GN=GW7_08272 PE=4 SV=1
**G5AZR4**	Platelet-activating factor acetylhydrolase IB subunit gamma OS=Heterocephalus glaber OX=10181 GN=PAFAH1B3 PE=4 SV=1
**G5B2Y7**	WD repeat-containing protein 1 OS=Heterocephalus glaber OX=10181 GN=GW7_14537 PE=4 SV=1
**G5AKY4**	Heat shock 70 kDa protein 4L OS=Heterocephalus glaber OX=10181 GN=GW7_10899 PE=3 SV=1
**G5C8M4**	UTP--glucose-1-phosphate uridylyltransferase OS=Heterocephalus glaber OX=10181 GN=GW7_04885 PE=3 SV=1
**G5C7J1**	Voltage-dependent anion-selective channel protein 2 OS=Heterocephalus glaber OX=10181 GN=GW7_13179 PE=4 SV=1
**G5BSD0**	4-aminobutyrate aminotransferase, mitochondrial (Fragment) OS=Heterocephalus glaber OX=10181 GN=GW7_08976 PE=3 SV=1
**G5BYD1**	Syntaxin-1B OS=Heterocephalus glaber OX=10181 GN=GW7_05131 PE=3 SV=1
**G5B1C5**	T-complex protein 1 subunit theta OS=Heterocephalus glaber OX=10181 GN=GW7_13214 PE=3 SV=1
**G5BSN2**	Phosphoglucomutase-1 OS=Heterocephalus glaber OX=10181 GN=GW7_01507 PE=4 SV=1
**G5AR92**	T-complex protein 1 subunit gamma (Fragment) OS=Heterocephalus glaber OX=10181 GN=GW7_14281 PE=3 SV=1
**G5CBF1**	Protein kinase C gamma type (Fragment) OS=Heterocephalus glaber OX=10181 GN=GW7_20162 PE=3 SV=1
**G5BDP5**	Septin-8 OS=Heterocephalus glaber OX=10181 GN=GW7_11423 PE=3 SV=1
**G5BCZ6**	Ras-related protein Rab-2A OS=Heterocephalus glaber OX=10181 GN=GW7_17505 PE=4 SV=1
**G5B2F5**	Actin, cytoplasmic 1 OS=Heterocephalus glaber OX=10181 GN=GW7_00567 PE=3 SV=1
**G5BA18**	CB1 cannabinoid receptor-interacting protein 1 OS=Heterocephalus glaber OX=10181 GN=GW7_02210 PE=4 SV=1
**G5C2D5**	cAMP-dependent protein kinase type II-beta regulatory subunit OS=Heterocephalus glaber OX=10181 GN=PRKAR2B PE=4 SV=1
**G5C4L3**	Nucleoside diphosphate kinase (Fragment) OS=Heterocephalus glaber OX=10181 GN=GW7_21798 PE=3 SV=1
**G5BAB4**	Constitutive coactivator of PPAR-gamma-like protein 2 OS=Heterocephalus glaber OX=10181 GN=GW7_01037 PE=4 SV=1
**G5ASQ2**	Septin-8 OS=Heterocephalus glaber OX=10181 GN=GW7_12246 PE=3 SV=1
**G5BRK8**	Importin-7 OS=Heterocephalus glaber OX=10181 GN=GW7_21205 PE=4 SV=1
**G5ANV4**	Golgin subfamily A member 3 OS=Heterocephalus glaber OX=10181 GN=GW7_09569 PE=4 SV=1
**G5AM45**	Zinc transporter 1 OS=Heterocephalus glaber OX=10181 GN=GW7_16306 PE=4 SV=1
**G5BJY3**	Succinate-semialdehyde dehydrogenase (Fragment) OS=Heterocephalus glaber OX=10181 GN=GW7_06350 PE=3 SV=1
**G5CAI7**	Cytosolic purine 5'-nucleotidase OS=Heterocephalus glaber OX=10181 GN=GW7_15916 PE=4 SV=1
**G5ASH7**	Guanine deaminase OS=Heterocephalus glaber OX=10181 GN=GW7_07953 PE=3 SV=1
**G5CAH6**	Alpha-centractin OS=Heterocephalus glaber OX=10181 GN=ACTR1A PE=3 SV=1
**G5ALN8**	Heterogeneous nuclear ribonucleoprotein D0 (Fragment) OS=Heterocephalus glaber OX=10181 GN=GW7_10606 PE=4 SV=1
**G5BAI1**	IQ motif and SEC7 domain-containing protein 1 OS=Heterocephalus glaber OX=10181 GN=GW7_02159 PE=4 SV=1
**G5BB78**	Citrate synthase (Fragment) OS=Heterocephalus glaber OX=10181 GN=GW7_15757 PE=3 SV=1
**G5BJK0**	Synaptophysin OS=Heterocephalus glaber OX=10181 GN=GW7_20097 PE=4 SV=1
**G5BS94**	Glutathione S-transferase P OS=Heterocephalus glaber OX=10181 GN=GW7_14677 PE=3 SV=1
**G5BZB8**	Elongation factor 2 OS=Heterocephalus glaber OX=10181 GN=EEF2 PE=4 SV=1
**G5CBQ4**	T-complex protein 1 subunit eta OS=Heterocephalus glaber OX=10181 GN=GW7_03296 PE=3 SV=1
**G5BRD7**	Sarcoplasmic/endoplasmic reticulum calcium ATPase 1 OS=Heterocephalus glaber OX=10181 GN=GW7_14894 PE=3 SV=1
**G5BFB4**	Dihydropyrimidinase-related protein 4 OS=Heterocephalus glaber OX=10181 GN=GW7_14070 PE=4 SV=1
**G5C9Y1**	Phospholipase D3 OS=Heterocephalus glaber OX=10181 GN=GW7_03571 PE=4 SV=1

**G5BCG5**	Metabotropic glutamate receptor 4 OS=Heterocephalus glaber OX=10181 GN=GW7_15655 PE=3 SV=1
**G5B710**	T-complex protein 1 subunit epsilon OS=Heterocephalus glaber OX=10181 GN=GW7_03792 PE=3 SV=1
**G5BPM1**	Alpha-2-macroglobulin OS=Heterocephalus glaber OX=10181 GN=GW7_17301 PE=4 SV=1
**G5ATL5**	Phosphoinositide phospholipase C OS=Heterocephalus glaber OX=10181 GN=GW7_07363 PE=4 SV=1
**G5B615**	V-type proton ATPase subunit H OS=Heterocephalus glaber OX=10181 GN=ATP6V1H PE=3 SV=1
**G5BWI0**	NADH dehydrogenase (Ubiquinone) iron-sulfur protein 2, mitochondrial isoform 1 OS=Heterocephalus glaber OX=10181 GN=NDUFS2 PE=3 SV=1
**G5BQX5**	Glutathione S-transferase OS=Heterocephalus glaber OX=10181 GN=GW7_20139 PE=3 SV=1
**G5BCH1**	Alpha-adducin OS=Heterocephalus glaber OX=10181 GN=ADD1 PE=4 SV=1
**G5C4J2**	Glyceraldehyde-3-phosphate dehydrogenase OS=Heterocephalus glaber OX=10181 GN=GW7_21777 PE=4 SV=1
**G5BPB2**	NADH dehydrogenase [ubiquinone] iron-sulfur protein 3, mitochondrial OS=Heterocephalus glaber OX=10181 GN=GW7_11265 PE=3 SV=1
**G5BD70**	Adenosylhomocysteinase OS=Heterocephalus glaber OX=10181 GN=GW7_07805 PE=3 SV=1
**G5BH50**	Profilin (Fragment) OS=Heterocephalus glaber OX=10181 GN=GW7_12907 PE=3 SV=1
**G5BIH8**	Protein kinase C OS=Heterocephalus glaber OX=10181 GN=GW7_09221 PE=3 SV=1
**G5BZ00**	Zinc transporter 3 OS=Heterocephalus glaber OX=10181 GN=GW7_14384 PE=4 SV=1
**G5AY28**	Synaptotagmin-17 OS=Heterocephalus glaber OX=10181 GN=GW7_06689 PE=4 SV=1
**G5BPT7**	Adenosylhomocysteinase OS=Heterocephalus glaber OX=10181 GN=AHCY PE=3 SV=1
**G5BF97**	DNA topoisomerase 2-beta OS=Heterocephalus glaber OX=10181 GN=GW7_15047 PE=4 SV=1
**G5AWL7**	Serine/threonine-protein phosphatase (Fragment) OS=Heterocephalus glaber OX=10181 GN=GW7_03093 PE=3 SV=1
**G5C1E9**	Leucine-rich repeat and calponin-like protein domain-containing protein 1 OS=Heterocephalus glaber OX=10181 GN=GW7_16595 PE=4 SV=1
**G5BSE8**	Histone H2A OS=Heterocephalus glaber OX=10181 GN=GW7_09999 PE=3 SV=1
**G5BFL5**	Glucose-6-phosphate 1-dehydrogenase OS=Heterocephalus glaber OX=10181 GN=GW7_19334 PE=3 SV=1
**G5ATY3**	Anion exchange protein OS=Heterocephalus glaber OX=10181 GN=GW7_14415 PE=3 SV=1
**G5BS07**	Dipeptidyl aminopeptidase-like protein 6 OS=Heterocephalus glaber OX=10181 GN=GW7_03940 PE=3 SV=1
**G5AYT2**	Putative ATP-dependent RNA helicase DDX17 OS=Heterocephalus glaber OX=10181 GN=GW7_02579 PE=3 SV=1
**G5BHI4**	NADH dehydrogenase [ubiquinone] 1 alpha subcomplex subunit 10, mitochondrial OS=Heterocephalus glaber OX=10181 GN=GW7_08787 PE=3 SV=1
**G5C873**	Neural cell adhesion molecule 2 OS=Heterocephalus glaber OX=10181 GN=GW7_07725 PE=4 SV=1
**G5B0L4**	ATP-citrate synthase OS=Heterocephalus glaber OX=10181 GN=GW7_06867 PE=3 SV=1
**G5CBJ9**	Dihydropyrimidinase-related protein 5 OS=Heterocephalus glaber OX=10181 GN=GW7_17031 PE=4 SV=1
**G5AXT3**	Adenylyl cyclase-associated protein OS=Heterocephalus glaber OX=10181 GN=GW7_06940 PE=3 SV=1
**G5CAD2**	Phosphatidylinositol-binding clathrin assembly protein OS=Heterocephalus glaber OX=10181 GN=GW7_14144 PE=4 SV=1
**G5B8C9**	OCIA domain-containing protein 2 (Fragment) OS=Heterocephalus glaber OX=10181 GN=GW7_00862 PE=4 SV=1
**G5BFV5**	Heterogeneous nuclear ribonucleoproteins C1/C2 OS=Heterocephalus glaber OX=10181 GN=GW7_08510 PE=4 SV=1
**G5BHY5**	60S ribosomal protein L11 (Fragment) OS=Heterocephalus glaber OX=10181 GN=GW7_07495 PE=3 SV=1
**G5C289**	Microtubule-associated protein RP/EB family member 2 (Fragment) OS=Heterocephalus glaber OX=10181 GN=GW7_13100 PE=4 SV=1
**G5C5Z4**	Actin-related protein 2 OS=Heterocephalus glaber OX=10181 GN=GW7_18355 PE=3 SV=1
**G5B8J0**	Noelin (Fragment) OS=Heterocephalus glaber OX=10181 GN=GW7_14332 PE=4 SV=1
**G5BH87**	Adenosylhomocysteinase (Fragment) OS=Heterocephalus glaber OX=10181 GN=GW7_16791 PE=3 SV=1
**G5AYJ7**	4F2 cell-surface antigen heavy chain OS=Heterocephalus glaber OX=10181 GN=GW7_01791 PE=4 SV=1
**G5BF08**	Homer protein-like protein 2 OS=Heterocephalus glaber OX=10181 GN=GW7_13593 PE=4 SV=1
**G5BUW4**	60S ribosomal protein L27a OS=Heterocephalus glaber OX=10181 GN=RPL27A PE=3 SV=1
**G5BM53**	Protein disulfide-isomerase OS=Heterocephalus glaber OX=10181 GN=GW7_18775 PE=3 SV=1
**G5BVG8**	Succinate--CoA ligase [ADP/GDP-forming] subunit alpha, mitochondrial OS=Heterocephalus glaber OX=10181 GN=SUCLG1 PE=3 SV=1
**G5AUK3**	Proteasome subunit alpha type (Fragment) OS=Heterocephalus glaber OX=10181 GN=GW7_10861 PE=3 SV=1
**G5BDX2**	Proteasome subunit alpha type OS=Heterocephalus glaber OX=10181 GN=PSMA7 PE=3 SV=1
**G5ALG5**	U2-associated protein SR140 OS=Heterocephalus glaber OX=10181 GN=GW7_01159 PE=4 SV=1

**G5B521**	Malic enzyme (Fragment) OS=Heterocephalus glaber OX=10181 GN=GW7_17644 PE=3 SV=1
**G5BV28**	Histone H3 OS=Heterocephalus glaber OX=10181 GN=GW7_16515 PE=3 SV=1
**G5AR38**	Endoplasmin (Fragment) OS=Heterocephalus glaber OX=10181 GN=GW7_10317 PE=3 SV=1
**G5BBE6**	CUG-BP-and ETR-3-like factor 2 (Fragment) OS=Heterocephalus glaber OX=10181 GN=GW7_19731 PE=4 SV=1
**G5BQU1**	Neural cell adhesion molecule L1 (Fragment) OS=Heterocephalus glaber OX=10181 GN=GW7_02026 PE=4 SV=1
**G5BY03**	Neuronal membrane glycoprotein M6-a OS=Heterocephalus glaber OX=10181 GN=GW7_21848 PE=4 SV=1
**G5C0C5**	Phosphoglycerate mutase OS=Heterocephalus glaber OX=10181 GN=GW7_09237 PE=3 SV=1
**G5BIK6**	Calcium/calmodulin-dependent protein kinase type II beta chain OS=Heterocephalus glaber OX=10181 GN=GW7_08267 PE=4 SV=1
**G5BF40**	Ras-related C3 botulinum toxin substrate 1 (Fragment) OS=Heterocephalus glaber OX=10181 GN=GW7_17917 PE=4 SV=1
**G5AV13**	Sodium/calcium exchanger 2 OS=Heterocephalus glaber OX=10181 GN=GW7_18138 PE=3 SV=1
**G5BRF2**	Neuroplastin OS=Heterocephalus glaber OX=10181 GN=GW7_19526 PE=4 SV=1
**G5BY29**	Importin subunit beta-1 OS=Heterocephalus glaber OX=10181 GN=GW7_00453 PE=4 SV=1
**G5ASA2**	Ras-related protein Rab-6A OS=Heterocephalus glaber OX=10181 GN=GW7_02105 PE=4 SV=1
**G5B9Q1**	Phytanoyl-CoA hydroxylase-interacting protein OS=Heterocephalus glaber OX=10181 GN=GW7_20727 PE=4 SV=1
**G5B0K5**	Ras-related protein Rab-5C OS=Heterocephalus glaber OX=10181 GN=GW7_06858 PE=4 SV=1
**G5BAV0**	Ras-related C3 botulinum toxin substrate 2 OS=Heterocephalus glaber OX=10181 GN=GW7_03672 PE=4 SV=1
**G5CBD1**	Beta-soluble NSF attachment protein OS=Heterocephalus glaber OX=10181 GN=GW7_00718 PE=4 SV=1
**G5B6J0**	Cofilin-1 OS=Heterocephalus glaber OX=10181 GN=GW7_20612 PE=3 SV=1
**G5BPR0**	Neuronal-specific septin-3 (Fragment) OS=Heterocephalus glaber OX=10181 GN=GW7_19448 PE=3 SV=1
**G5B8B7**	Clathrin coat assembly protein AP180 OS=Heterocephalus glaber OX=10181 GN=GW7_19308 PE=4 SV=1
**G5C3T1**	Actin-related protein 3 OS=Heterocephalus glaber OX=10181 GN=GW7_07463 PE=3 SV=1
**G5BHZ1**	Heterogeneous nuclear ribonucleoprotein R OS=Heterocephalus glaber OX=10181 GN=GW7_07501 PE=4 SV=1
**G5BM50**	Microtubule-associated protein 1A OS=Heterocephalus glaber OX=10181 GN=GW7_18772 PE=4 SV=1
**G5BBE2**	Glutamate--cysteine ligase catalytic subunit (Fragment) OS=Heterocephalus glaber OX=10181 GN=GW7_06303 PE=4 SV=1
**G5B8E6**	F-actin-capping protein subunit alpha (Fragment) OS=Heterocephalus glaber OX=10181 GN=GW7_12267 PE=3 SV=1
**G5AP97**	Protein 4.1 OS=Heterocephalus glaber OX=10181 GN=GW7_07130 PE=4 SV=1
**G5BDS2**	Cerebellin-3 OS=Heterocephalus glaber OX=10181 GN=GW7_16489 PE=4 SV=1
**G5BYY8**	Microtubule-associated protein RP/EB family member 3 OS=Heterocephalus glaber OX=10181 GN=MAPRE3 PE=4 SV=1
**G5B4V6**	Major prion protein OS=Heterocephalus glaber OX=10181 GN=GW7_03223 PE=3 SV=1
**G5AS68**	Phosphodiesterase OS=Heterocephalus glaber OX=10181 GN=GW7_02116 PE=3 SV=1
**G5AN52**	Ras-related protein Rab-11A OS=Heterocephalus glaber OX=10181 GN=RAB11A PE=4 SV=1
**G5AW15**	Vesicle-trafficking protein SEC22b OS=Heterocephalus glaber OX=10181 GN=GW7_13757 PE=3 SV=1
**G5BK72**	Creatine kinase S-type, mitochondrial OS=Heterocephalus glaber OX=10181 GN=GW7_10879 PE=3 SV=1
**G5BQA9**	Serotransferrin OS=Heterocephalus glaber OX=10181 GN=GW7_05106 PE=3 SV=1
**G5CA61**	Four and a half LIM domains protein 1 OS=Heterocephalus glaber OX=10181 GN=GW7_15573 PE=4 SV=1
**G5AQT3**	Septin-9 (Fragment) OS=Heterocephalus glaber OX=10181 GN=GW7_06407 PE=3 SV=1
**G5C6C2**	Nucleoprotein TPR OS=Heterocephalus glaber OX=10181 GN=GW7_07103 PE=4 SV=1
**G5C6M4**	Casein kinase II subunit alpha OS=Heterocephalus glaber OX=10181 GN=GW7_20923 PE=4 SV=1
**G5BJG1**	Cytochrome c oxidase subunit 4 isoform 1, mitochondrial OS=Heterocephalus glaber OX=10181 GN=GW7_12651 PE=4 SV=1
**G5BGK6**	Glutaminase kidney isoform, mitochondrial (Fragment) OS=Heterocephalus glaber OX=10181 GN=GW7_01412 PE=3 SV=1
**G5BMR5**	Guanine nucleotide-binding protein G(O) subunit alpha OS=Heterocephalus glaber OX=10181 GN=GW7_09345 PE=4 SV=1
**G5ALF6**	Phosphoglycerate kinase OS=Heterocephalus glaber OX=10181 GN=PGK1 PE=3 SV=1
**G5BJ78**	Rho GDP-dissociation inhibitor 1 OS=Heterocephalus glaber OX=10181 GN=ARHGDIA PE=4 SV=1
**G5AMA2**	NAD-dependent protein deacetylase OS=Heterocephalus glaber OX=10181 GN=GW7_16704 PE=3 SV=1
**G5AYH2**	Elongation factor 1-gamma OS=Heterocephalus glaber OX=10181 GN=GW7_01766 PE=4 SV=1
**G5APW6**	NipSnap-like protein 1 (Fragment) OS=Heterocephalus glaber OX=10181 GN=GW7_05309 PE=4 SV=1

**G5BVI3**	60S ribosomal protein L23a OS=Heterocephalus glaber OX=10181 GN=GW7_08185 PE=3 SV=1
**G5BYE6**	Endophilin-A1 (Fragment) OS=Heterocephalus glaber OX=10181 GN=GW7_18987 PE=4 SV=1
**G5AWP7**	GTP-binding nuclear protein Ran (Fragment) OS=Heterocephalus glaber OX=10181 GN=GW7_03170 PE=3 SV=1
**G5C662**	V-type proton ATPase subunit OS=Heterocephalus glaber OX=10181 GN=GW7_02358 PE=3 SV=1
**G5BG31**	MICOS complex subunit MIC60 OS=Heterocephalus glaber OX=10181 GN=IMMT PE=3 SV=1
**G5BI03**	Cell division control protein 42-like protein OS=Heterocephalus glaber OX=10181 GN=GW7_07513 PE=4 SV=1
**G5BCT6**	Platelet-activating factor acetylhydrolase IB subunit alpha OS=Heterocephalus glaber OX=10181 GN=GW7_05075 PE=3 SV=1
**G5BK64**	Phosphatidylinositol-5-phosphate 4-kinase type-2 beta (Fragment) OS=Heterocephalus glaber OX=10181 GN=GW7_13066 PE=4 SV=1
**G5BSC6**	Ubiquitin carboxyl-terminal hydrolase 7 OS=Heterocephalus glaber OX=10181 GN=GW7_08972 PE=3 SV=1
**G5BXL7**	Glucose-6-phosphate isomerase OS=Heterocephalus glaber OX=10181 GN=GW7_18400 PE=3 SV=1
**G5AN56**	Very-long-chain (3R)-3-hydroxyacyl-CoA dehydratase (Fragment) OS=Heterocephalus glaber OX=10181 GN=GW7_19668 PE=3 SV=1
**G5BC67**	T-complex protein 1 subunit alpha OS=Heterocephalus glaber OX=10181 GN=GW7_11562 PE=3 SV=1
**G5BML0**	Pleiotropic regulator 1 OS=Heterocephalus glaber OX=10181 GN=GW7_21603 PE=4 SV=1
**G5C1J8**	Neurexin-1-alpha OS=Heterocephalus glaber OX=10181 GN=GW7_16388 PE=4 SV=1
**G5AP00**	Polyadenylate-binding protein OS=Heterocephalus glaber OX=10181 GN=GW7_00765 PE=3 SV=1
**G5B8N7**	Vesicular glutamate transporter 1 OS=Heterocephalus glaber OX=10181 GN=GW7_15284 PE=4 SV=1
**G5BZX7**	CaM kinase-like vesicle-associated protein OS=Heterocephalus glaber OX=10181 GN=GW7_19932 PE=4 SV=1
**G5BFY1**	Inactive hydroxysteroid dehydrogenase-like protein 1 OS=Heterocephalus glaber OX=10181 GN=GW7_15943 PE=3 SV=1
**G5C8Q4**	FAST kinase domain-containing protein 1 OS=Heterocephalus glaber OX=10181 GN=GW7_06207 PE=4 SV=1
**G5BY67**	28S ribosomal protein S34, mitochondrial OS=Heterocephalus glaber OX=10181 GN=GW7_05752 PE=4 SV=1
**G5ARG8**	Heat shock 70 kDa protein 12A OS=Heterocephalus glaber OX=10181 GN=GW7_21295 PE=4 SV=1
**G5C8J0**	Netrin-G1 ligand OS=Heterocephalus glaber OX=10181 GN=GW7_15729 PE=4 SV=1
**G5B6G5**	Reticulon OS=Heterocephalus glaber OX=10181 GN=GW7_20694 PE=4 SV=1
**G5C5Z1**	Amino acid transporter OS=Heterocephalus glaber OX=10181 GN=GW7_18352 PE=3 SV=1
**G5APK7**	Zinc transporter ZIP10 OS=Heterocephalus glaber OX=10181 GN=GW7_00937 PE=4 SV=1
**G5AQ53**	Mitochondrial pyruvate carrier OS=Heterocephalus glaber OX=10181 GN=GW7_21171 PE=3 SV=1
**G5C384**	Pre-mRNA branch site protein p14 OS=Heterocephalus glaber OX=10181 GN=GW7_13983 PE=4 SV=1
**G5BAT4**	Desmoplakin OS=Heterocephalus glaber OX=10181 GN=GW7_06449 PE=4 SV=1
**G5BFD9**	Zinc transporter ZIP12 OS=Heterocephalus glaber OX=10181 GN=GW7_20294 PE=4 SV=1
**G5C2S9**	Heterogeneous nuclear ribonucleoprotein H2 OS=Heterocephalus glaber OX=10181 GN=GW7_14140 PE=4 SV=1
**G5BSL6**	Eukaryotic translation initiation factor 3 subunit E OS=Heterocephalus glaber OX=10181 GN=EIF3E PE=3 SV=1
**G5C226**	Sodium-and chloride-dependent GABA transporter 3 OS=Heterocephalus glaber OX=10181 GN=GW7_20151 PE=4 SV=1
**G5B4Q7**	Arf-GAP with SH3 domain, ANK repeat and PH domain-containing protein 1 OS=Heterocephalus glaber OX=10181 GN=GW7_10926 PE=4 SV=1
**G5B2A2**	Copine-6 OS=Heterocephalus glaber OX=10181 GN=GW7_11974 PE=4 SV=1
**G5BXJ3**	Myelin-associated glycoprotein isoform b OS=Heterocephalus glaber OX=10181 GN=MAG PE=4 SV=1
**G5AX92**	Pentatricopeptide repeat-containing protein 1 OS=Heterocephalus glaber OX=10181 GN=GW7_06631 PE=4 SV=1
**G5AXV9**	Ezrin OS=Heterocephalus glaber OX=10181 GN=GW7_19302 PE=4 SV=1
**G5C3G6**	Heat shock 70 kDa protein 1B OS=Heterocephalus glaber OX=10181 GN=GW7_04796 PE=3 SV=1
**G5BX86**	Tubulin monoglycylase TTLL3 OS=Heterocephalus glaber OX=10181 GN=GW7_19486 PE=4 SV=1
**G5BET5**	ATP-dependent RNA helicase DDX1 OS=Heterocephalus glaber OX=10181 GN=GW7_19785 PE=4 SV=1
**G5B782**	60S acidic ribosomal protein P0 OS=Heterocephalus glaber OX=10181 GN=RPLP0 PE=3 SV=1
**G5AUL0**	Isocitrate dehydrogenase [NAD] subunit, mitochondrial OS=Heterocephalus glaber OX=10181 GN=GW7_10868 PE=3 SV=1
**G5CAJ1**	Up-regulated during skeletal muscle growth protein 5 OS=Heterocephalus glaber OX=10181 GN=GW7_15920 PE=4 SV=1
**G5AJZ7**	Phosphatidylinositol-4-phosphate 5-kinase type-1 alpha OS=Heterocephalus glaber OX=10181 GN=GW7_10556 PE=4 SV=1
**G5AKW2**	RasGAP-activating-like protein 1 OS=Heterocephalus glaber OX=10181 GN=GW7_10151 PE=4 SV=1

**G5BSB5**	G protein-coupled receptor kinase (Fragment) OS=Heterocephalus glaber OX=10181 GN=GW7_14698 PE=3 SV=1
**G5C6E9**	Myotubularin OS=Heterocephalus glaber OX=10181 GN=GW7_05400 PE=4 SV=1
**G5CBK6**	Trifunctional enzyme subunit beta, mitochondrial OS=Heterocephalus glaber OX=10181 GN=GW7_17038 PE=3 SV=1
**G5AN45**	NADH dehydrogenase [ubiquinone] 1 alpha subcomplex subunit 13 OS=Heterocephalus glaber OX=10181 GN=GW7_05858 PE=4 SV=1
**G5AX86**	E3 ubiquitin-protein ligase OS=Heterocephalus glaber OX=10181 GN=GW7_06625 PE=4 SV=1
**G5BYB7**	UPF0420 protein C16orf58 OS=Heterocephalus glaber OX=10181 GN=GW7_05117 PE=4 SV=1
**G5BLW2**	Glutamate receptor 2 OS=Heterocephalus glaber OX=10181 GN=GW7_04537 PE=3 SV=1
**G5BKB7**	Gamma-adducin OS=Heterocephalus glaber OX=10181 GN=GW7_14624 PE=4 SV=1
**G5BWQ7**	High affinity copper uptake protein 1 OS=Heterocephalus glaber OX=10181 GN=GW7_09545 PE=4 SV=1
**G5ANZ0**	Cytochrome c oxidase subunit 6C OS=Heterocephalus glaber OX=10181 GN=GW7_00755 PE=4 SV=1
**G5CBK7**	Trifunctional enzyme subunit alpha, mitochondrial OS=Heterocephalus glaber OX=10181 GN=HADHA PE=3 SV=1
**G5ANV7**	Phosphoglycerate mutase family member 5 OS=Heterocephalus glaber OX=10181 GN=GW7_09572 PE=4 SV=1
**G5APR8**	BTB/POZ domain-containing protein KCTD16 OS=Heterocephalus glaber OX=10181 GN=GW7_01638 PE=4 SV=1
**G5AW66**	NADH dehydrogenase [ubiquinone] 1 subunit C2 OS=Heterocephalus glaber OX=10181 GN=GW7_00914 PE=3 SV=1
**G5BBX1**	Calcium-dependent secretion activator 1 (Fragment) OS=Heterocephalus glaber OX=10181 GN=GW7_02265 PE=4 SV=1
**G5C351**	FERM, RhoGEF and pleckstrin domain-containing protein 1 (Fragment) OS=Heterocephalus glaber OX=10181 GN=GW7_12715 PE=4 SV=1
**G5BZN6**	Succinate dehydrogenase [ubiquinone] iron-sulfur subunit, mitochondrial OS=Heterocephalus glaber OX=10181 GN=GW7_09699 PE=3 SV=1
**G5B5W3**	Alanyl-tRNA synthetase, cytoplasmic OS=Heterocephalus glaber OX=10181 GN=AARS PE=3 SV=1
**G5B6C9**	40S ribosomal protein S3a OS=Heterocephalus glaber OX=10181 GN=GW7_08253 PE=4 SV=1
**G5C077**	AFG3-like protein 2 (Fragment) OS=Heterocephalus glaber OX=10181 GN=GW7_12057 PE=3 SV=1
**G5BPY0**	Sidoreflexin OS=Heterocephalus glaber OX=10181 GN=SFXN1 PE=3 SV=1
**G5BD34**	C-4 methylsterol oxidase OS=Heterocephalus glaber OX=10181 GN=GW7_07430 PE=3 SV=1
**G5BBH0**	Ras-related protein Rap-1b isoform 1 OS=Heterocephalus glaber OX=10181 GN=RAP1B PE=4 SV=1
**G5B5J9**	Tyrosine--tRNA ligase OS=Heterocephalus glaber OX=10181 GN=GW7_11504 PE=3 SV=1
**G5C0D0**	Neuronal cell adhesion molecule OS=Heterocephalus glaber OX=10181 GN=GW7_06596 PE=4 SV=1
**G5BRW3**	Ribosomal protein L19 OS=Heterocephalus glaber OX=10181 GN=GW7_09589 PE=3 SV=1
**G5C7R9**	Proteasome subunit beta OS=Heterocephalus glaber OX=10181 GN=PSMB1 PE=3 SV=1
**G5BRG5**	Semaphorin-7A OS=Heterocephalus glaber OX=10181 GN=GW7_19539 PE=3 SV=1
**G5B6P2**	Neurexin-2-alpha OS=Heterocephalus glaber OX=10181 GN=GW7_20664 PE=4 SV=1
**G5ARH5**	Gamma-aminobutyric acid receptor subunit beta-2 OS=Heterocephalus glaber OX=10181 GN=GW7_13529 PE=3 SV=1
**G5AZL1**	40S ribosomal protein S15 OS=Heterocephalus glaber OX=10181 GN=RPS15 PE=3 SV=1
**G5BYW2**	Phenylalanyl-tRNA synthetase alpha chain OS=Heterocephalus glaber OX=10181 GN=GW7_16082 PE=4 SV=1
**G5B5D5**	Sodium/potassium-transporting ATPase subunit beta OS=Heterocephalus glaber OX=10181 GN=GW7_13837 PE=3 SV=1
**G5BNW3**	Rho-related GTP-binding protein RhoC OS=Heterocephalus glaber OX=10181 GN=GW7_03049 PE=4 SV=1
**G5B2L9**	Rogdi-like protein OS=Heterocephalus glaber OX=10181 GN=GW7_19978 PE=4 SV=1
**G5C225**	Transporter OS=Heterocephalus glaber OX=10181 GN=GW7_20150 PE=3 SV=1
**G5BT86**	Kininogen-1 OS=Heterocephalus glaber OX=10181 GN=KNG1 PE=4 SV=1
**G5B4Y6**	Beta-adducin OS=Heterocephalus glaber OX=10181 GN=ADD2 PE=4 SV=1
**G5BYS2**	NADH dehydrogenase [ubiquinone] 1 beta subcomplex subunit 7 OS=Heterocephalus glaber OX=10181 GN=GW7_16042 PE=4 SV=1
**G5AKN0**	N-acylethanolamine-hydrolyzing acid amidase OS=Heterocephalus glaber OX=10181 GN=GW7_21291 PE=3 SV=1
**G5BEP8**	Tyrosine-protein phosphatase non-receptor type substrate 1 OS=Heterocephalus glaber OX=10181 GN=GW7_13443 PE=4 SV=1
**G5BRJ4**	Induced myeloid leukemia cell differentiation protein Mcl-1-like protein OS=Heterocephalus glaber OX=10181 GN=GW7_20578 PE=4 SV=1
**G5CPH0**	ATP synthase subunit a OS=Heterocephalus glaber OX=10181 GN=ATP6 PE=4 SV=1

**G5BV92**	Mu-crystallin-like protein OS=Heterocephalus glaber OX=10181 GN=CRYM PE=4 SV=1
**G5AQL0**	Hydroxyacyl-coenzyme A dehydrogenase, mitochondrial OS=Heterocephalus glaber OX=10181 GN=GW7_17857 PE=4 SV=1
**G5BWU7**	Catenin alpha-2 (Fragment) OS=Heterocephalus glaber OX=10181 GN=GW7_19189 PE=4 SV=1
**G5B6W3**	60S ribosomal protein L35 OS=Heterocephalus glaber OX=10181 GN=RPL35 PE=3 SV=1
**G5APV2**	Splicing factor 3 subunit 1 OS=Heterocephalus glaber OX=10181 GN=SF3A1 PE=4 SV=1
**G5API3**	60S ribosomal protein L13 OS=Heterocephalus glaber OX=10181 GN=RPL13 PE=3 SV=1
**G5AYQ6**	Zinc transporter 7 OS=Heterocephalus glaber OX=10181 GN=GW7_21185 PE=4 SV=1
**G5BX63**	NADH dehydrogenase (Ubiquinone) 1 beta subcomplex subunit 5, mitochondrial isoform 1 OS=Heterocephalus glaber OX=10181 GN=NDUFB5 PE=4 SV=1
**G5BCR1**	NAD(P) transhydrogenase, mitochondrial OS=Heterocephalus glaber OX=10181 GN=GW7_04058 PE=4 SV=1
**G5BWN6**	V-type proton ATPase subunit E 1 isoform b OS=Heterocephalus glaber OX=10181 GN=ATP6V1E1 PE=3 SV=1
**G5AWB5**	Talin-2 OS=Heterocephalus glaber OX=10181 GN=GW7_00150 PE=4 SV=1
**G5ATW7**	Carbonic anhydrase 2 (Fragment) OS=Heterocephalus glaber OX=10181 GN=GW7_14399 PE=4 SV=1
**G5BCE5**	Peroxiredoxin-4 OS=Heterocephalus glaber OX=10181 GN=PRDX4 PE=4 SV=1
**G5AV28**	Synaptic vesicle glycoprotein 2B OS=Heterocephalus glaber OX=10181 GN=GW7_05778 PE=4 SV=1
**G5BN12**	Serine/threonine-protein phosphatase OS=Heterocephalus glaber OX=10181 GN=PPP2CA PE=3 SV=1
**G5BGY3**	Phenylalanine--tRNA ligase beta subunit OS=Heterocephalus glaber OX=10181 GN=FARSB PE=4 SV=1
**G5B5Y3**	40S ribosomal protein S4 OS=Heterocephalus glaber OX=10181 GN=GW7_15480 PE=3 SV=1
**G5B4R7**	Small nuclear ribonucleoprotein-associated protein B (Fragment) OS=Heterocephalus glaber OX=10181 GN=GW7_03184 PE=4 SV=1
**G5BYP8**	Bromodomain-containing protein 4 OS=Heterocephalus glaber OX=10181 GN=GW7_16018 PE=4 SV=1
**G5AW20**	Lupus La protein-like protein OS=Heterocephalus glaber OX=10181 GN=GW7_02256 PE=4 SV=1
**G5AZ44**	Putative cysteinyl-tRNA synthetase, mitochondrial OS=Heterocephalus glaber OX=10181 GN=GW7_00286 PE=3 SV=1
**G5BAR1**	Cytosolic non-specific dipeptidase OS=Heterocephalus glaber OX=10181 GN=GW7_18554 PE=4 SV=1
**G5BLB9**	Phytanoyl-CoA hydroxylase-interacting protein-like isoform 1 OS=Heterocephalus glaber OX=10181 GN=PHYHIPL PE=4 SV=1
**G5C720**	Glycogen debranching enzyme OS=Heterocephalus glaber OX=10181 GN=GW7_08051 PE=4 SV=1
**G5B6T9**	Amphiphysin OS=Heterocephalus glaber OX=10181 GN=GW7_09869 PE=4 SV=1
**G5C053**	Clathrin heavy chain OS=Heterocephalus glaber OX=10181 GN=GW7_01835 PE=3 SV=1
**G5BL29**	Fumarate hydratase, mitochondrial OS=Heterocephalus glaber OX=10181 GN=FH PE=3 SV=1
**G5BQW4**	Proteasome subunit alpha type OS=Heterocephalus glaber OX=10181 GN=PSMA5 PE=3 SV=1
**G5B4D9**	Small conductance calcium-activated potassium channel protein 1 (Fragment) OS=Heterocephalus glaber OX=10181 GN=GW7_16479 PE=4 SV=1
**G5BNX8**	ATP synthase subunit b, mitochondrial OS=Heterocephalus glaber OX=10181 GN=GW7_03064 PE=4 SV=1
**G5ATA4**	Collagen alpha-1(XVIII) chain OS=Heterocephalus glaber OX=10181 GN=GW7_13728 PE=4 SV=1
**G5CBM7**	Apolipoprotein E OS=Heterocephalus glaber OX=10181 GN=GW7_18161 PE=2 SV=1
**G5BCD2**	Asparaginyl-tRNA synthetase, cytoplasmic OS=Heterocephalus glaber OX=10181 GN=GW7_03772 PE=4 SV=1
**G5BRC3**	Coronin OS=Heterocephalus glaber OX=10181 GN=GW7_14880 PE=3 SV=1
**G5BIL3**	Peptidyl-prolyl cis-trans isomerase OS=Heterocephalus glaber OX=10181 GN=PPIA PE=3 SV=1
**G5ARB5**	Histone-lysine N-methyltransferase OS=Heterocephalus glaber OX=10181 GN=GW7_14304 PE=4 SV=1
**G5BPH4**	Tubulin polymerization-promoting protein OS=Heterocephalus glaber OX=10181 GN=GW7_14713 PE=4 SV=1
**E3VX74**	40S ribosomal protein S27 OS=Heterocephalus glaber OX=10181 GN=GW7_11176 PE=2 SV=1
**G5AU15**	Pre-mRNA-processing factor 40-like protein B OS=Heterocephalus glaber OX=10181 GN=GW7_14447 PE=4 SV=1
**G5BH25**	Splicing factor 3B subunit 4 OS=Heterocephalus glaber OX=10181 GN=GW7_05788 PE=4 SV=1
**G5BEC8**	Phosphatidylinositol-5-phosphate 4-kinase type-2 gamma OS=Heterocephalus glaber OX=10181 GN=GW7_04681 PE=4 SV=1
**G5BFF6**	Cytochrome b-c1 complex subunit 8 OS=Heterocephalus glaber OX=10181 GN=UQCRQ PE=4 SV=1
**G5AXV0**	Catalase OS=Heterocephalus glaber OX=10181 GN=GW7_08865 PE=3 SV=1
**G5ATA6**	Poly(RC)-binding protein 3 OS=Heterocephalus glaber OX=10181 GN=GW7_13730 PE=4 SV=1
**G5AY95**	NADH dehydrogenase (Ubiquinone) 1 beta subcomplex subunit 9 OS=Heterocephalus glaber OX=10181 GN=NDUFB9 PE=3 SV=1
**G5AZF7**	NADH dehydrogenase [ubiquinone] 1 beta subcomplex subunit 11, mitochondrial OS=Heterocephalus glaber OX=10181 GN=GW7_11280 PE=4 SV=1
**G5BUE2**	Proteasome subunit alpha type (Fragment) OS=Heterocephalus glaber OX=10181 GN=GW7_21362 PE=3 SV=1
**G5BGU2**	Regulator of G-protein signaling 7 OS=Heterocephalus glaber OX=10181 GN=GW7_20225 PE=4 SV=1

**G5BMM2**	Pleckstrin-like protein domain-containing family A member 6 OS=Heterocephalus glaber OX=10181 GN=GW7_11215 PE=4 SV=1
**G5BCG0**	Protein kinase C and casein kinase substrate in neurons protein 1 OS=Heterocephalus glaber OX=10181 GN=GW7_15650 PE=4 SV=1
**G5BVV5**	Eukaryotic translation initiation factor 3 subunit A OS=Heterocephalus glaber OX=10181 GN=EIF3A PE=3 SV=1
**G5AS76**	40S ribosomal protein S3 OS=Heterocephalus glaber OX=10181 GN=RPS3 PE=3 SV=1
**G5C4R0**	Succinyl-CoA:3-ketoacid-coenzyme A transferase OS=Heterocephalus glaber OX=10181 GN=GW7_19761 PE=3 SV=1
**G5AR57**	NADH dehydrogenase [ubiquinone] 1 alpha subcomplex subunit 2 (Fragment) OS=Heterocephalus glaber OX=10181 GN=GW7_12539 PE=4 SV=1
**G5B165**	Multidrug resistance-associated protein 4 OS=Heterocephalus glaber OX=10181 GN=ABCC4 PE=4 SV=1
**G5AZ20**	Gamma-aminobutyric acid type B receptor subunit 2 OS=Heterocephalus glaber OX=10181 GN=GW7_05037 PE=3 SV=1
**G5AKA6**	Adenylyl cyclase-associated protein OS=Heterocephalus glaber OX=10181 GN=GW7_18428 PE=3 SV=1
**G5C7L2**	40S ribosomal protein S7 OS=Heterocephalus glaber OX=10181 GN=RPS7 PE=3 SV=1
**G5BJM5**	60S ribosomal protein L12 OS=Heterocephalus glaber OX=10181 GN=GW7_00296 PE=3 SV=1
**G5CPG8**	Cytochrome c oxidase subunit 2 OS=Heterocephalus glaber OX=10181 GN=COX2 PE=3 SV=1
**G5B5Y6**	Lysine--tRNA ligase OS=Heterocephalus glaber OX=10181 GN=KARS PE=3 SV=1
**G5BWW8**	Splicing factor 3B subunit 2 OS=Heterocephalus glaber OX=10181 GN=GW7_07186 PE=4 SV=1
**G5B8P3**	40S ribosomal protein S11 (Fragment) OS=Heterocephalus glaber OX=10181 GN=GW7_15290 PE=3 SV=1
**G5AVA6**	Enoyl-CoA hydratase, mitochondrial OS=Heterocephalus glaber OX=10181 GN=GW7_07876 PE=3 SV=1
**G5AYV0**	Synaptogyrin-1 (Fragment) OS=Heterocephalus glaber OX=10181 GN=GW7_02597 PE=4 SV=1
**G5AYX6**	Pyridoxal phosphate phosphatase OS=Heterocephalus glaber OX=10181 GN=GW7_02557 PE=3 SV=1
**G5C0Q7**	MICOS complex subunit MIC13 OS=Heterocephalus glaber OX=10181 GN=GW7_20494 PE=3 SV=1
**G5BLB3**	CDGSH iron sulfur domain-containing protein 1 OS=Heterocephalus glaber OX=10181 GN=GW7_01960 PE=4 SV=1
**G5B7K2**	Calnexin OS=Heterocephalus glaber OX=10181 GN=GW7_17973 PE=3 SV=1
**G5B8W4**	40S ribosomal protein S27a OS=Heterocephalus glaber OX=10181 GN=RPS27A PE=4 SV=1
**G5BL47**	Cytochrome b-c1 complex subunit Rieske, mitochondrial OS=Heterocephalus glaber OX=10181 GN=UQCRFS1 PE=4 SV=1
**G5BN01**	Protein RUFY3 OS=Heterocephalus glaber OX=10181 GN=GW7_20521 PE=4 SV=1
**G5AWC6**	Transcription initiation factor IIA subunit 2 (Fragment) OS=Heterocephalus glaber OX=10181 GN=GW7_00161 PE=4 SV=1
**G5AZE3**	Receptor-type tyrosine-protein phosphatase zeta OS=Heterocephalus glaber OX=10181 GN=GW7_13190 PE=4 SV=1
**G5BAZ8**	Zinc transporter SLC39A7 OS=Heterocephalus glaber OX=10181 GN=SLC39A7 PE=4 SV=1
**G5BII7**	Dolichyl-diphosphooligosaccharide--protein glycosyltransferase subunit 1 OS=Heterocephalus glaber OX=10181 GN=GW7_10957 PE=3 SV=1
**G5AQU8**	Ubiquitin-conjugating enzyme E2 O OS=Heterocephalus glaber OX=10181 GN=GW7_06422 PE=4 SV=1
**G5BW10**	Epidermal growth factor receptor kinase substrate 8-like protein 1 OS=Heterocephalus glaber OX=10181 GN=GW7_11661 PE=4 SV=1
**G5BWA4**	Proteasome subunit beta type-6 OS=Heterocephalus glaber OX=10181 GN=GW7_04618 PE=4 SV=1
**G5B211**	28S ribosomal protein S27, mitochondrial (Fragment) OS=Heterocephalus glaber OX=10181 GN=GW7_19014 PE=4 SV=1
**G5BAA4**	Heterogeneous nuclear ribonucleoprotein A1-like protein 2 OS=Heterocephalus glaber OX=10181 GN=GW7_16956 PE=4 SV=1
**G5B7K7**	40S ribosomal protein S5 OS=Heterocephalus glaber OX=10181 GN=GW7_05598 PE=3 SV=1
**G5BFU9**	N6-adenosine-methyltransferase 70 kDa subunit OS=Heterocephalus glaber OX=10181 GN=METTL3 PE=3 SV=1
**G5BX57**	SLIT-ROBO Rho GTPase-activating protein 2 OS=Heterocephalus glaber OX=10181 GN=GW7_13782 PE=4 SV=1
**G5AKY1**	Neuronal membrane glycoprotein M6-b OS=Heterocephalus glaber OX=10181 GN=GW7_16652 PE=4 SV=1
**G5AVC6**	UDP-glucuronosyltransferase OS=Heterocephalus glaber OX=10181 GN=GW7_15973 PE=3 SV=1
**G5AL84**	Leucine-rich PPR motif-containing protein, mitochondrial (Fragment) OS=Heterocephalus glaber OX=10181 GN=GW7_03995 PE=4 SV=1
**G5AUX0**	Optic atrophy 3 protein OS=Heterocephalus glaber OX=10181 GN=GW7_18095 PE=4 SV=1
**G5B3V9**	Engulfment and cell motility protein 1 OS=Heterocephalus glaber OX=10181 GN=GW7_09458 PE=4 SV=1
**G5B6U4**	Ras-related protein Ral-A OS=Heterocephalus glaber OX=10181 GN=RALA PE=4 SV=1

**G5AV01**	AP complex subunit sigma OS=Heterocephalus glaber OX=10181 GN=GW7_18126 PE=3 SV=1
**G5C7Y3**	V-type proton ATPase subunit D OS=Heterocephalus glaber OX=10181 GN=ATP6V1D PE=4 SV=1
**G5B4M8**	ELAV-like protein (Fragment) OS=Heterocephalus glaber OX=10181 GN=GW7_02477 PE=3 SV=1
**G5C705**	Leucine-rich glioma-inactivated protein 1 OS=Heterocephalus glaber OX=10181 GN=LGI1 PE=4 SV=1
**G5BPE1**	tRNA-splicing ligase RtcB homolog OS=Heterocephalus glaber OX=10181 GN=RTCB PE=3 SV=1
**G5BK63**	Proteasome subunit beta OS=Heterocephalus glaber OX=10181 GN=GW7_13065 PE=3 SV=1
**G5BMY3**	Golgi SNAP receptor complex member 2 isoform A OS=Heterocephalus glaber OX=10181 GN=GOSR2 PE=3 SV=1
**G5BBC3**	Splicing factor 3A subunit 2 OS=Heterocephalus glaber OX=10181 GN=GW7_07321 PE=4 SV=1
**G5AVX4**	Phosphatidylinositol-5-phosphate 4-kinase type-2 alpha OS=Heterocephalus glaber OX=10181 GN=GW7_05903 PE=4 SV=1
**G5C0T9**	NADH dehydrogenase [ubiquinone] 1 alpha subcomplex subunit 12 OS=Heterocephalus glaber OX=10181 GN=NDUFA12 PE=3 SV=1
**G5C835**	Propionyl-CoA carboxylase beta chain, mitochondrial OS=Heterocephalus glaber OX=10181 GN=GW7_21808 PE=4 SV=1
**G5B795**	60S ribosomal protein L31 OS=Heterocephalus glaber OX=10181 GN=GW7_13242 PE=4 SV=1
**G5BPN1**	Solute carrier family 2, facilitated glucose transporter member 3 OS=Heterocephalus glaber OX=10181 GN=GW7_17311 PE=3 SV=1
**G5BTQ7**	Succinate--CoA ligase [ADP-forming] subunit beta, mitochondrial OS=Heterocephalus glaber OX=10181 GN=SUCLA2 PE=3 SV=1
**G5B6G2**	Non-specific serine/threonine protein kinase (Fragment) OS=Heterocephalus glaber OX=10181 GN=GW7_20691 PE=4 SV=1
**G5B224**	Hsp90 co-chaperone Cdc37 OS=Heterocephalus glaber OX=10181 GN=CDC37 PE=4 SV=1
**G5BBP0**	2,4-dienoyl-CoA reductase, mitochondrial OS=Heterocephalus glaber OX=10181 GN=GW7_09360 PE=4 SV=1
**G5BY63**	Hydroxyacylglutathione hydrolase, mitochondrial OS=Heterocephalus glaber OX=10181 GN=GW7_05748 PE=3 SV=1
**G5BFW4**	Protein NDRG2 OS=Heterocephalus glaber OX=10181 GN=GW7_08519 PE=4 SV=1
**G5BAG3**	RuvB-like helicase OS=Heterocephalus glaber OX=10181 GN=RUVBL2 PE=3 SV=1
**G5ASV2**	Nucleolin OS=Heterocephalus glaber OX=10181 GN=GW7_10373 PE=4 SV=1
**G5BC44**	Ras-related protein Rab-7a OS=Heterocephalus glaber OX=10181 GN=GW7_12906 PE=4 SV=1
**G5BF50**	Eukaryotic translation initiation factor 3 subunit B OS=Heterocephalus glaber OX=10181 GN=EIF3B PE=3 SV=1
**G5BHQ8**	Kalirin OS=Heterocephalus glaber OX=10181 GN=GW7_14844 PE=4 SV=1
**G5AYG1**	Cleavage and polyadenylation specificity factor subunit 7 OS=Heterocephalus glaber OX=10181 GN=GW7_01755 PE=4 SV=1
**G5B2H6**	Sodium/potassium/calcium exchanger 2 OS=Heterocephalus glaber OX=10181 GN=GW7_00607 PE=3 SV=1
