# Supplementary material for: Acute Hypoxia Alters Extracellular Vesicle Signatures and the Brain Citrullinome of Naked Mole-Rats (Heterocephalus glaber)
Source: Int J Mol Sci. 2022 Apr 23;23(9):4683. doi: 10.3390/ijms23094683 (PMC9100269; doi:10.3390/ijms23094683)
Supplement: Supplementary file 1 [file ijms-23-04683-s001.zip › Supp Table S2 Brain citrullinome hypoxia all with specific hits highlights (1).pdf]

Supplementary Table S2. The brain citrullinome in hypoxia. Full list of naked mole-rat protein hits from LC-MS/MS analysis of F95 enriched proteins isolated from brains of naked mole-rats (*Heterocephalus glaber*) following hypoxia; hits specific for the hypoxia group only are highlighted in pink.

Protein ID	Protein name
G5B8V8	Spectrin beta chain OS=Heterocephalus glaber OX=10181 GN=GW7_15253 PE=3 SV=1
G5ASE4	Spectrin alpha chain, brain OS=Heterocephalus glaber OX=10181 GN=GW7_01914 PE=4 SV=1
G5AZQ6	Sodium/potassium-transporting ATPase subunit alpha OS=Heterocephalus glaber OX=10181 GN=GW7_15424 PE=3 SV=1
G5C9E0	DmX-like protein 2 OS=Heterocephalus glaber OX=10181 GN=GW7_04224 PE=4 SV=1
G5BWK9	Sodium/potassium-transporting ATPase subunit alpha-2 OS=Heterocephalus glaber OX=10181 GN=GW7_00530 PE=3 SV=1
G5BFJ7	Sodium/potassium-transporting ATPase subunit alpha OS=Heterocephalus glaber OX=10181 GN=GW7_09834 PE=3 SV=1
G5BY99	Dihydropyrimidinase-related protein 2 (Fragment) OS=Heterocephalus glaber OX=10181 GN=GW7_07625 PE=4 SV=1
G5BL81	ATP synthase subunit alpha OS=Heterocephalus glaber OX=10181 GN=ATP5A1 PE=3 SV=1
G5BHB1	Pyruvate kinase OS=Heterocephalus glaber OX=10181 GN=GW7_06053 PE=3 SV=1
G5ASF4	Dynamin-1 (Fragment) OS=Heterocephalus glaber OX=10181 GN=GW7_01924 PE=3 SV=1
G5BS02	Heat shock cognate 71 kDa protein OS=Heterocephalus glaber OX=10181 GN=GW7_03309 PE=3 SV=1
G5BLH0	Syntaxin-binding protein 1 OS=Heterocephalus glaber OX=10181 GN=GW7_06738 PE=3 SV=1
G5C895	Clathrin heavy chain OS=Heterocephalus glaber OX=10181 GN=GW7_08300 PE=3 SV=1
G5B184	Hexokinase-1 OS=Heterocephalus glaber OX=10181 GN=GW7_10633 PE=3 SV=1
G5ATF9	Glutamate dehydrogenase 1, mitochondrial OS=Heterocephalus glaber OX=10181 GN=GW7_14620 PE=3 SV=1
G5BB67	ATP synthase subunit beta OS=Heterocephalus glaber OX=10181 GN=GW7_15746 PE=3 SV=1
G5B0C4	Synapsin-1 OS=Heterocephalus glaber OX=10181 GN=GW7_10284 PE=4 SV=1
G5ARR9	Tenascin-R OS=Heterocephalus glaber OX=10181 GN=GW7_21557 PE=4 SV=1
G5AST8	Homer protein-like protein 1 (Fragment) OS=Heterocephalus glaber OX=10181 GN=GW7_06603 PE=4 SV=1
G5ANG4	Calcium-binding mitochondrial carrier protein Aralar1 OS=Heterocephalus glaber OX=10181 GN=SLC25A12 PE=3 SV=1
G5BXS0	Fructose-bisphosphate aldolase OS=Heterocephalus glaber OX=10181 GN=GW7_19061 PE=3 SV=1
G5AU24	Tubulin alpha-1C chain OS=Heterocephalus glaber OX=10181 GN=GW7_14456 PE=3 SV=1
G5BPP6	Aconitate hydratase, mitochondrial OS=Heterocephalus glaber OX=10181 GN=GW7_19434 PE=3 SV=1
G5BNN8	Heat shock cognate protein HSP 90-beta OS=Heterocephalus glaber OX=10181 GN=GW7_19161 PE=3 SV=1
G5AKJ8	Tubulin alpha chain OS=Heterocephalus glaber OX=10181 GN=GW7_07278 PE=3 SV=1
G5BXB9	Transitional endoplasmic reticulum ATPase (Fragment) OS=Heterocephalus glaber OX=10181 GN=GW7_03434 PE=3 SV=1
G5BJV3	Alpha-1,4 glucan phosphorylase OS=Heterocephalus glaber OX=10181 GN=GW7_03733 PE=3 SV=1
G5B5P2	Serum albumin (Fragment) OS=Heterocephalus glaber OX=10181 GN=GW7_11087 PE=4 SV=1
G5BFZ1	Tubulin beta chain OS=Heterocephalus glaber OX=10181 GN=GW7_03924 PE=3 SV=1
G5BVT0	Malic enzyme OS=Heterocephalus glaber OX=10181 GN=GW7_16261 PE=3 SV=1
G5BVP2	ARF GTPase-activating protein GIT1 OS=Heterocephalus glaber OX=10181 GN=GW7_08203 PE=4 SV=1
G5B0L2	2',3'-cyclic-nucleotide 3'-phosphodiesterase (Fragment) OS=Heterocephalus glaber OX=10181 GN=GW7_06865 PE=4 SV=1
G5APH2	Tubulin beta chain OS=Heterocephalus glaber OX=10181 GN=GW7_12416 PE=3 SV=1
G5BTG2	Putative ATP-dependent RNA helicase DHX30 (Fragment) OS=Heterocephalus glaber OX=10181 GN=GW7_17258 PE=4 SV=1
G5ARA1	Lamin-A/C OS=Heterocephalus glaber OX=10181 GN=GW7_14290 PE=3 SV=1
G5C8F7	AP-2 complex subunit alpha OS=Heterocephalus glaber OX=10181 GN=GW7_12697 PE=3 SV=1

**G5C6D8**	Splicing factor, proline-and glutamine-rich OS=Heterocephalus glaber OX=10181 GN=GW7_05389 PE=4 SV=1
**G5B750**	AP complex subunit beta OS=Heterocephalus glaber OX=10181 GN=GW7_18272 PE=3 SV=1
**G5C546**	Alpha-enolase OS=Heterocephalus glaber OX=10181 GN=GW7_09136 PE=2 SV=1
**G5B8Q7**	AP-2 complex subunit alpha OS=Heterocephalus glaber OX=10181 GN=GW7_15304 PE=3 SV=1
**G5BJ63**	Actin, cytoplasmic 2 OS=Heterocephalus glaber OX=10181 GN=GW7_04078 PE=3 SV=1
**G5BWZ4**	Spectrin beta chain OS=Heterocephalus glaber OX=10181 GN=GW7_07212 PE=3 SV=1
**G5BW26**	Non-POU domain-containing octamer-binding protein OS=Heterocephalus glaber OX=10181 GN=NONO PE=4 SV=1
**G5BI78**	Actin, cytoplasmic 1 OS=Heterocephalus glaber OX=10181 GN=GW7_16883 PE=3 SV=1
**G5BVH5**	Fructose-bisphosphate aldolase OS=Heterocephalus glaber OX=10181 GN=GW7_08177 PE=3 SV=1
**G5BD04**	Heat shock-related 70 kDa protein 2 OS=Heterocephalus glaber OX=10181 GN=GW7_17513 PE=3 SV=1
**G5BTH0**	Dihydropyrimidinase-related protein 3 OS=Heterocephalus glaber OX=10181 GN=DPYSL3 PE=4 SV=1
**G5BMY6**	Vesicle-fusing ATPase (Fragment) OS=Heterocephalus glaber OX=10181 GN=GW7_07669 PE=4 SV=1
**G5AMA4**	Heterogeneous nuclear ribonucleoprotein L OS=Heterocephalus glaber OX=10181 GN=GW7_16706 PE=4 SV=1
**G5BGJ6**	Contactin-1 OS=Heterocephalus glaber OX=10181 GN=GW7_19026 PE=4 SV=1
**G5AL32**	V-type proton ATPase catalytic subunit A OS=Heterocephalus glaber OX=10181 GN=GW7_03605 PE=3 SV=1
**G5BUP0**	Transketolase OS=Heterocephalus glaber OX=10181 GN=GW7_01625 PE=4 SV=1
**G5BE32**	Putative pre-mRNA-splicing factor ATP-dependent RNA helicase DHX15 OS=Heterocephalus glaber OX=10181 GN=DHX15 PE=4 SV=1
**G5AZ41**	Rho guanine nucleotide exchange factor 7 OS=Heterocephalus glaber OX=10181 GN=GW7_00283 PE=4 SV=1
**G5B318**	Dihydropyrimidinase-related protein 1 (Fragment) OS=Heterocephalus glaber OX=10181 GN=GW7_14551 PE=4 SV=1
**G5AZ97**	Triple functional domain protein OS=Heterocephalus glaber OX=10181 GN=GW7_01985 PE=4 SV=1
**G5BXH3**	Putative proline--tRNA ligase, mitochondrial OS=Heterocephalus glaber OX=10181 GN=PARS2 PE=4 SV=1
**G5BV89**	Cytochrome b-c1 complex subunit 2, mitochondrial OS=Heterocephalus glaber OX=10181 GN=UQCRC2 PE=3 SV=1
**G5BSI9**	Calcium-transporting ATPase OS=Heterocephalus glaber OX=10181 GN=GW7_13643 PE=3 SV=1
**G5C5M0**	NADH-ubiquinone oxidoreductase 75 kDa subunit, mitochondrial OS=Heterocephalus glaber OX=10181 GN=GW7_17583 PE=3 SV=1
**G5BAX8**	Septin-7 OS=Heterocephalus glaber OX=10181 GN=GW7_00168 PE=3 SV=1
**G5BGB3**	Cullin-associated NEDD8-dissociated protein 1 OS=Heterocephalus glaber OX=10181 GN=CAND1 PE=4 SV=1
**G5CAP7**	Glyceraldehyde-3-phosphate dehydrogenase (Fragment) OS=Heterocephalus glaber OX=10181 GN=GW7_12106 PE=3 SV=1
**G5ATM3**	ERC protein 2 OS=Heterocephalus glaber OX=10181 GN=GW7_00199 PE=4 SV=1
**G5B7T4**	Kinesin heavy chain isoform 5C OS=Heterocephalus glaber OX=10181 GN=GW7_17996 PE=3 SV=1
**G5BKE5**	Tubulin beta chain OS=Heterocephalus glaber OX=10181 GN=GW7_11747 PE=3 SV=1
**G5AZF9**	Ubiquitin-like modifier-activating enzyme 1 OS=Heterocephalus glaber OX=10181 GN=GW7_11282 PE=3 SV=1
**G5C0R6**	Dipeptidyl peptidase 9 OS=Heterocephalus glaber OX=10181 GN=GW7_20503 PE=3 SV=1
**G5ANV4**	Golgin subfamily A member 3 OS=Heterocephalus glaber OX=10181 GN=GW7_09569 PE=4 SV=1
**G5ALS1**	Keratin, type II cytoskeletal 6B OS=Heterocephalus glaber OX=10181 GN=GW7_03778 PE=3 SV=1
**G5AXZ3**	2-oxoglutarate dehydrogenase E1 component-like, mitochondrial OS=Heterocephalus glaber OX=10181 GN=GW7_10398 PE=4 SV=1
**G5BY30**	Aminopeptidase OS=Heterocephalus glaber OX=10181 GN=GW7_00454 PE=3 SV=1
**G5BEX6**	Neural cell adhesion molecule 1 OS=Heterocephalus glaber OX=10181 GN=GW7_02493 PE=4 SV=1
**G5B1L2**	Band 4.1-like protein 3 OS=Heterocephalus glaber OX=10181 GN=GW7_17835 PE=4 SV=1

**G5BU67**	ADP/ATP translocase 2 OS=Heterocephalus glaber OX=10181 GN=GW7_07398 PE=3 SV=1
**G5C4F1**	Tubulin beta chain OS=Heterocephalus glaber OX=10181 GN=GW7_15271 PE=3 SV=1
**G5ARE5**	Vacuolar proton pump subunit B OS=Heterocephalus glaber OX=10181 GN=GW7_01073 PE=3 SV=1
**G5BTE1**	AP-2 complex subunit mu-1 OS=Heterocephalus glaber OX=10181 GN=GW7_17237 PE=3 SV=1
**G5ALS3**	Keratin, type II cytoskeletal 5 OS=Heterocephalus glaber OX=10181 GN=GW7_03780 PE=3 SV=1
**G5AW57**	Aspartate aminotransferase OS=Heterocephalus glaber OX=10181 GN=GOT2 PE=4 SV=1
**G5BJQ1**	Septin-11 OS=Heterocephalus glaber OX=10181 GN=GW7_19874 PE=3 SV=1
**G5BEG2**	L-lactate dehydrogenase OS=Heterocephalus glaber OX=10181 GN=GW7_05659 PE=2 SV=1
**G5BM52**	Creatine kinase U-type, mitochondrial OS=Heterocephalus glaber OX=10181 GN=GW7_18774 PE=3 SV=1
**G5B139**	Amino acid transporter OS=Heterocephalus glaber OX=10181 GN=GW7_01386 PE=3 SV=1
**G5AMR7**	Protein ERGIC-53 OS=Heterocephalus glaber OX=10181 GN=GW7_20044 PE=4 SV=1
**G5C9J0**	Synaptotagmin-1 OS=Heterocephalus glaber OX=10181 GN=GW7_11734 PE=4 SV=1
**G5C4U7**	Aspartate aminotransferase OS=Heterocephalus glaber OX=10181 GN=GOT1 PE=4 SV=1
**G5BX71**	Calcium-transporting ATPase OS=Heterocephalus glaber OX=10181 GN=GW7_19471 PE=3 SV=1
**G5BMD6**	Ankyrin-2 (Fragment) OS=Heterocephalus glaber OX=10181 GN=GW7_01307 PE=4 SV=1
**G5B360**	Calcium-transporting ATPase OS=Heterocephalus glaber OX=10181 GN=GW7_12839 PE=3 SV=1
**G5C6E3**	Neurochondrin (Fragment) OS=Heterocephalus glaber OX=10181 GN=GW7_05394 PE=4 SV=1
**G5BKE6**	Tubulin beta chain OS=Heterocephalus glaber OX=10181 GN=GW7_11748 PE=3 SV=1
**G5APX0**	AP complex subunit beta OS=Heterocephalus glaber OX=10181 GN=GW7_05313 PE=3 SV=1
**G5B577**	Acylglycerol kinase, mitochondrial OS=Heterocephalus glaber OX=10181 GN=GW7_11853 PE=4 SV=1
**G5BU71**	Septin-6 OS=Heterocephalus glaber OX=10181 GN=GW7_07402 PE=3 SV=1
**G5B3C5**	Serine/threonine-protein phosphatase 2A 65 kDa regulatory subunit A alpha isoform OS=Heterocephalus glaber OX=10181 GN=GW7_06227 PE=4 SV=1
**G5ASD2**	Leucine-rich repeat-containing protein 8A OS=Heterocephalus glaber OX=10181 GN=LRRC8A PE=4 SV=1
**G5BI16**	Putative heat shock protein HSP 90-beta-3 OS=Heterocephalus glaber OX=10181 GN=GW7_02247 PE=4 SV=1
**G5BXQ6**	Neurofascin OS=Heterocephalus glaber OX=10181 GN=GW7_15814 PE=4 SV=1
**G5AKA3**	L-lactate dehydrogenase OS=Heterocephalus glaber OX=10181 GN=GW7_11628 PE=2 SV=1
**G5BIL1**	2-oxoglutarate dehydrogenase E1 component, mitochondrial OS=Heterocephalus glaber OX=10181 GN=GW7_08272 PE=4 SV=1
**G5BBZ6**	Guanine nucleotide-binding protein G(I)/G(S)/G(T) subunit beta-2 OS=Heterocephalus glaber OX=10181 GN=GNB2 PE=4 SV=1
**G5BWB9**	ATP-dependent 6-phosphofructokinase OS=Heterocephalus glaber OX=10181 GN=GW7_07004 PE=3 SV=1
**G5CAA4**	Isocitrate dehydrogenase [NADP] OS=Heterocephalus glaber OX=10181 GN=GW7_08829 PE=3 SV=1
**G5BN09**	Voltage-dependent anion-selective channel protein 1 OS=Heterocephalus glaber OX=10181 GN=VDAC1 PE=4 SV=1
**G5BT87**	Histidine-rich glycoprotein OS=Heterocephalus glaber OX=10181 GN=GW7_17202 PE=4 SV=1
**G5BG62**	Malate dehydrogenase OS=Heterocephalus glaber OX=10181 GN=GW7_08880 PE=3 SV=1
**G5B6T3**	ATP-dependent 6-phosphofructokinase OS=Heterocephalus glaber OX=10181 GN=GW7_21506 PE=3 SV=1
**G5BA48**	Stress-70 protein, mitochondrial OS=Heterocephalus glaber OX=10181 GN=GW7_10091 PE=3 SV=1
**G5AMM5**	LanC-like protein 2 OS=Heterocephalus glaber OX=10181 GN=GW7_07012 PE=4 SV=1
**G5C1Q0**	Splicing factor 3B subunit 3 OS=Heterocephalus glaber OX=10181 GN=GW7_01482 PE=4 SV=1
**G5BSN0**	Dedicator of cytokinesis protein 8 OS=Heterocephalus glaber OX=10181 GN=GW7_01505 PE=3 SV=1
**G5AKY4**	Heat shock 70 kDa protein 4L OS=Heterocephalus glaber OX=10181 GN=GW7_10899 PE=3 SV=1
**G5BJY3**	Succinate-semialdehyde dehydrogenase (Fragment) OS=Heterocephalus glaber OX=10181 GN=GW7_06350 PE=3 SV=1
**G5AZ30**	Endoplasmic reticulum resident protein 44 OS=Heterocephalus glaber OX=10181 GN=ERP44 PE=4 SV=1
**G5BN06**	Heat shock 70 kDa protein 4 OS=Heterocephalus glaber OX=10181 GN=HSPA4 PE=3 SV=1
**G5AKB7**	Anion exchange protein OS=Heterocephalus glaber OX=10181 GN=GW7_07691 PE=3 SV=1

**G5AVJ8**	Nck-associated protein 1 (Fragment) OS=Heterocephalus glaber OX=10181 GN=GW7_12399 PE=4 SV=1
**G5BS33**	Hemoglobin subunit beta OS=Heterocephalus glaber OX=10181 GN=GW7_03824 PE=3 SV=1
**G5ATH6**	Guanine nucleotide-binding protein G(I)/G(S)/G(T) subunit beta-1 OS=Heterocephalus glaber OX=10181 GN=GNB1 PE=4 SV=1
**G5B212**	Microtubule-associated protein 1B OS=Heterocephalus glaber OX=10181 GN=GW7_19015 PE=4 SV=1
**G5B4D4**	Ras-related protein Rab-3A OS=Heterocephalus glaber OX=10181 GN=RAB3A PE=4 SV=1
**G5BW99**	Mitochondrial 2-oxoglutarate/malate carrier protein isoform 2 OS=Heterocephalus glaber OX=10181 GN=SLC25A11 PE=3 SV=1
**G5BI79**	Fascin OS=Heterocephalus glaber OX=10181 GN=GW7_16884 PE=4 SV=1
**G5B2W9**	ADP/ATP translocase 1 OS=Heterocephalus glaber OX=10181 GN=GW7_12487 PE=3 SV=1
**G5B0M6**	Keratin, type I cytoskeletal 14 OS=Heterocephalus glaber OX=10181 GN=GW7_06879 PE=3 SV=1
**G5C2C6**	Dihydrolipoyl dehydrogenase OS=Heterocephalus glaber OX=10181 GN=DLD PE=3 SV=1
**G5B4X0**	Calcium-transporting ATPase OS=Heterocephalus glaber OX=10181 GN=GW7_06050 PE=3 SV=1
**G5APJ0**	Synapsin-2 OS=Heterocephalus glaber OX=10181 GN=GW7_10966 PE=4 SV=1
**G5CAS2**	Prohibitin-2 OS=Heterocephalus glaber OX=10181 GN=GW7_12131 PE=4 SV=1
**G5BHP2**	E3 ubiquitin-protein ligase CBL (Fragment) OS=Heterocephalus glaber OX=10181 GN=GW7_18686 PE=4 SV=1
**G5B6D0**	Glutamine synthetase OS=Heterocephalus glaber OX=10181 GN=GW7_08254 PE=3 SV=1
**G5C5Z3**	Ras-related protein Rab-1A (Fragment) OS=Heterocephalus glaber OX=10181 GN=GW7_18354 PE=4 SV=1
**G5CAZ8**	Acetyltransferase component of pyruvate dehydrogenase complex OS=Heterocephalus glaber OX=10181 GN=DLAT PE=3 SV=1
**G5BKX2**	Cytoplasmic FMR1-interacting protein 2 OS=Heterocephalus glaber OX=10181 GN=GW7_12158 PE=4 SV=1
**G5B6V7**	78 kDa glucose-regulated protein OS=Heterocephalus glaber OX=10181 GN=HSPA5 PE=3 SV=1
**G5AP69**	Serine/threonine-protein phosphatase (Fragment) OS=Heterocephalus glaber OX=10181 GN=GW7_10360 PE=3 SV=1
**G5BBX1**	Calcium-dependent secretion activator 1 (Fragment) OS=Heterocephalus glaber OX=10181 GN=GW7_02265 PE=4 SV=1
**G5BMR4**	Guanine nucleotide-binding protein G(O) subunit alpha (Fragment) OS=Heterocephalus glaber OX=10181 GN=GW7_09344 PE=4 SV=1
**G5BXL7**	Glucose-6-phosphate isomerase OS=Heterocephalus glaber OX=10181 GN=GW7_18400 PE=3 SV=1
**G5BH24**	Synaptic vesicle glycoprotein 2A OS=Heterocephalus glaber OX=10181 GN=SV2A PE=4 SV=1
**G5BYJ8**	Hemoglobin subunit beta OS=Heterocephalus glaber OX=10181 GN=GW7_14163 PE=3 SV=1
**G5AXS2**	Ras-related protein Rab-14 OS=Heterocephalus glaber OX=10181 GN=RAB14 PE=4 SV=1
**G5AZH3**	Cytoplasmic FMR1-interacting protein 1 (Fragment) OS=Heterocephalus glaber OX=10181 GN=GW7_18826 PE=4 SV=1
**G5C7M9**	Dynamin-3 (Fragment) OS=Heterocephalus glaber OX=10181 GN=GW7_06265 PE=3 SV=1
**G5BA56**	1,4-alpha-glucan-branching enzyme OS=Heterocephalus glaber OX=10181 GN=GW7_07702 PE=4 SV=1
**G5BPM1**	Alpha-2-macroglobulin OS=Heterocephalus glaber OX=10181 GN=GW7_17301 PE=4 SV=1
**G5B1C5**	T-complex protein 1 subunit theta OS=Heterocephalus glaber OX=10181 GN=GW7_13214 PE=3 SV=1
**G5BVL3**	14-3-3 protein epsilon OS=Heterocephalus glaber OX=10181 GN=GW7_08232 PE=3 SV=1
**G5C0K7**	Myelin basic protein OS=Heterocephalus glaber OX=10181 GN=GW7_03536 PE=4 SV=1
**G5C3N6**	Vacuolar protein sorting-associated protein 35 OS=Heterocephalus glaber OX=10181 GN=GW7_08700 PE=3 SV=1
**G5BI06**	Basement membrane-specific heparan sulfate proteoglycan core protein OS=Heterocephalus glaber OX=10181 GN=GW7_07516 PE=4 SV=1
**G5CA17**	Septin-5 (Fragment) OS=Heterocephalus glaber OX=10181 GN=GW7_11135 PE=3 SV=1
**G5C3C3**	Tubulin beta chain OS=Heterocephalus glaber OX=10181 GN=GW7_04753 PE=3 SV=1
**G5BYD1**	Syntaxin-1B OS=Heterocephalus glaber OX=10181 GN=GW7_05131 PE=3 SV=1
**G5CAR8**	Gamma-enolase OS=Heterocephalus glaber OX=10181 GN=GW7_12127 PE=2 SV=1
**G5BYB7**	UPF0420 protein C16orf58 OS=Heterocephalus glaber OX=10181 GN=GW7_05117 PE=4 SV=1
**G5AXH0**	Actin, gamma-enteric smooth muscle OS=Heterocephalus glaber OX=10181 GN=GW7_02888 PE=3 SV=1

**G5BSN2**	Phosphoglucomutase-1 OS=Heterocephalus glaber OX=10181 GN=GW7_01507 PE=4 SV=1
**G5AR38**	Endoplasmin (Fragment) OS=Heterocephalus glaber OX=10181 GN=GW7_10317 PE=3 SV=1
**G5AP01**	14-3-3 protein zeta/delta OS=Heterocephalus glaber OX=10181 GN=GW7_00766 PE=3 SV=1
**G5B2A3**	Protein kinase C-binding protein NELL2 OS=Heterocephalus glaber OX=10181 GN=GW7_03965 PE=4 SV=1
**G5BUH8**	Dynamin-2 OS=Heterocephalus glaber OX=10181 GN=GW7_13280 PE=3 SV=1
**G5BKG1**	Mitogen-activated protein kinase OS=Heterocephalus glaber OX=10181 GN=GW7_15351 PE=4 SV=1
**G5B2K8**	Phosphate carrier protein, mitochondrial OS=Heterocephalus glaber OX=10181 GN=GW7_08592 PE=3 SV=1
**G5C8D3**	Elongation factor 1-alpha (Fragment) OS=Heterocephalus glaber OX=10181 GN=GW7_09733 PE=3 SV=1
**G5BSI1**	Dynamin-1-like protein OS=Heterocephalus glaber OX=10181 GN=GW7_21637 PE=3 SV=1
**G5AVL7**	Cytochrome b-c1 complex subunit 1, mitochondrial OS=Heterocephalus glaber OX=10181 GN=UQCRC1 PE=4 SV=1
**G5C7R8**	Constitutive coactivator of peroxisome proliferator-activated receptor gamma OS=Heterocephalus glaber OX=10181 GN=GW7_11305 PE=4 SV=1
**G5BFK9**	Rab GDP dissociation inhibitor OS=Heterocephalus glaber OX=10181 GN=GW7_19328 PE=3 SV=1
**G5C1L8**	Dihydrolipoyllysine-residue succinyltransferase component of 2-oxoglutarate dehydrogenase complex, mitochondrial OS=Heterocephalus glaber OX=10181 GN=DLST PE=4 SV=1
**G5C530**	6-phosphogluconate dehydrogenase, decarboxylating OS=Heterocephalus glaber OX=10181 GN=GW7_09120 PE=3 SV=1
**G5BWX1**	Ras-related protein Rab-1B OS=Heterocephalus glaber OX=10181 GN=RAB1B PE=4 SV=1
**G5AXV6**	ATP synthase subunit gamma OS=Heterocephalus glaber OX=10181 GN=GW7_14036 PE=3 SV=1
**G5BBV6**	14-3-3 protein eta OS=Heterocephalus glaber OX=10181 GN=GW7_17673 PE=3 SV=1
**G5BTW5**	NADH dehydrogenase [ubiquinone] flavoprotein 1, mitochondrial OS=Heterocephalus glaber OX=10181 GN=NDUFV1 PE=3 SV=1
**G5B0J8**	V-type proton ATPase subunit a OS=Heterocephalus glaber OX=10181 GN=GW7_06851 PE=3 SV=1
**G5AU37**	ADP-ribosylation factor 3 OS=Heterocephalus glaber OX=10181 GN=GW7_14469 PE=3 SV=1
**G5C1Y4**	T-complex protein 1 subunit delta OS=Heterocephalus glaber OX=10181 GN=GW7_01322 PE=3 SV=1
**G5B251**	Heat shock cognate 71 kDa protein OS=Heterocephalus glaber OX=10181 GN=GW7_09579 PE=3 SV=1
**G5B2V7**	60 kDa heat shock protein, mitochondrial OS=Heterocephalus glaber OX=10181 GN=GW7_17339 PE=4 SV=1
**G5BKW4**	Paraspeckle component 1 OS=Heterocephalus glaber OX=10181 GN=GW7_03914 PE=4 SV=1
**G5BE96**	Synaptojanin-1 (Fragment) OS=Heterocephalus glaber OX=10181 GN=GW7_08747 PE=4 SV=1
**G5C9G0**	Amino acid transporter OS=Heterocephalus glaber OX=10181 GN=GW7_08002 PE=3 SV=1
**G5ALK7**	Elongation factor 1-alpha OS=Heterocephalus glaber OX=10181 GN=GW7_12808 PE=3 SV=1
**G5C8G6**	Mitochondrial glutamate carrier 1 OS=Heterocephalus glaber OX=10181 GN=GW7_12706 PE=3 SV=1
**G5B6P0**	Alpha-1,4 glucan phosphorylase OS=Heterocephalus glaber OX=10181 GN=GW7_20662 PE=3 SV=1
**G5ASS3**	T-complex protein 1 subunit zeta isoform a OS=Heterocephalus glaber OX=10181 GN=CCT6A PE=3 SV=1
**G5BAY8**	Lysine-specific demethylase 5C OS=Heterocephalus glaber OX=10181 GN=GW7_04947 PE=4 SV=1
**G5C8M3**	Malate dehydrogenase OS=Heterocephalus glaber OX=10181 GN=GW7_04884 PE=3 SV=1
**G5B710**	T-complex protein 1 subunit epsilon OS=Heterocephalus glaber OX=10181 GN=GW7_03792 PE=3 SV=1
**G5BZX7**	CaM kinase-like vesicle-associated protein OS=Heterocephalus glaber OX=10181 GN=GW7_19932 PE=4 SV=1
**G5BWS7**	AP complex subunit beta OS=Heterocephalus glaber OX=10181 GN=GW7_21525 PE=3 SV=1
**G5C2C4**	Neuronal cell adhesion molecule (Fragment) OS=Heterocephalus glaber OX=10181 GN=GW7_15852 PE=4 SV=1
**G5BF93**	Trimethyllysine dioxygenase, mitochondrial OS=Heterocephalus glaber OX=10181 GN=GW7_10776 PE=4 SV=1

**G5BZ40**	Pyruvate dehydrogenase E1 component subunit alpha OS=Heterocephalus glaber OX=10181 GN=GW7_01970 PE=4 SV=1
**G5B840**	Band 4.1-like protein 1 OS=Heterocephalus glaber OX=10181 GN=GW7_21265 PE=4 SV=1
**G5CBK7**	Trifunctional enzyme subunit alpha, mitochondrial OS=Heterocephalus glaber OX=10181 GN=HADHA PE=3 SV=1
**G5CAR5**	Triosephosphate isomerase OS=Heterocephalus glaber OX=10181 GN=GW7_12124 PE=3 SV=1
**G5B615**	V-type proton ATPase subunit H OS=Heterocephalus glaber OX=10181 GN=ATP6V1H PE=3 SV=1
**G5C8M4**	UTP--glucose-1-phosphate uridylyltransferase OS=Heterocephalus glaber OX=10181 GN=GW7_04885 PE=3 SV=1
**G5C7U4**	T-complex protein 1 subunit beta OS=Heterocephalus glaber OX=10181 GN=GW7_11343 PE=3 SV=1
**G5BXY1**	Hemoglobin subunit alpha OS=Heterocephalus glaber OX=10181 GN=HBA2 PE=3 SV=1
**G5BHY3**	PITH domain-containing protein (Fragment) OS=Heterocephalus glaber OX=10181 GN=GW7_07493 PE=4 SV=1
**G5AR92**	T-complex protein 1 subunit gamma (Fragment) OS=Heterocephalus glaber OX=10181 GN=GW7_14281 PE=3 SV=1
**G5BYW2**	Phenylalanyl-tRNA synthetase alpha chain OS=Heterocephalus glaber OX=10181 GN=GW7_16082 PE=4 SV=1
**G5C7J1**	Voltage-dependent anion-selective channel protein 2 OS=Heterocephalus glaber OX=10181 GN=GW7_13179 PE=4 SV=1
**G5BQA9**	Serotransferrin OS=Heterocephalus glaber OX=10181 GN=GW7_05106 PE=3 SV=1
**G5B0L4**	ATP-citrate synthase OS=Heterocephalus glaber OX=10181 GN=GW7_06867 PE=3 SV=1
**G5AKS9**	Nitric oxide synthase, brain OS=Heterocephalus glaber OX=10181 GN=GW7_10175 PE=4 SV=1
**G5B0N0**	Keratin, type I cytoskeletal 13 OS=Heterocephalus glaber OX=10181 GN=GW7_06883 PE=3 SV=1
**G5BEC9**	Kinesin heavy chain isoform 5A OS=Heterocephalus glaber OX=10181 GN=KIF5A PE=3 SV=1
**G5ALF6**	Phosphoglycerate kinase OS=Heterocephalus glaber OX=10181 GN=PGK1 PE=3 SV=1
**G5C773**	Band 4.1-like protein 2 OS=Heterocephalus glaber OX=10181 GN=GW7_21858 PE=4 SV=1
**G5B6W0**	Protein SCAI OS=Heterocephalus glaber OX=10181 GN=GW7_01139 PE=4 SV=1
**G5BLU3**	DnaJ-like protein subfamily C member 10 (Fragment) OS=Heterocephalus glaber OX=10181 GN=GW7_10932 PE=4 SV=1
**G5B382**	Synaptotagmin-2 OS=Heterocephalus glaber OX=10181 GN=GW7_12861 PE=4 SV=1
**G5BQX0**	AMP deaminase OS=Heterocephalus glaber OX=10181 GN=GW7_20134 PE=3 SV=1
**G5AYH2**	Elongation factor 1-gamma OS=Heterocephalus glaber OX=10181 GN=GW7_01766 PE=4 SV=1
**G5BZY6**	Guanine nucleotide-binding protein G(I), alpha-2 subunit OS=Heterocephalus glaber OX=10181 GN=GW7_19941 PE=4 SV=1
**G5B928**	Calcium-transporting ATPase OS=Heterocephalus glaber OX=10181 GN=GW7_14600 PE=3 SV=1
**G5BEK1**	14-3-3 protein theta OS=Heterocephalus glaber OX=10181 GN=YWHAQ PE=3 SV=1
**G5CBJ9**	Dihydropyrimidinase-related protein 5 OS=Heterocephalus glaber OX=10181 GN=GW7_17031 PE=4 SV=1
**G5BFL5**	Glucose-6-phosphate 1-dehydrogenase OS=Heterocephalus glaber OX=10181 GN=GW7_19334 PE=3 SV=1
**G5BQU1**	Neural cell adhesion molecule L1 (Fragment) OS=Heterocephalus glaber OX=10181 GN=GW7_02026 PE=4 SV=1
**G5BSD0**	4-aminobutyrate aminotransferase, mitochondrial (Fragment) OS=Heterocephalus glaber OX=10181 GN=GW7_08976 PE=3 SV=1
**G5B2Y7**	WD repeat-containing protein 1 OS=Heterocephalus glaber OX=10181 GN=GW7_14537 PE=4 SV=1
**G5B6J0**	Cofilin-1 OS=Heterocephalus glaber OX=10181 GN=GW7_20612 PE=3 SV=1
**G5BCQ6**	Transcriptional activator protein Pur-alpha OS=Heterocephalus glaber OX=10181 GN=GW7_08872 PE=4 SV=1
**G5C3R7**	Septin-2 OS=Heterocephalus glaber OX=10181 GN=GW7_11070 PE=3 SV=1
**G5CAX4**	Heat shock cognate 71 kDa protein OS=Heterocephalus glaber OX=10181 GN=GW7_21369 PE=3 SV=1
**G5C7W9**	Alpha-actinin-1 OS=Heterocephalus glaber OX=10181 GN=GW7_03701 PE=4 SV=1
**G5ALX2**	Catenin beta-1 OS=Heterocephalus glaber OX=10181 GN=GW7_09171 PE=4 SV=1
**G5ARZ9**	NADH dehydrogenase (Ubiquinone) 1 alpha subcomplex subunit 9, mitochondrial OS=Heterocephalus glaber OX=10181 GN=NDUFA9 PE=4 SV=1
**G5BQX2**	Glutathione S-transferase OS=Heterocephalus glaber OX=10181 GN=GW7_20136 PE=3 SV=1

**G5BXR0**	Acetyl-CoA acetyltransferase, mitochondrial OS=Heterocephalus glaber OX=10181 GN=GW7_19747 PE=3 SV=1
**G5AKU7**	Aldehyde dehydrogenase, mitochondrial isoform 1 OS=Heterocephalus glaber OX=10181 GN=ALDH2 PE=3 SV=1
**G5B4Q7**	Arf-GAP with SH3 domain, ANK repeat and PH domain-containing protein 1 OS=Heterocephalus glaber OX=10181 GN=GW7_10926 PE=4 SV=1
**G5C873**	Neural cell adhesion molecule 2 OS=Heterocephalus glaber OX=10181 GN=GW7_07725 PE=4 SV=1
**G5BJ37**	Keratin, type II cytoskeletal 79 OS=Heterocephalus glaber OX=10181 GN=GW7_10814 PE=3 SV=1
**G5BBP4**	EF-hand calcium-binding protein 1 OS=Heterocephalus glaber OX=10181 GN=GW7_09364 PE=4 SV=1
**G5BG59**	14-3-3 protein gamma (Fragment) OS=Heterocephalus glaber OX=10181 GN=GW7_08877 PE=3 SV=1
**G5B4U5**	Uncharacterized protein OS=Heterocephalus glaber OX=10181 GN=GW7_03212 PE=4 SV=1
**G5BRD7**	Sarcoplasmic/endoplasmic reticulum calcium ATPase 1 OS=Heterocephalus glaber OX=10181 GN=GW7_14894 PE=3 SV=1
**G5BET5**	ATP-dependent RNA helicase DDX1 OS=Heterocephalus glaber OX=10181 GN=GW7_19785 PE=4 SV=1
**G5BPG8**	Succinate dehydrogenase [ubiquinone] flavoprotein subunit, mitochondrial OS=Heterocephalus glaber OX=10181 GN=GW7_14707 PE=3 SV=1
**G5BCH1**	Alpha-adducin OS=Heterocephalus glaber OX=10181 GN=ADD1 PE=4 SV=1
**G5BLD9**	Myelin proteolipid protein (Fragment) OS=Heterocephalus glaber OX=10181 GN=GW7_06253 PE=4 SV=1
**G5BHA8**	ADP-ribosylation factor 5 OS=Heterocephalus glaber OX=10181 GN=GW7_16812 PE=3 SV=1
**G5AQD6**	3,2-trans-enoyl-CoA isomerase, mitochondrial OS=Heterocephalus glaber OX=10181 GN=GW7_05021 PE=3 SV=1
**G5BHI4**	NADH dehydrogenase [ubiquinone] 1 alpha subcomplex subunit 10, mitochondrial OS=Heterocephalus glaber OX=10181 GN=GW7_08787 PE=3 SV=1
**G5BQX5**	Glutathione S-transferase OS=Heterocephalus glaber OX=10181 GN=GW7_20139 PE=3 SV=1
**G5C9N9**	Myotubularin (Fragment) OS=Heterocephalus glaber OX=10181 GN=GW7_05283 PE=4 SV=1
**G5BB78**	Citrate synthase (Fragment) OS=Heterocephalus glaber OX=10181 GN=GW7_15757 PE=3 SV=1
**G5AMA2**	NAD-dependent protein deacetylase OS=Heterocephalus glaber OX=10181 GN=GW7_16704 PE=3 SV=1
**G5BGY3**	Phenylalanine--tRNA ligase beta subunit OS=Heterocephalus glaber OX=10181 GN=FARSB PE=4 SV=1
**G5BLR5**	Fructose-bisphosphate aldolase A OS=Heterocephalus glaber OX=10181 GN=GW7_10395 PE=4 SV=1
**G5BBE2**	Glutamate--cysteine ligase catalytic subunit (Fragment) OS=Heterocephalus glaber OX=10181 GN=GW7_06303 PE=4 SV=1
**G5BRK8**	Importin-7 OS=Heterocephalus glaber OX=10181 GN=GW7_21205 PE=4 SV=1
**G5APK7**	Zinc transporter ZIP10 OS=Heterocephalus glaber OX=10181 GN=GW7_00937 PE=4 SV=1
**G5BDV3**	Rap1 GTPase-GDP dissociation stimulator 1 (Fragment) OS=Heterocephalus glaber OX=10181 GN=GW7_10113 PE=4 SV=1
**G5B5F3**	Vesicle-associated membrane protein 2 (Fragment) OS=Heterocephalus glaber OX=10181 GN=GW7_13855 PE=4 SV=1
**G5CBK9**	Ras-related protein Rab-10 OS=Heterocephalus glaber OX=10181 GN=GW7_17041 PE=4 SV=1
**G5C5T9**	Cytochrome c1, heme protein, mitochondrial OS=Heterocephalus glaber OX=10181 GN=GW7_19247 PE=4 SV=1
**G5B0M4**	Keratin, type I cytoskeletal 17 OS=Heterocephalus glaber OX=10181 GN=GW7_06877 PE=3 SV=1
**G5BPR0**	Neuronal-specific septin-3 (Fragment) OS=Heterocephalus glaber OX=10181 GN=GW7_19448 PE=3 SV=1
**G5AM45**	Zinc transporter 1 OS=Heterocephalus glaber OX=10181 GN=GW7_16306 PE=4 SV=1
**G5AXT3**	Adenylyl cyclase-associated protein OS=Heterocephalus glaber OX=10181 GN=GW7_06940 PE=3 SV=1
**G5C0C5**	Phosphoglycerate mutase OS=Heterocephalus glaber OX=10181 GN=GW7_09237 PE=3 SV=1
**G5AKJ4**	Aspartyl aminopeptidase OS=Heterocephalus glaber OX=10181 GN=DNPEP PE=3 SV=1
**G5BWW8**	Splicing factor 3B subunit 2 OS=Heterocephalus glaber OX=10181 GN=GW7_07186 PE=4 SV=1
**G5AMB0**	Alpha-actinin-4 OS=Heterocephalus glaber OX=10181 GN=GW7_16712 PE=4 SV=1

**G5BCG5**	Metabotropic glutamate receptor 4 OS=Heterocephalus glaber OX=10181 GN=GW7_15655 PE=3 SV=1
**G5C8P9**	Lupus La protein OS=Heterocephalus glaber OX=10181 GN=SSB PE=4 SV=1
**G5CAF3**	Sideroflexin-3 OS=Heterocephalus glaber OX=10181 GN=GW7_15882 PE=4 SV=1
**G5ATY3**	Anion exchange protein OS=Heterocephalus glaber OX=10181 GN=GW7_14415 PE=3 SV=1
**G5B2P2**	Heat shock protein 105 kDa OS=Heterocephalus glaber OX=10181 GN=GW7_02038 PE=3 SV=1
**G5AKF3**	Myelin-oligodendrocyte glycoprotein (Fragment) OS=Heterocephalus glaber OX=10181 GN=GW7_10279 PE=4 SV=1
**G5BCR1**	NAD(P) transhydrogenase, mitochondrial OS=Heterocephalus glaber OX=10181 GN=GW7_04058 PE=4 SV=1
**G5BB30**	14-3-3 protein beta/alpha OS=Heterocephalus glaber OX=10181 GN=YWHAB PE=3 SV=1
**G5ASQ2**	Septin-8 OS=Heterocephalus glaber OX=10181 GN=GW7_12246 PE=3 SV=1
**G5BUK7**	Pyruvate dehydrogenase E1 component subunit beta OS=Heterocephalus glaber OX=10181 GN=PDHB PE=4 SV=1
**G5BM50**	Microtubule-associated protein 1A OS=Heterocephalus glaber OX=10181 GN=GW7_18772 PE=4 SV=1
**G5B7R5**	Formin-like protein 2 (Fragment) OS=Heterocephalus glaber OX=10181 GN=GW7_17977 PE=4 SV=1
**G5ASH7**	Guanine deaminase OS=Heterocephalus glaber OX=10181 GN=GW7_07953 PE=3 SV=1
**G5AM26**	ATP synthase subunit O, mitochondrial OS=Heterocephalus glaber OX=10181 GN=GW7_16287 PE=3 SV=1
**G5AQU8**	Ubiquitin-conjugating enzyme E2 O OS=Heterocephalus glaber OX=10181 GN=GW7_06422 PE=4 SV=1
**G5BRF2**	Neuroplastin OS=Heterocephalus glaber OX=10181 GN=GW7_19526 PE=4 SV=1
**G5B3I7**	Ras-related protein Rab-5A OS=Heterocephalus glaber OX=10181 GN=RAB5A PE=4 SV=1
**G5C5Q2**	ELKS/RAB6-interacting/CAST family member 1 OS=Heterocephalus glaber OX=10181 GN=GW7_19210 PE=4 SV=1
**G5AWZ4**	Coronin OS=Heterocephalus glaber OX=10181 GN=GW7_02143 PE=3 SV=1
**G5AKP8**	Phosphatidylinositol-4-phosphate 5-kinase type-1 gamma (Fragment) OS=Heterocephalus glaber OX=10181 GN=GW7_11309 PE=4 SV=1
**G5BJ39**	Keratin, type II cytoskeletal 8 OS=Heterocephalus glaber OX=10181 GN=GW7_10816 PE=2 SV=1
**G5BZB8**	Elongation factor 2 OS=Heterocephalus glaber OX=10181 GN=EEF2 PE=4 SV=1
**G5BWI0**	NADH dehydrogenase (Ubiquinone) iron-sulfur protein 2, mitochondrial isoform 1 OS=Heterocephalus glaber OX=10181 GN=NDUFS2 PE=3 SV=1
**G5AXG2**	Dynactin subunit 1 OS=Heterocephalus glaber OX=10181 GN=GW7_02880 PE=4 SV=1
**G5C394**	Calcium/calmodulin-dependent protein kinase type II alpha chain OS=Heterocephalus glaber OX=10181 GN=GW7_00118 PE=4 SV=1
**G5B558**	Putative RNA-binding protein Luc7-like 2 (Fragment) OS=Heterocephalus glaber OX=10181 GN=GW7_11834 PE=4 SV=1
**G5ALU1**	Regulating synaptic membrane exocytosis protein 1 OS=Heterocephalus glaber OX=10181 GN=GW7_17598 PE=4 SV=1
**G5AWA9**	Ras-related protein Rab-8B OS=Heterocephalus glaber OX=10181 GN=GW7_00144 PE=4 SV=1
**G5BDD6**	Small conductance calcium-activated potassium channel protein 3 OS=Heterocephalus glaber OX=10181 GN=GW7_05186 PE=4 SV=1
**G5BY29**	Importin subunit beta-1 OS=Heterocephalus glaber OX=10181 GN=GW7_00453 PE=4 SV=1
**G5BJ97**	Fatty acid synthase OS=Heterocephalus glaber OX=10181 GN=GW7_04112 PE=4 SV=1
**G5BGK6**	Glutaminase kidney isoform, mitochondrial (Fragment) OS=Heterocephalus glaber OX=10181 GN=GW7_01412 PE=3 SV=1
**G5B521**	Malic enzyme (Fragment) OS=Heterocephalus glaber OX=10181 GN=GW7_17644 PE=3 SV=1
**G5CBQ4**	T-complex protein 1 subunit eta OS=Heterocephalus glaber OX=10181 GN=GW7_03296 PE=3 SV=1
**G5C6C2**	Nucleoprotein TPR OS=Heterocephalus glaber OX=10181 GN=GW7_07103 PE=4 SV=1
**G5BAB4**	Constitutive coactivator of PPAR-gamma-like protein 2 OS=Heterocephalus glaber OX=10181 GN=GW7_01037 PE=4 SV=1
**G5BD70**	Adenosylhomocysteinase OS=Heterocephalus glaber OX=10181 GN=GW7_07805 PE=3 SV=1
**G5AL84**	Leucine-rich PPR motif-containing protein, mitochondrial (Fragment) OS=Heterocephalus glaber OX=10181 GN=GW7_03995 PE=4 SV=1
**G5BJQ3**	ADP-ribosylation factor 3 OS=Heterocephalus glaber OX=10181 GN=GW7_19876 PE=3 SV=1

**G5BN93**	Synaptic vesicle membrane protein VAT-1-like protein OS=Heterocephalus glaber OX=10181 GN=GW7_07847 PE=4 SV=1
**G5BDP5**	Septin-8 OS=Heterocephalus glaber OX=10181 GN=GW7_11423 PE=3 SV=1
**G5B399**	Cysteine and glycine-rich protein 1 isoform 3 OS=Heterocephalus glaber OX=10181 GN=CSRP1 PE=4 SV=1
**G5BC67**	T-complex protein 1 subunit alpha OS=Heterocephalus glaber OX=10181 GN=GW7_11562 PE=3 SV=1
**G5BS07**	Dipeptidyl aminopeptidase-like protein 6 OS=Heterocephalus glaber OX=10181 GN=GW7_03940 PE=3 SV=1
**G5CB51**	Sodium/potassium-transporting ATPase subunit beta OS=Heterocephalus glaber OX=10181 GN=GW7_09541 PE=3 SV=1
**G5C4P1**	Prohibitin OS=Heterocephalus glaber OX=10181 GN=GW7_21759 PE=4 SV=1
**G5BG31**	MICOS complex subunit MIC60 OS=Heterocephalus glaber OX=10181 GN=IMMT PE=3 SV=1
**G5BB94**	Ras-related protein Rab-5B OS=Heterocephalus glaber OX=10181 GN=RAB5B PE=4 SV=1
**G5BWN6**	V-type proton ATPase subunit E 1 isoform b OS=Heterocephalus glaber OX=10181 GN=ATP6V1E1 PE=3 SV=1
**G5AN56**	Very-long-chain (3R)-3-hydroxyacyl-CoA dehydratase (Fragment) OS=Heterocephalus glaber OX=10181 GN=GW7_19668 PE=3 SV=1
**G5ALD6**	D-beta-hydroxybutyrate dehydrogenase, mitochondrial OS=Heterocephalus glaber OX=10181 GN=BDH1 PE=3 SV=1
**G5BCA6**	Dedicator of cytokinesis protein 4 (Fragment) OS=Heterocephalus glaber OX=10181 GN=GW7_16648 PE=3 SV=1
**G5BTQ7**	Succinate--CoA ligase [ADP-forming] subunit beta, mitochondrial OS=Heterocephalus glaber OX=10181 GN=SUCLA2 PE=3 SV=1
**G5B1R3**	AP-3 complex subunit beta OS=Heterocephalus glaber OX=10181 GN=GW7_09432 PE=3 SV=1
**G5BH50**	Profilin (Fragment) OS=Heterocephalus glaber OX=10181 GN=GW7_12907 PE=3 SV=1
**G5CBF1**	Protein kinase C gamma type (Fragment) OS=Heterocephalus glaber OX=10181 GN=GW7_20162 PE=3 SV=1
**G5BXS3**	Ankyrin-3 OS=Heterocephalus glaber OX=10181 GN=GW7_01938 PE=4 SV=1
**G5C3G5**	Heat shock 70 kDa protein 1L OS=Heterocephalus glaber OX=10181 GN=GW7_04795 PE=3 SV=1
**G5APR8**	BTB/POZ domain-containing protein KCTD16 OS=Heterocephalus glaber OX=10181 GN=GW7_01638 PE=4 SV=1
**G5BWU7**	Catenin alpha-2 (Fragment) OS=Heterocephalus glaber OX=10181 GN=GW7_19189 PE=4 SV=1
**G5BG81**	Syntaxin-1A OS=Heterocephalus glaber OX=10181 GN=GW7_08899 PE=3 SV=1
**G5BJK0**	Synaptophysin OS=Heterocephalus glaber OX=10181 GN=GW7_20097 PE=4 SV=1
**G5B8J0**	Noelin (Fragment) OS=Heterocephalus glaber OX=10181 GN=GW7_14332 PE=4 SV=1
**G5B5D5**	Sodium/potassium-transporting ATPase subunit beta OS=Heterocephalus glaber OX=10181 GN=GW7_13837 PE=3 SV=1
**G5BFB4**	Dihydropyrimidinase-related protein 4 OS=Heterocephalus glaber OX=10181 GN=GW7_14070 PE=4 SV=1
**G5B7Q2**	Solute carrier family 12 member 5 OS=Heterocephalus glaber OX=10181 GN=GW7_06548 PE=4 SV=1
**G5BY03**	Neuronal membrane glycoprotein M6-a OS=Heterocephalus glaber OX=10181 GN=GW7_21848 PE=4 SV=1
**G5AQ71**	Kalirin (Fragment) OS=Heterocephalus glaber OX=10181 GN=GW7_10663 PE=4 SV=1
**G5C4L3**	Nucleoside diphosphate kinase (Fragment) OS=Heterocephalus glaber OX=10181 GN=GW7_21798 PE=3 SV=1
**G5C289**	Microtubule-associated protein RP/EB family member 2 (Fragment) OS=Heterocephalus glaber OX=10181 GN=GW7_13100 PE=4 SV=1
**G5APW6**	NipSnap-like protein 1 (Fragment) OS=Heterocephalus glaber OX=10181 GN=GW7_05309 PE=4 SV=1
**G5C2D5**	cAMP-dependent protein kinase type II-beta regulatory subunit OS=Heterocephalus glaber OX=10181 GN=PRKAR2B PE=4 SV=1
**G5ARM9**	Heterogeneous nuclear ribonucleoprotein K OS=Heterocephalus glaber OX=10181 GN=HNRNPK PE=4 SV=1
**G5ARG8**	Heat shock 70 kDa protein 12A OS=Heterocephalus glaber OX=10181 GN=GW7_21295 PE=4 SV=1
**2::tr|G5AZE3|G5AZE3_HETGA**	Receptor-type tyrosine-protein phosphatase zeta OS=Heterocephalus glaber OX=10181 GN=GW7_13190 PE=4 SV=1

**G5AY28**	Synaptotagmin-17 OS=Heterocephalus glaber OX=10181 GN=GW7_06689 PE=4 SV=1
**G5AV02**	Glucocorticoid receptor DNA-binding factor 1 OS=Heterocephalus glaber OX=10181 GN=GW7_18127 PE=4 SV=1
**G5BK64**	Phosphatidylinositol-5-phosphate 4-kinase type-2 beta (Fragment) OS=Heterocephalus glaber OX=10181 GN=GW7_13066 PE=4 SV=1
**G5B8E6**	F-actin-capping protein subunit alpha (Fragment) OS=Heterocephalus glaber OX=10181 GN=GW7_12267 PE=3 SV=1
**G5B0K5**	Ras-related protein Rab-5C OS=Heterocephalus glaber OX=10181 GN=GW7_06858 PE=4 SV=1
**G5BH87**	Adenosylhomocysteinase (Fragment) OS=Heterocephalus glaber OX=10181 GN=GW7_16791 PE=3 SV=1
**G5BL29**	Fumarate hydratase, mitochondrial OS=Heterocephalus glaber OX=10181 GN=FH PE=3 SV=1
**G5BTZ8**	Heterogeneous nuclear ribonucleoprotein A3 OS=Heterocephalus glaber OX=10181 GN=GW7_13685 PE=4 SV=1
**G5BX63**	NADH dehydrogenase (Ubiquinone) 1 beta subcomplex subunit 5, mitochondrial isoform 1 OS=Heterocephalus glaber OX=10181 GN=NDUFB5 PE=4 SV=1
**G5C3T1**	Actin-related protein 3 OS=Heterocephalus glaber OX=10181 GN=GW7_07463 PE=3 SV=1
**G5C4V9**	Ras-related protein Rab-2A OS=Heterocephalus glaber OX=10181 GN=GW7_18515 PE=4 SV=1
**G5BTX3**	14-3-3 protein eta OS=Heterocephalus glaber OX=10181 GN=GW7_17473 PE=3 SV=1
**G5B6T9**	Amphiphysin OS=Heterocephalus glaber OX=10181 GN=GW7_09869 PE=4 SV=1
**G5B3J1**	Rab GDP dissociation inhibitor (Fragment) OS=Heterocephalus glaber OX=10181 GN=GW7_02321 PE=3 SV=1
**G5AS76**	40S ribosomal protein S3 OS=Heterocephalus glaber OX=10181 GN=RPS3 PE=3 SV=1
**G5BLW2**	Glutamate receptor 2 OS=Heterocephalus glaber OX=10181 GN=GW7_04537 PE=3 SV=1
**G5C4J2**	Glyceraldehyde-3-phosphate dehydrogenase OS=Heterocephalus glaber OX=10181 GN=GW7_21777 PE=4 SV=1
**G5BVG8**	Succinate--CoA ligase [ADP/GDP-forming] subunit alpha, mitochondrial OS=Heterocephalus glaber OX=10181 GN=SUCLG1 PE=3 SV=1
**G5B4Y6**	Beta-adducin OS=Heterocephalus glaber OX=10181 GN=ADD2 PE=4 SV=1
**G5BCT6**	Platelet-activating factor acetylhydrolase IB subunit alpha OS=Heterocephalus glaber OX=10181 GN=GW7_05075 PE=3 SV=1
**G5BPB2**	NADH dehydrogenase [ubiquinone] iron-sulfur protein 3, mitochondrial OS=Heterocephalus glaber OX=10181 GN=GW7_11265 PE=3 SV=1
**G5AV13**	Sodium/calcium exchanger 2 OS=Heterocephalus glaber OX=10181 GN=GW7_18138 PE=3 SV=1
**G5BNX8**	ATP synthase subunit b, mitochondrial OS=Heterocephalus glaber OX=10181 GN=GW7_03064 PE=4 SV=1
**G5B651**	ARF GTPase-activating protein GIT2 OS=Heterocephalus glaber OX=10181 GN=GW7_18931 PE=4 SV=1
**G5AWL7**	Serine/threonine-protein phosphatase (Fragment) OS=Heterocephalus glaber OX=10181 GN=GW7_03093 PE=3 SV=1
**G5AYJ7**	4F2 cell-surface antigen heavy chain OS=Heterocephalus glaber OX=10181 GN=GW7_01791 PE=4 SV=1
**G5C7Y3**	V-type proton ATPase subunit D OS=Heterocephalus glaber OX=10181 GN=ATP6V1D PE=4 SV=1
**G5BN12**	Serine/threonine-protein phosphatase OS=Heterocephalus glaber OX=10181 GN=PPP2CA PE=3 SV=1
**G5C7G8**	Serine/threonine-protein phosphatase OS=Heterocephalus glaber OX=10181 GN=GW7_13156 PE=3 SV=1
**G5AL24**	Microtubule-associated protein OS=Heterocephalus glaber OX=10181 GN=GW7_13401 PE=4 SV=1
**G5BY58**	NADH dehydrogenase (Ubiquinone) 1 beta subcomplex subunit 10 OS=Heterocephalus glaber OX=10181 GN=NDUFB10 PE=4 SV=1
**G5BLA6**	Guanine nucleotide-binding protein G(Q) subunit alpha OS=Heterocephalus glaber OX=10181 GN=GW7_20311 PE=4 SV=1
**G5BL47**	Cytochrome b-c1 complex subunit Rieske, mitochondrial OS=Heterocephalus glaber OX=10181 GN=UQCRFS1 PE=4 SV=1
**G5BN01**	Protein RUFY3 OS=Heterocephalus glaber OX=10181 GN=GW7_20521 PE=4 SV=1
**G5BEP8**	Tyrosine-protein phosphatase non-receptor type substrate 1 OS=Heterocephalus glaber OX=10181 GN=GW7_13443 PE=4 SV=1
**G5BRC3**	Coronin OS=Heterocephalus glaber OX=10181 GN=GW7_14880 PE=3 SV=1
**G5AS68**	Phosphodiesterase OS=Heterocephalus glaber OX=10181 GN=GW7_02116 PE=3 SV=1

**G5APR2**	Cytosol aminopeptidase OS=Heterocephalus glaber OX=10181 GN=LAP3 PE=3 SV=1
**G5BIK6**	Calcium/calmodulin-dependent protein kinase type II beta chain OS=Heterocephalus glaber OX=10181 GN=GW7_08267 PE=4 SV=1
**G5ASK0**	Proteasome subunit beta OS=Heterocephalus glaber OX=10181 GN=PSMB5 PE=3 SV=1
**G5C5U7**	Plectin-1 OS=Heterocephalus glaber OX=10181 GN=GW7_19255 PE=4 SV=1
**G5BDS2**	Cerebellin-3 OS=Heterocephalus glaber OX=10181 GN=GW7_16489 PE=4 SV=1
**G5CBD1**	Beta-soluble NSF attachment protein OS=Heterocephalus glaber OX=10181 GN=GW7_00718 PE=4 SV=1
**G5C2S9**	Heterogeneous nuclear ribonucleoprotein H2 OS=Heterocephalus glaber OX=10181 GN=GW7_14140 PE=4 SV=1
**G5ASA2**	Ras-related protein Rab-6A OS=Heterocephalus glaber OX=10181 GN=GW7_02105 PE=4 SV=1
**G5B2N8**	Vacuolar protein sorting-associated protein 26B OS=Heterocephalus glaber OX=10181 GN=GW7_11540 PE=4 SV=1
**G5B8B7**	Clathrin coat assembly protein AP180 OS=Heterocephalus glaber OX=10181 GN=GW7_19308 PE=4 SV=1
**G5CAH6**	Alpha-centractin OS=Heterocephalus glaber OX=10181 GN=ACTR1A PE=3 SV=1
**G5B5W3**	Alanyl-tRNA synthetase, cytoplasmic OS=Heterocephalus glaber OX=10181 GN=AARS PE=3 SV=1
**G5BXJ3**	Myelin-associated glycoprotein isoform b OS=Heterocephalus glaber OX=10181 GN=MAG PE=4 SV=1
**G5AV01**	AP complex subunit sigma OS=Heterocephalus glaber OX=10181 GN=GW7_18126 PE=3 SV=1
**G5BUW4**	60S ribosomal protein L27a OS=Heterocephalus glaber OX=10181 GN=RPL27A PE=3 SV=1
**G5BA18**	CB1 cannabinoid receptor-interacting protein 1 OS=Heterocephalus glaber OX=10181 GN=GW7_02210 PE=4 SV=1
**G5CA61**	Four and a half LIM domains protein 1 OS=Heterocephalus glaber OX=10181 GN=GW7_15573 PE=4 SV=1
**G5BJG1**	Cytochrome c oxidase subunit 4 isoform 1, mitochondrial OS=Heterocephalus glaber OX=10181 GN=GW7_12651 PE=4 SV=1
**G5B9Z4**	Importin subunit alpha OS=Heterocephalus glaber OX=10181 GN=GW7_05375 PE=3 SV=1
**G5B782**	60S acidic ribosomal protein P0 OS=Heterocephalus glaber OX=10181 GN=RPLP0 PE=3 SV=1
**G5BBE6**	CUG-BP-and ETR-3-like factor 2 (Fragment) OS=Heterocephalus glaber OX=10181 GN=GW7_19731 PE=4 SV=1
**G5BTC4**	26S proteasome non-ATPase regulatory subunit 2 OS=Heterocephalus glaber OX=10181 GN=GW7_17231 PE=3 SV=1
**G5AZR4**	Platelet-activating factor acetylhydrolase IB subunit gamma OS=Heterocephalus glaber OX=10181 GN=PAFAH1B3 PE=4 SV=1
**G5ALN8**	Heterogeneous nuclear ribonucleoprotein D0 (Fragment) OS=Heterocephalus glaber OX=10181 GN=GW7_10606 PE=4 SV=1
**G5B230**	Intercellular adhesion molecule 5 OS=Heterocephalus glaber OX=10181 GN=GW7_08246 PE=4 SV=1
**G5C3G6**	Heat shock 70 kDa protein 1B OS=Heterocephalus glaber OX=10181 GN=GW7_04796 PE=3 SV=1
**G5B623**	Guanine nucleotide-binding protein G(Z) subunit alpha OS=Heterocephalus glaber OX=10181 GN=GW7_18903 PE=4 SV=1
**G5ATL5**	Phosphoinositide phospholipase C OS=Heterocephalus glaber OX=10181 GN=GW7_07363 PE=4 SV=1
**G5BZ60**	Adenylyl cyclase-associated protein OS=Heterocephalus glaber OX=10181 GN=GW7_06073 PE=3 SV=1
**G5C720**	Glycogen debranching enzyme OS=Heterocephalus glaber OX=10181 GN=GW7_08051 PE=4 SV=1
**G5BQA1**	GTPase KRas isoform b OS=Heterocephalus glaber OX=10181 GN=KRAS PE=4 SV=1
**G5BUE2**	Proteasome subunit alpha type (Fragment) OS=Heterocephalus glaber OX=10181 GN=GW7_21362 PE=3 SV=1
**G5C6E9**	Myotubularin OS=Heterocephalus glaber OX=10181 GN=GW7_05400 PE=4 SV=1
**G5BDX2**	Proteasome subunit alpha type OS=Heterocephalus glaber OX=10181 GN=PSMA7 PE=3 SV=1
**G5C1E9**	Leucine-rich repeat and calponin-like protein domain-containing protein 1 OS=Heterocephalus glaber OX=10181 GN=GW7_16595 PE=4 SV=1
**G5ALG5**	U2-associated protein SR140 OS=Heterocephalus glaber OX=10181 GN=GW7_01159 PE=4 SV=1
**G5B327**	Plexin-A1 OS=Heterocephalus glaber OX=10181 GN=GW7_03027 PE=4 SV=1
**G5AYG1**	Cleavage and polyadenylation specificity factor subunit 7 OS=Heterocephalus glaber OX=10181 GN=GW7_01755 PE=4 SV=1

**G5B9Q1**	Phytanoyl-CoA hydroxylase-interacting protein OS=Heterocephalus glaber OX=10181 GN=GW7_20727 PE=4 SV=1
**G5BZN6**	Succinate dehydrogenase [ubiquinone] iron-sulfur subunit, mitochondrial OS=Heterocephalus glaber OX=10181 GN=GW7_09699 PE=3 SV=1
**G5C4R0**	Succinyl-CoA:3-ketoacid-coenzyme A transferase OS=Heterocephalus glaber OX=10181 GN=GW7_19761 PE=3 SV=1
**G5BX86**	Tubulin monoglycylase TTLL3 OS=Heterocephalus glaber OX=10181 GN=GW7_19486 PE=4 SV=1
**G5C662**	V-type proton ATPase subunit OS=Heterocephalus glaber OX=10181 GN=GW7_02358 PE=3 SV=1
**G5BBH0**	Ras-related protein Rap-1b isoform 1 OS=Heterocephalus glaber OX=10181 GN=RAP1B PE=4 SV=1
**G5BJU2**	Septin-4 (Fragment) OS=Heterocephalus glaber OX=10181 GN=GW7_05221 PE=3 SV=1
**G5CAD2**	Phosphatidylinositol-binding clathrin assembly protein OS=Heterocephalus glaber OX=10181 GN=GW7_14144 PE=4 SV=1
**G5BCG0**	Protein kinase C and casein kinase substrate in neurons protein 1 OS=Heterocephalus glaber OX=10181 GN=GW7_15650 PE=4 SV=1
**G5C053**	Clathrin heavy chain OS=Heterocephalus glaber OX=10181 GN=GW7_01835 PE=3 SV=1
**G5CAR4**	Ubiquitin carboxyl-terminal hydrolase OS=Heterocephalus glaber OX=10181 GN=USP5 PE=3 SV=1
**G5CBG2**	40S ribosomal protein S9 OS=Heterocephalus glaber OX=10181 GN=RPS9 PE=3 SV=1
**G5BM53**	Protein disulfide-isomerase OS=Heterocephalus glaber OX=10181 GN=GW7_18775 PE=3 SV=1
**G5B5J9**	Tyrosine--tRNA ligase OS=Heterocephalus glaber OX=10181 GN=GW7_11504 PE=3 SV=1
**G5B6P2**	Neurexin-2-alpha OS=Heterocephalus glaber OX=10181 GN=GW7_20664 PE=4 SV=1
**G5C7B5**	Calnexin OS=Heterocephalus glaber OX=10181 GN=GW7_16684 PE=3 SV=1
**G5BV64**	Protein kinase C beta type (Fragment) OS=Heterocephalus glaber OX=10181 GN=GW7_04700 PE=3 SV=1
**G5B2L9**	Rogdi-like protein OS=Heterocephalus glaber OX=10181 GN=GW7_19978 PE=4 SV=1
**G5AT31**	Versican core protein (Fragment) OS=Heterocephalus glaber OX=10181 GN=GW7_17141 PE=4 SV=1
**G5AN52**	Ras-related protein Rab-11A OS=Heterocephalus glaber OX=10181 GN=RAB11A PE=4 SV=1
**G5AVA6**	Enoyl-CoA hydratase, mitochondrial OS=Heterocephalus glaber OX=10181 GN=GW7_07876 PE=3 SV=1
**G5AZ44**	Putative cysteinyl-tRNA synthetase, mitochondrial OS=Heterocephalus glaber OX=10181 GN=GW7_00286 PE=3 SV=1
**G5BUU3**	Mitochondrial import receptor subunit TOM70 OS=Heterocephalus glaber OX=10181 GN=GW7_21055 PE=4 SV=1
**G5BLB9**	Phytanoyl-CoA hydroxylase-interacting protein-like isoform 1 OS=Heterocephalus glaber OX=10181 GN=PHYHIPL PE=4 SV=1
**G5CBK6**	Trifunctional enzyme subunit beta, mitochondrial OS=Heterocephalus glaber OX=10181 GN=GW7_17038 PE=3 SV=1
**G5AKI4**	Anion exchange protein OS=Heterocephalus glaber OX=10181 GN=GW7_07264 PE=3 SV=1
**G5BT43**	Voltage-dependent anion-selective channel protein 3 isoform 2 OS=Heterocephalus glaber OX=10181 GN=VDAC3 PE=4 SV=1
**G5AU15**	Pre-mRNA-processing factor 40-like protein B OS=Heterocephalus glaber OX=10181 GN=GW7_14447 PE=4 SV=1
**G5BEG5**	N-acylneuraminate cytidylyltransferase OS=Heterocephalus glaber OX=10181 GN=GW7_05662 PE=4 SV=1
**G5AQT3**	Septin-9 (Fragment) OS=Heterocephalus glaber OX=10181 GN=GW7_06407 PE=3 SV=1
**G5C1G0**	Guanine nucleotide-binding protein subunit beta-4 (Fragment) OS=Heterocephalus glaber OX=10181 GN=GW7_14113 PE=4 SV=1
**G5BF08**	Homer protein-like protein 2 OS=Heterocephalus glaber OX=10181 GN=GW7_13593 PE=4 SV=1
**G5AR83**	Brevican core protein OS=Heterocephalus glaber OX=10181 GN=GW7_14272 PE=4 SV=1
**G5BF97**	DNA topoisomerase 2-beta OS=Heterocephalus glaber OX=10181 GN=GW7_15047 PE=4 SV=1
**G5B9C3**	Stromal interaction molecule 1 (Fragment) OS=Heterocephalus glaber OX=10181 GN=GW7_12631 PE=4 SV=1
**G5B5Y3**	40S ribosomal protein S4 OS=Heterocephalus glaber OX=10181 GN=GW7_15480 PE=3 SV=1
**G5CAJ1**	Up-regulated during skeletal muscle growth protein 5 OS=Heterocephalus glaber OX=10181 GN=GW7_15920 PE=4 SV=1
**G5AV28**	Synaptic vesicle glycoprotein 2B OS=Heterocephalus glaber OX=10181 GN=GW7_05778 PE=4 SV=1

**G5BAI1**	IQ motif and SEC7 domain-containing protein 1 OS=Heterocephalus glaber OX=10181 GN=GW7_02159 PE=4 SV=1
**G5BLN1**	Atlastin-1 (Fragment) OS=Heterocephalus glaber OX=10181 GN=GW7_00228 PE=4 SV=1
**G5AXV0**	Catalase OS=Heterocephalus glaber OX=10181 GN=GW7_08865 PE=3 SV=1
**G5BBP0**	2,4-dienoyl-CoA reductase, mitochondrial OS=Heterocephalus glaber OX=10181 GN=GW7_09360 PE=4 SV=1
**G5B890**	60S ribosomal protein L18 OS=Heterocephalus glaber OX=10181 GN=GW7_05359 PE=4 SV=1
**G5AXI1**	Dual specificity tyrosine-phosphorylation-regulated kinase 1A (Fragment) OS=Heterocephalus glaber OX=10181 GN=GW7_17149 PE=4 SV=1
**G5BK72**	Creatine kinase S-type, mitochondrial OS=Heterocephalus glaber OX=10181 GN=GW7_10879 PE=3 SV=1
**G5AUK3**	Proteasome subunit alpha type (Fragment) OS=Heterocephalus glaber OX=10181 GN=GW7_10861 PE=3 SV=1
**G5C077**	AFG3-like protein 2 (Fragment) OS=Heterocephalus glaber OX=10181 GN=GW7_12057 PE=3 SV=1
**G5BQW4**	Proteasome subunit alpha type OS=Heterocephalus glaber OX=10181 GN=PSMA5 PE=3 SV=1
**G5BRL1**	Switch-associated protein 70 OS=Heterocephalus glaber OX=10181 GN=SWAP70 PE=4 SV=1
**G5BJ78**	Rho GDP-dissociation inhibitor 1 OS=Heterocephalus glaber OX=10181 GN=ARHGDIA PE=4 SV=1
**G5B9K1**	Putative ubiquitin carboxyl-terminal hydrolase FAF-X OS=Heterocephalus glaber OX=10181 GN=GW7_04024 PE=3 SV=1
**G5B371**	Liprin-alpha-4 OS=Heterocephalus glaber OX=10181 GN=GW7_12850 PE=4 SV=1
**G5BII7**	Dolichyl-diphosphooligosaccharide--protein glycosyltransferase subunit 1 OS=Heterocephalus glaber OX=10181 GN=GW7_10957 PE=3 SV=1
**G5C5Z1**	Amino acid transporter OS=Heterocephalus glaber OX=10181 GN=GW7_18352 PE=3 SV=1
**G5BFV5**	Heterogeneous nuclear ribonucleoproteins C1/C2 OS=Heterocephalus glaber OX=10181 GN=GW7_08510 PE=4 SV=1
**G5C9Y1**	Phospholipase D3 OS=Heterocephalus glaber OX=10181 GN=GW7_03571 PE=4 SV=1
**G5BH20**	Histone H2B OS=Heterocephalus glaber OX=10181 GN=GW7_05783 PE=3 SV=1
**G5BZ00**	Zinc transporter 3 OS=Heterocephalus glaber OX=10181 GN=GW7_14384 PE=4 SV=1
**G5BH46**	Transmembrane protein 65 (Fragment) OS=Heterocephalus glaber OX=10181 GN=GW7_05809 PE=4 SV=1
**G5AX60**	Peroxisomal multifunctional enzyme type 2 OS=Heterocephalus glaber OX=10181 GN=GW7_07527 PE=4 SV=1
**G5BIL3**	Peptidyl-prolyl cis-trans isomerase OS=Heterocephalus glaber OX=10181 GN=PPIA PE=3 SV=1
**G5AN45**	NADH dehydrogenase [ubiquinone] 1 alpha subcomplex subunit 13 OS=Heterocephalus glaber OX=10181 GN=GW7_05858 PE=4 SV=1
**G5BAR1**	Cytosolic non-specific dipeptidase OS=Heterocephalus glaber OX=10181 GN=GW7_18554 PE=4 SV=1
**G5BID1**	Phosphodiesterase OS=Heterocephalus glaber OX=10181 GN=PDE1B PE=3 SV=1
**G5AZ20**	Gamma-aminobutyric acid type B receptor subunit 2 OS=Heterocephalus glaber OX=10181 GN=GW7_05037 PE=3 SV=1
**G5AQ32**	4-trimethylaminobutyraldehyde dehydrogenase OS=Heterocephalus glaber OX=10181 GN=ALDH9A1 PE=3 SV=1
**G5ATC2**	Rho GTPase-activating protein 1 OS=Heterocephalus glaber OX=10181 GN=GW7_01178 PE=4 SV=1
**G5C1J8**	Neurexin-1-alpha OS=Heterocephalus glaber OX=10181 GN=GW7_16388 PE=4 SV=1
**G5AQN1**	COP9 signalosome complex subunit 5 OS=Heterocephalus glaber OX=10181 GN=COPS5 PE=4 SV=1
**G5BWQ7**	High affinity copper uptake protein 1 OS=Heterocephalus glaber OX=10181 GN=GW7_09545 PE=4 SV=1
**G5BYY8**	Microtubule-associated protein RP/EB family member 3 OS=Heterocephalus glaber OX=10181 GN=MAPRE3 PE=4 SV=1
**G5BL96**	Keratin, type II cytoskeletal 7 OS=Heterocephalus glaber OX=10181 GN=GW7_04045 PE=3 SV=1
**G5BZL6**	Aflatoxin B1 aldehyde reductase member 2 OS=Heterocephalus glaber OX=10181 GN=GW7_09679 PE=4 SV=1
**G5C9N0**	DNA topoisomerase I (Fragment) OS=Heterocephalus glaber OX=10181 GN=GW7_18071 PE=3 SV=1
**G5B5Y6**	Lysine--tRNA ligase OS=Heterocephalus glaber OX=10181 GN=KARS PE=3 SV=1

**G5BHZ1**	Heterogeneous nuclear ribonucleoprotein R OS=Heterocephalus glaber OX=10181 GN=GW7_07501 PE=4 SV=1
**G5BPT7**	Adenosylhomocysteinase OS=Heterocephalus glaber OX=10181 GN=AHCY PE=3 SV=1
**G5AKZ3**	Guanine nucleotide-binding protein G(S) subunit alpha isoforms XLas OS=Heterocephalus glaber OX=10181 GN=GW7_08852 PE=4 SV=1
**G5B8C9**	OCIA domain-containing protein 2 (Fragment) OS=Heterocephalus glaber OX=10181 GN=GW7_00862 PE=4 SV=1
**G5ASC5**	Carnitine O-acetyltransferase isoform 1 OS=Heterocephalus glaber OX=10181 GN=CRAT PE=3 SV=1
**G5BKZ8**	Talin-1 OS=Heterocephalus glaber OX=10181 GN=GW7_10700 PE=4 SV=1
**G5CAI7**	Cytosolic purine 5'-nucleotidase OS=Heterocephalus glaber OX=10181 GN=GW7_15916 PE=4 SV=1
**G5C5B6**	Homer protein-like protein 3 OS=Heterocephalus glaber OX=10181 GN=GW7_11673 PE=4 SV=1
**G5C5Z4**	Actin-related protein 2 OS=Heterocephalus glaber OX=10181 GN=GW7_18355 PE=3 SV=1
**G5BLW4**	Retinal dehydrogenase 1 (Fragment) OS=Heterocephalus glaber OX=10181 GN=GW7_02633 PE=3 SV=1
**G5AQH6**	Solute carrier family 2, facilitated glucose transporter member 1 (Fragment) OS=Heterocephalus glaber OX=10181 GN=GW7_04364 PE=3 SV=1
**G5BPY0**	Sidoreflexin OS=Heterocephalus glaber OX=10181 GN=SFXN1 PE=3 SV=1
**G5AY95**	NADH dehydrogenase (Ubiquinone) 1 beta subcomplex subunit 9 OS=Heterocephalus glaber OX=10181 GN=NDUFB9 PE=3 SV=1
**G5B6G5**	Reticulon OS=Heterocephalus glaber OX=10181 GN=GW7_20694 PE=4 SV=1
**G5CBM7**	Apolipoprotein E OS=Heterocephalus glaber OX=10181 GN=GW7_18161 PE=2 SV=1
**G5C705**	Leucine-rich glioma-inactivated protein 1 OS=Heterocephalus glaber OX=10181 GN=LGI1 PE=4 SV=1
**G5BYW3**	Glutaryl-CoA dehydrogenase, mitochondrial OS=Heterocephalus glaber OX=10181 GN=GW7_16083 PE=3 SV=1
**2G5BKI4**	Cell division control protein 42-like protein OS=Heterocephalus glaber OX=10181 GN=GW7_09970 PE=4 SV=1
**G5BLW6**	Retinal dehydrogenase 1 OS=Heterocephalus glaber OX=10181 GN=GW7_02635 PE=3 SV=1
**G5B6W3**	60S ribosomal protein L35 OS=Heterocephalus glaber OX=10181 GN=RPL35 PE=3 SV=1
**G5ASS1**	NipSnap-like protein 2 (Fragment) OS=Heterocephalus glaber OX=10181 GN=GW7_14812 PE=4 SV=1
**G5BIN6**	Synaptic vesicle membrane protein VAT-1-like protein (Fragment) OS=Heterocephalus glaber OX=10181 GN=GW7_16839 PE=4 SV=1
**G5BSN9**	Putative tyrosine-protein phosphatase auxilin OS=Heterocephalus glaber OX=10181 GN=GW7_01514 PE=4 SV=1
**G5BJS5**	Aquaporin-4 OS=Heterocephalus glaber OX=10181 GN=GW7_07902 PE=3 SV=1
**G5BQT6**	B-cell receptor-associated protein 31 OS=Heterocephalus glaber OX=10181 GN=GW7_02021 PE=4 SV=1
**G5BCJ5**	Uncharacterized protein OS=Heterocephalus glaber OX=10181 GN=GW7_18963 PE=4 SV=1
**G5ATW7**	Carbonic anhydrase 2 (Fragment) OS=Heterocephalus glaber OX=10181 GN=GW7_14399 PE=4 SV=1
**G5BAZ8**	Zinc transporter SLC39A7 OS=Heterocephalus glaber OX=10181 GN=SLC39A7 PE=4 SV=1
**G5BKB7**	Gamma-adducin OS=Heterocephalus glaber OX=10181 GN=GW7_14624 PE=4 SV=1
**G5AQS0**	60 kDa heat shock protein, mitochondrial OS=Heterocephalus glaber OX=10181 GN=GW7_10597 PE=4 SV=1
**G5ATA4**	Collagen alpha-1(XVIII) chain OS=Heterocephalus glaber OX=10181 GN=GW7_13728 PE=4 SV=1
**G5B5C8**	Eukaryotic initiation factor 4A-I OS=Heterocephalus glaber OX=10181 GN=GW7_13830 PE=3 SV=1
**G5AR57**	NADH dehydrogenase [ubiquinone] 1 alpha subcomplex subunit 2 (Fragment) OS=Heterocephalus glaber OX=10181 GN=GW7_12539 PE=4 SV=1
**G5BLQ4**	Oxysterol-binding protein OS=Heterocephalus glaber OX=10181 GN=GW7_09395 PE=3 SV=1
**G5C0T9**	NADH dehydrogenase [ubiquinone] 1 alpha subcomplex subunit 12 OS=Heterocephalus glaber OX=10181 GN=NDUFA12 PE=3 SV=1
**G5BT86**	Kininogen-1 OS=Heterocephalus glaber OX=10181 GN=KNG1 PE=4 SV=1
**G5AQL0**	Hydroxyacyl-coenzyme A dehydrogenase, mitochondrial OS=Heterocephalus glaber OX=10181 GN=GW7_17857 PE=4 SV=1

**G5BY67**	28S ribosomal protein S34, mitochondrial OS=Heterocephalus glaber OX=10181 GN=GW7_05752 PE=4 SV=1
**G5BYT3**	cAMP-dependent protein kinase catalytic subunit alpha OS=Heterocephalus glaber OX=10181 GN=GW7_16053 PE=3 SV=1
**G5C2R7**	Methyl-CpG-binding protein 2 OS=Heterocephalus glaber OX=10181 GN=GW7_13707 PE=4 SV=1
**G5BML0**	Pleiotropic regulator 1 OS=Heterocephalus glaber OX=10181 GN=GW7_21603 PE=4 SV=1
**G5BY35**	p130Cas-associated protein OS=Heterocephalus glaber OX=10181 GN=GW7_00459 PE=4 SV=1
**G5B2A2**	Copine-6 OS=Heterocephalus glaber OX=10181 GN=GW7_11974 PE=4 SV=1
**G5AN82**	Dual specificity mitogen-activated protein kinase kinase 1 OS=Heterocephalus glaber OX=10181 GN=GW7_19660 PE=3 SV=1
**G5BJV7**	Adipocyte plasma membrane-associated protein OS=Heterocephalus glaber OX=10181 GN=APMAP PE=4 SV=1
**G5BSC6**	Ubiquitin carboxyl-terminal hydrolase 7 OS=Heterocephalus glaber OX=10181 GN=GW7_08972 PE=3 SV=1
**G5BLB3**	CDGSH iron sulfur domain-containing protein 1 OS=Heterocephalus glaber OX=10181 GN=GW7_01960 PE=4 SV=1
**G5BYS2**	NADH dehydrogenase [ubiquinone] 1 beta subcomplex subunit 7 OS=Heterocephalus glaber OX=10181 GN=GW7_16042 PE=4 SV=1
**G5CBQ7**	Sideroflexin-5 OS=Heterocephalus glaber OX=10181 GN=GW7_03299 PE=4 SV=1
**G5AKY1**	Neuronal membrane glycoprotein M6-b OS=Heterocephalus glaber OX=10181 GN=GW7_16652 PE=4 SV=1
**G5BDJ8**	WD repeat-containing protein 68 OS=Heterocephalus glaber OX=10181 GN=GW7_17461 PE=4 SV=1
**G5BI03**	Cell division control protein 42-like protein OS=Heterocephalus glaber OX=10181 GN=GW7_07513 PE=4 SV=1
**G5C350**	Importin-5 OS=Heterocephalus glaber OX=10181 GN=GW7_12714 PE=4 SV=1
**G5BRW3**	Ribosomal protein L19 OS=Heterocephalus glaber OX=10181 GN=GW7_09589 PE=3 SV=1
**G5C9W7**	Ras-related protein Rab-4B (Fragment) OS=Heterocephalus glaber OX=10181 GN=GW7_03557 PE=4 SV=1
**G5ASV2**	Nucleolin OS=Heterocephalus glaber OX=10181 GN=GW7_10373 PE=4 SV=1
**G5AKW2**	RasGAP-activating-like protein 1 OS=Heterocephalus glaber OX=10181 GN=GW7_10151 PE=4 SV=1
**G5B6U4**	Ras-related protein Ral-A OS=Heterocephalus glaber OX=10181 GN=RALA PE=4 SV=1
**G5C0D0**	Neuronal cell adhesion molecule OS=Heterocephalus glaber OX=10181 GN=GW7_06596 PE=4 SV=1
**G5BMR5**	Guanine nucleotide-binding protein G(O) subunit alpha OS=Heterocephalus glaber OX=10181 GN=GW7_09345 PE=4 SV=1
**G5B6W7**	Proteasome subunit beta OS=Heterocephalus glaber OX=10181 GN=GW7_01146 PE=3 SV=1
**G5AU14**	Formin-like protein 3 OS=Heterocephalus glaber OX=10181 GN=GW7_14446 PE=4 SV=1
**G5BZP0**	Phosphoacetylglucosamine mutase OS=Heterocephalus glaber OX=10181 GN=GW7_08293 PE=3 SV=1
**G5BK63**	Proteasome subunit beta OS=Heterocephalus glaber OX=10181 GN=GW7_13065 PE=3 SV=1
**G5B389**	Endothelin B receptor-like protein 2 OS=Heterocephalus glaber OX=10181 GN=GW7_12868 PE=4 SV=1
**G5C2E8**	Tetraspanin OS=Heterocephalus glaber OX=10181 GN=GW7_00385 PE=3 SV=1
**G5BMX3**	Myosin-XV OS=Heterocephalus glaber OX=10181 GN=GW7_07656 PE=3 SV=1
**G5C351**	FERM, RhoGEF and pleckstrin domain-containing protein 1 (Fragment) OS=Heterocephalus glaber OX=10181 GN=GW7_12715 PE=4 SV=1
**G5BS94**	Glutathione S-transferase P OS=Heterocephalus glaber OX=10181 GN=GW7_14677 PE=3 SV=1
**G5B117**	Proteasome subunit alpha type OS=Heterocephalus glaber OX=10181 GN=GW7_14185 PE=3 SV=1
**G5C384**	Pre-mRNA branch site protein p14 OS=Heterocephalus glaber OX=10181 GN=GW7_13983 PE=4 SV=1
**G5BY52**	Synaptogyrin-3 (Fragment) OS=Heterocephalus glaber OX=10181 GN=GW7_05737 PE=4 SV=1
**G5AK81**	Anion exchange protein (Fragment) OS=Heterocephalus glaber OX=10181 GN=GW7_08981 PE=3 SV=1
**G5AWP7**	GTP-binding nuclear protein Ran (Fragment) OS=Heterocephalus glaber OX=10181 GN=GW7_03170 PE=3 SV=1
**G5BSE8**	Histone H2A OS=Heterocephalus glaber OX=10181 GN=GW7_09999 PE=3 SV=1

**G5C226**	Sodium-and chloride-dependent GABA transporter 3 OS=Heterocephalus glaber OX=10181 GN=GW7_20151 PE=4 SV=1
**G5B211**	28S ribosomal protein S27, mitochondrial (Fragment) OS=Heterocephalus glaber OX=10181 GN=GW7_19014 PE=4 SV=1
**G5BNW3**	Rho-related GTP-binding protein RhoC OS=Heterocephalus glaber OX=10181 GN=GW7_03049 PE=4 SV=1
**G5BG13**	6-phosphofructokinase, liver type OS=Heterocephalus glaber OX=10181 GN=GW7_06815 PE=4 SV=1
**G5B6E8**	Isocitrate dehydrogenase [NADP] OS=Heterocephalus glaber OX=10181 GN=IDH1 PE=3 SV=1
**G5BQT9**	Isocitrate dehydrogenase [NAD] subunit, mitochondrial OS=Heterocephalus glaber OX=10181 GN=GW7_02024 PE=3 SV=1
**G5BVV5**	Eukaryotic translation initiation factor 3 subunit A OS=Heterocephalus glaber OX=10181 GN=EIF3A PE=3 SV=1
**G5AVX4**	Phosphatidylinositol-5-phosphate 4-kinase type-2 alpha OS=Heterocephalus glaber OX=10181 GN=GW7_05903 PE=4 SV=1
**G5BCB4**	Annexin OS=Heterocephalus glaber OX=10181 GN=GW7_14853 PE=3 SV=1
**G5BRM9**	Glycyl-tRNA synthetase OS=Heterocephalus glaber OX=10181 GN=GW7_21726 PE=4 SV=1
**G5BX33**	Heat shock protein HSP 90-alpha 1 OS=Heterocephalus glaber OX=10181 GN=GW7_11862 PE=4 SV=1
**G5B8W2**	Reticulon (Fragment) OS=Heterocephalus glaber OX=10181 GN=GW7_15257 PE=4 SV=1
**G5BCL0**	ATP synthase subunit e, mitochondrial OS=Heterocephalus glaber OX=10181 GN=GW7_18978 PE=4 SV=1
**G5C8J0**	Netrin-G1 ligand OS=Heterocephalus glaber OX=10181 GN=GW7_15729 PE=4 SV=1
**G5BD34**	C-4 methylsterol oxidase OS=Heterocephalus glaber OX=10181 GN=GW7_07430 PE=3 SV=1
**G5C0Q7**	MICOS complex subunit MIC13 OS=Heterocephalus glaber OX=10181 GN=GW7_20494 PE=3 SV=1
**G5BMJ9**	Tripartite motif-containing protein 2 OS=Heterocephalus glaber OX=10181 GN=GW7_21592 PE=4 SV=1
**G5C8B4**	DnaJ homolog subfamily C member 5 OS=Heterocephalus glaber OX=10181 GN=DNAJC5 PE=4 SV=1
**G5BMM2**	Pleckstrin-like protein domain-containing family A member 6 OS=Heterocephalus glaber OX=10181 GN=GW7_11215 PE=4 SV=1
**G5BG23**	Pyridoxal kinase OS=Heterocephalus glaber OX=10181 GN=GW7_06825 PE=4 SV=1
**G5B9Q5**	Zinc transporter ZIP14 (Fragment) OS=Heterocephalus glaber OX=10181 GN=GW7_20731 PE=4 SV=1
**G5BNV9**	Monocarboxylate transporter 1 OS=Heterocephalus glaber OX=10181 GN=GW7_03045 PE=2 SV=1
**G5ATA6**	Poly(RC)-binding protein 3 OS=Heterocephalus glaber OX=10181 GN=GW7_13730 PE=4 SV=1
**G5B6T4**	Presequence protease, mitochondrial OS=Heterocephalus glaber OX=10181 GN=GW7_21507 PE=4 SV=1
**G5ANV7**	Phosphoglycerate mutase family member 5 OS=Heterocephalus glaber OX=10181 GN=GW7_09572 PE=4 SV=1
**G5C9Y4**	E3 ubiquitin-protein ligase HUWE1 OS=Heterocephalus glaber OX=10181 GN=GW7_13236 PE=4 SV=1
**G5BMH1**	Filamin-B OS=Heterocephalus glaber OX=10181 GN=GW7_03972 PE=4 SV=1
**G5AZK8**	NADH dehydrogenase (Ubiquinone) iron-sulfur protein 7, mitochondrial OS=Heterocephalus glaber OX=10181 GN=NDUFS7 PE=3 SV=1
**G5AVC6**	UDP-glucuronosyltransferase OS=Heterocephalus glaber OX=10181 GN=GW7_15973 PE=3 SV=1
**G5BPH4**	Tubulin polymerization-promoting protein OS=Heterocephalus glaber OX=10181 GN=GW7_14713 PE=4 SV=1
**G5BRJ4**	Induced myeloid leukemia cell differentiation protein Mcl-1-like protein OS=Heterocephalus glaber OX=10181 GN=GW7_20578 PE=4 SV=1
**G5BF40**	Ras-related C3 botulinum toxin substrate 1 (Fragment) OS=Heterocephalus glaber OX=10181 GN=GW7_17917 PE=4 SV=1
**G5BII9**	Putative ubiquitin carboxyl-terminal hydrolase FAF-Y OS=Heterocephalus glaber OX=10181 GN=GW7_10330 PE=3 SV=1
**G5BZV1**	Inosine-5'-monophosphate dehydrogenase OS=Heterocephalus glaber OX=10181 GN=IMPDH2 PE=3 SV=1
**G5AQ53**	Mitochondrial pyruvate carrier OS=Heterocephalus glaber OX=10181 GN=GW7_21171 PE=3 SV=1

**G5BMZ1**	Microtubule-associated protein OS=Heterocephalus glaber OX=10181 GN=GW7_07674 PE=4 SV=1
**G5BYS8**	ATP-dependent RNA helicase DDX39 OS=Heterocephalus glaber OX=10181 GN=GW7_16048 PE=4 SV=1
**G5B8W4**	40S ribosomal protein S27a OS=Heterocephalus glaber OX=10181 GN=RPS27A PE=4 SV=1
**G5AYV0**	Synaptogyrin-1 (Fragment) OS=Heterocephalus glaber OX=10181 GN=GW7_02597 PE=4 SV=1
**G5B151**	YTH domain-containing protein 1 OS=Heterocephalus glaber OX=10181 GN=GW7_09283 PE=4 SV=1
**G5AMJ9**	Microtubule-actin cross-linking factor 1, isoform 4 OS=Heterocephalus glaber OX=10181 GN=GW7_20337 PE=4 SV=1
**G5BFF6**	Cytochrome b-c1 complex subunit 8 OS=Heterocephalus glaber OX=10181 GN=UQCRQ PE=4 SV=1
**G5BQV7**	Seryl-tRNA synthetase, cytoplasmic OS=Heterocephalus glaber OX=10181 GN=GW7_20121 PE=4 SV=1
**G5APB6**	BTB/POZ domain-containing protein KCTD8 OS=Heterocephalus glaber OX=10181 GN=GW7_17018 PE=4 SV=1
**G5ARH5**	Gamma-aminobutyric acid receptor subunit beta-2 OS=Heterocephalus glaber OX=10181 GN=GW7_13529 PE=3 SV=1
**G5AQT1**	Bifunctional aminoacyl-tRNA synthetase OS=Heterocephalus glaber OX=10181 GN=GW7_09428 PE=3 SV=1
**G5C052**	Tricarboxylate transport protein, mitochondrial isoform a OS=Heterocephalus glaber OX=10181 GN=SLC25A1 PE=3 SV=1
**G5BT64**	Claudin OS=Heterocephalus glaber OX=10181 GN=GW7_13967 PE=3 SV=1
**G5C758**	Zinc transporter ZIP6 OS=Heterocephalus glaber OX=10181 GN=GW7_04828 PE=4 SV=1
**G5C6M4**	Casein kinase II subunit alpha OS=Heterocephalus glaber OX=10181 GN=GW7_20923 PE=4 SV=1
**G5API3**	60S ribosomal protein L13 OS=Heterocephalus glaber OX=10181 GN=RPL13 PE=3 SV=1
**G5C8G8**	Cell cycle exit and neuronal differentiation protein 1 OS=Heterocephalus glaber OX=10181 GN=CEND1 PE=4 SV=1
**G5AZJ5**	Actin-related protein 2/3 complex subunit 2 OS=Heterocephalus glaber OX=10181 GN=GW7_09502 PE=4 SV=1
**G5AJY0**	Histone-lysine N-methyltransferase OS=Heterocephalus glaber OX=10181 GN=GW7_10539 PE=3 SV=1
**G5AP00**	Polyadenylate-binding protein OS=Heterocephalus glaber OX=10181 GN=GW7_00765 PE=3 SV=1
**G5B441**	ELAV-like protein 1 OS=Heterocephalus glaber OX=10181 GN=GW7_09793 PE=4 SV=1
**G5BFW4**	Protein NDRG2 OS=Heterocephalus glaber OX=10181 GN=GW7_08519 PE=4 SV=1
**G5BJ57**	Brain-specific angiogenesis inhibitor 1-associated protein 2 OS=Heterocephalus glaber OX=10181 GN=GW7_04072 PE=4 SV=1
**G5ANZ0**	Cytochrome c oxidase subunit 6C OS=Heterocephalus glaber OX=10181 GN=GW7_00755 PE=4 SV=1
**G5ALG1**	SLIT-ROBO Rho GTPase-activating protein 1 OS=Heterocephalus glaber OX=10181 GN=GW7_05007 PE=4 SV=1
**G5AMV2**	Actin-related protein 3B (Fragment) OS=Heterocephalus glaber OX=10181 GN=GW7_02070 PE=3 SV=1
**G5BSL6**	Eukaryotic translation initiation factor 3 subunit E OS=Heterocephalus glaber OX=10181 GN=EIF3E PE=3 SV=1
**G5AYT2**	Putative ATP-dependent RNA helicase DDX17 OS=Heterocephalus glaber OX=10181 GN=GW7_02579 PE=3 SV=1
**G5BG17**	ES1 protein-like protein, mitochondrial OS=Heterocephalus glaber OX=10181 GN=GW7_06819 PE=4 SV=1
**G5BI50**	26S protease regulatory subunit 7 OS=Heterocephalus glaber OX=10181 GN=PSMC2 PE=3 SV=1
**G5BP41**	RNA-binding protein 25 OS=Heterocephalus glaber OX=10181 GN=GW7_04864 PE=4 SV=1
**G5BS22**	Aldo-keto reductase family 1 member B10 OS=Heterocephalus glaber OX=10181 GN=GW7_03813 PE=4 SV=1
**G5BV92**	Mu-crystallin-like protein OS=Heterocephalus glaber OX=10181 GN=CRYM PE=4 SV=1
**G5B8M4**	EH domain-containing protein 3 OS=Heterocephalus glaber OX=10181 GN=GW7_12819 PE=3 SV=1
**G5APW2**	Cytochrome b-c1 complex subunit 9 isoform b OS=Heterocephalus glaber OX=10181 GN=UQCR10 PE=4 SV=1
**G5C892**	Glycerophosphodiester phosphodiesterase domain-containing protein 1 isoform 1 OS=Heterocephalus glaber OX=10181 GN=GDPD1 PE=4 SV=1

**G5ATT8**	Proto-oncogene tyrosine-protein kinase Yes OS=Heterocephalus glaber OX=10181 GN=GW7_12511 PE=3 SV=1
**G5BG05**	Voltage-dependent N-type calcium channel subunit alpha (Fragment) OS=Heterocephalus glaber OX=10181 GN=GW7_03938 PE=3 SV=1
**G5AZE7**	Sodium/hydrogen exchanger (Fragment) OS=Heterocephalus glaber OX=10181 GN=GW7_06523 PE=3 SV=1
**G5C816**	Protein transport protein SEC23 OS=Heterocephalus glaber OX=10181 GN=GW7_07357 PE=3 SV=1
**G5B8T2**	Synaptotagmin-3 OS=Heterocephalus glaber OX=10181 GN=GW7_15329 PE=4 SV=1
**G5BCI6**	LETM1 and EF-hand domain-containing protein 1, mitochondrial OS=Heterocephalus glaber OX=10181 GN=GW7_18954 PE=4 SV=1
**G5AS20**	Phospholipid-transporting ATPase (Fragment) OS=Heterocephalus glaber OX=10181 GN=GW7_06616 PE=3 SV=1
**G5C2H5**	GTP-binding protein Di-Ras2 OS=Heterocephalus glaber OX=10181 GN=DIRAS2 PE=4 SV=1
**G5B4W2**	Phosphatidate cytidylyltransferase OS=Heterocephalus glaber OX=10181 GN=CDS2 PE=3 SV=1
**G5BFW6**	RSM22-like protein, mitochondrial OS=Heterocephalus glaber OX=10181 GN=GW7_08521 PE=4 SV=1
**G5AX92**	Pentatricopeptide repeat-containing protein 1 OS=Heterocephalus glaber OX=10181 GN=GW7_06631 PE=4 SV=1
**G5ALP8**	Putative saccharopine dehydrogenase OS=Heterocephalus glaber OX=10181 GN=SCCPDH PE=4 SV=1
**E3VX74**	40S ribosomal protein S27 OS=Heterocephalus glaber OX=10181 GN=GW7_11176 PE=2 SV=1
**G5BLV4**	Guanylate cyclase soluble subunit beta-1 (Fragment) OS=Heterocephalus glaber OX=10181 GN=GW7_04529 PE=3 SV=1
**G5B4M3**	NADH dehydrogenase (Ubiquinone) 1 alpha subcomplex subunit 7 OS=Heterocephalus glaber OX=10181 GN=NDUFA7 PE=4 SV=1
**G5BKP8**	Guanylate cyclase soluble subunit beta-2 OS=Heterocephalus glaber OX=10181 GN=GW7_20460 PE=3 SV=1
**G5ANX1**	Ribosomal protein L15 OS=Heterocephalus glaber OX=10181 GN=GW7_19814 PE=3 SV=1
**G5C396**	Platelet-derived growth factor receptor beta OS=Heterocephalus glaber OX=10181 GN=GW7_00120 PE=3 SV=1
**G5BD74**	Transporter OS=Heterocephalus glaber OX=10181 GN=GW7_07809 PE=3 SV=1
**G5B4R7**	Small nuclear ribonucleoprotein-associated protein B (Fragment) OS=Heterocephalus glaber OX=10181 GN=GW7_03184 PE=4 SV=1
**G5BY57**	40S ribosomal protein S2 OS=Heterocephalus glaber OX=10181 GN=GW7_05742 PE=3 SV=1
**G5BAV5**	Sulfurtransferase OS=Heterocephalus glaber OX=10181 GN=GW7_03677 PE=4 SV=1
**G5BQD5**	Histone H1.1 OS=Heterocephalus glaber OX=10181 GN=GW7_13921 PE=3 SV=1
**G5C4W8**	Dolichyl-diphosphooligosaccharide--protein glycosyltransferase 48 kDa subunit OS=Heterocephalus glaber OX=10181 GN=GW7_01845 PE=3 SV=1
**G5BY63**	Hydroxyacylglutathione hydrolase, mitochondrial OS=Heterocephalus glaber OX=10181 GN=GW7_05748 PE=3 SV=1
**G5AUX4**	Small nuclear ribonucleoprotein Sm D2 OS=Heterocephalus glaber OX=10181 GN=SNRPD2 PE=3 SV=1
**G5AZC2**	Polyadenylate-binding protein 1-like 2 OS=Heterocephalus glaber OX=10181 GN=GW7_11796 PE=4 SV=1
**G5BAR9**	Phosphoinositide phospholipase C (Fragment) OS=Heterocephalus glaber OX=10181 GN=GW7_19596 PE=4 SV=1
**G5B165**	Multidrug resistance-associated protein 4 OS=Heterocephalus glaber OX=10181 GN=ABCC4 PE=4 SV=1
**G5B8Q4**	Protein arginine N-methyltransferase 1 OS=Heterocephalus glaber OX=10181 GN=PRMT1 PE=3 SV=1
**G5AS88**	Phosphatidylinositol-5-phosphate 4-kinase type-2 gamma OS=Heterocephalus glaber OX=10181 GN=GW7_02091 PE=4 SV=1
**G5ARB5**	Histone-lysine N-methyltransferase OS=Heterocephalus glaber OX=10181 GN=GW7_14304 PE=4 SV=1
**G5BHN3**	Hypoxia up-regulated protein 1 OS=Heterocephalus glaber OX=10181 GN=GW7_18677 PE=3 SV=1
**G5BX32**	Heat shock protein HSP 90-alpha OS=Heterocephalus glaber OX=10181 GN=GW7_11861 PE=4 SV=1

**G5AZF7**	NADH dehydrogenase [ubiquinone] 1 beta subcomplex subunit 11, mitochondrial OS=Heterocephalus glaber OX=10181 GN=GW7_11280 PE=4 SV=1
**G5AVS2**	Rho-related GTP-binding protein RhoG OS=Heterocephalus glaber OX=10181 GN=RHOG PE=4 SV=1
**G5B3A8**	Kinesin-like protein KIF21B OS=Heterocephalus glaber OX=10181 GN=GW7_12887 PE=3 SV=1
**G5BJZ6**	Proteasome subunit alpha type OS=Heterocephalus glaber OX=10181 GN=PSMA2 PE=3 SV=1
**G5B4Q8**	Protein FAM49B OS=Heterocephalus glaber OX=10181 GN=FAM49B PE=4 SV=1
**G5BA12**	Ewing's tumor-associated antigen 1 OS=Heterocephalus glaber OX=10181 GN=GW7_02204 PE=4 SV=1
**G5BPF6**	Amine oxidase [flavin-containing] A (Fragment) OS=Heterocephalus glaber OX=10181 GN=GW7_15340 PE=4 SV=1
**G5C147**	Ras-related protein Rab-18 OS=Heterocephalus glaber OX=10181 GN=GW7_13698 PE=4 SV=1
**G5BWA4**	Proteasome subunit beta type-6 OS=Heterocephalus glaber OX=10181 GN=GW7_04618 PE=4 SV=1
**G5BTR3**	CDK5 and ABL1 enzyme substrate 1 (Fragment) OS=Heterocephalus glaber OX=10181 GN=GW7_00883 PE=4 SV=1
**G5BHY5**	60S ribosomal protein L11 (Fragment) OS=Heterocephalus glaber OX=10181 GN=GW7_07495 PE=3 SV=1
**G5BCE5**	Peroxiredoxin-4 OS=Heterocephalus glaber OX=10181 GN=PRDX4 PE=4 SV=1
**G5BN40**	LanC-like protein 1 OS=Heterocephalus glaber OX=10181 GN=GW7_18199 PE=4 SV=1
**G5AW66**	NADH dehydrogenase [ubiquinone] 1 subunit C2 OS=Heterocephalus glaber OX=10181 GN=GW7_00914 PE=3 SV=1
**G5B1Y9**	Whirlin OS=Heterocephalus glaber OX=10181 GN=GW7_01024 PE=4 SV=1
**G5BSB5**	G protein-coupled receptor kinase (Fragment) OS=Heterocephalus glaber OX=10181 GN=GW7_14698 PE=3 SV=1
**G5B1T3**	Aspartate--tRNA ligase, cytoplasmic OS=Heterocephalus glaber OX=10181 GN=DARS PE=3 SV=1
**G5AYQ6**	Zinc transporter 7 OS=Heterocephalus glaber OX=10181 GN=GW7_21185 PE=4 SV=1
**G5C2H3**	Methylglutaconyl-CoA hydratase, mitochondrial (Fragment) OS=Heterocephalus glaber OX=10181 GN=GW7_21658 PE=3 SV=1
**G5BTT2**	Nipped-B protein OS=Heterocephalus glaber OX=10181 GN=GW7_20141 PE=3 SV=1
**G5AU90**	NADH dehydrogenase (Ubiquinone) 1 beta subcomplex subunit 4 isoform 2 OS=Heterocephalus glaber OX=10181 GN=NDUFB4 PE=4 SV=1
**G5B1E5**	PI-PLC X domain-containing protein 2 isoform a OS=Heterocephalus glaber OX=10181 GN=PLCXD2 PE=4 SV=1
**G5C7R9**	Proteasome subunit beta OS=Heterocephalus glaber OX=10181 GN=PSMB1 PE=3 SV=1
**G5AYX6**	Pyridoxal phosphate phosphatase OS=Heterocephalus glaber OX=10181 GN=GW7_02557 PE=3 SV=1
**G5B4V6**	Major prion protein OS=Heterocephalus glaber OX=10181 GN=GW7_03223 PE=3 SV=1
**G5AMX5**	Wiskott-Aldrich syndrome protein family member 3 OS=Heterocephalus glaber OX=10181 GN=GW7_15950 PE=4 SV=1
**G5C835**	Propionyl-CoA carboxylase beta chain, mitochondrial OS=Heterocephalus glaber OX=10181 GN=GW7_21808 PE=4 SV=1
**G5BJB4**	Tubulin-specific chaperone D OS=Heterocephalus glaber OX=10181 GN=GW7_04129 PE=4 SV=1
**G5BA11**	Inositol 1,4,5-trisphosphate receptor type 1 OS=Heterocephalus glaber OX=10181 GN=GW7_04196 PE=4 SV=1
**G5AQ31**	Microsomal glutathione S-transferase 3 OS=Heterocephalus glaber OX=10181 GN=MGST3 PE=4 SV=1
**G5AU96**	Glycogen synthase kinase-3 beta OS=Heterocephalus glaber OX=10181 GN=GW7_00983 PE=3 SV=1
**G5AW75**	Non-specific serine/threonine protein kinase OS=Heterocephalus glaber OX=10181 GN=PAK1 PE=4 SV=1
**G5BFY1**	Inactive hydroxysteroid dehydrogenase-like protein 1 OS=Heterocephalus glaber OX=10181 GN=GW7_15943 PE=3 SV=1
**G5BPN1**	Solute carrier family 2, facilitated glucose transporter member 3 OS=Heterocephalus glaber OX=10181 GN=GW7_17311 PE=3 SV=1
**G5B8M0**	Proteasome subunit beta OS=Heterocephalus glaber OX=10181 GN=GW7_10314 PE=3 SV=1
**G5C8E9**	CtIP_N domain-containing protein OS=Heterocephalus glaber OX=10181 GN=GW7_09749 PE=4 SV=1
**G5AUL0**	Isocitrate dehydrogenase [NAD] subunit, mitochondrial OS=Heterocephalus glaber OX=10181 GN=GW7_10868 PE=3 SV=1

**G5BMR7**	Cleavage and polyadenylation specificity factor subunit 5 (Fragment) OS=Heterocephalus glaber OX=10181 GN=GW7_09347 PE=4 SV=1
**G5BPE1**	tRNA-splicing ligase RtcB homolog OS=Heterocephalus glaber OX=10181 GN=RTCB PE=3 SV=1
**G5CPG8**	Cytochrome c oxidase subunit 2 OS=Heterocephalus glaber OX=10181 GN=COX2 PE=3 SV=1
**G5B795**	60S ribosomal protein L31 OS=Heterocephalus glaber OX=10181 GN=GW7_13242 PE=4 SV=1
**G5BGH1**	Methylmalonyl-CoA mutase, mitochondrial OS=Heterocephalus glaber OX=10181 GN=MUT PE=4 SV=1
**G5AKT8**	Serine/threonine-protein kinase TAO3 OS=Heterocephalus glaber OX=10181 GN=GW7_10184 PE=4 SV=1
**G5AUW0**	Glutamate decarboxylase 2 OS=Heterocephalus glaber OX=10181 GN=GW7_03484 PE=3 SV=1
**G5C0H1**	E3 ubiquitin-protein ligase UBR5 (Fragment) OS=Heterocephalus glaber OX=10181 GN=GW7_07223 PE=4 SV=1
**G5B319**	Janus kinase and microtubule-interacting protein 1 OS=Heterocephalus glaber OX=10181 GN=GW7_14552 PE=4 SV=1
**G5ALH5**	ATP-dependent RNA helicase DDX3Y OS=Heterocephalus glaber OX=10181 GN=GW7_13691 PE=3 SV=1
**G5BW10**	Epidermal growth factor receptor kinase substrate 8-like protein 1 OS=Heterocephalus glaber OX=10181 GN=GW7_11661 PE=4 SV=1
**G5BBC0**	AP-3 complex subunit delta-1 OS=Heterocephalus glaber OX=10181 GN=GW7_07318 PE=4 SV=1
**G5BXB6**	Protein KIAA1045 OS=Heterocephalus glaber OX=10181 GN=GW7_03431 PE=4 SV=1
**G5AZ64**	Stress-70 protein, mitochondrial OS=Heterocephalus glaber OX=10181 GN=GW7_21299 PE=4 SV=1
**G5BF94**	Heat shock protein HSP 90-alpha 1 OS=Heterocephalus glaber OX=10181 GN=GW7_15044 PE=4 SV=1
**G5B7K7**	40S ribosomal protein S5 OS=Heterocephalus glaber OX=10181 GN=GW7_05598 PE=3 SV=1
**G5C8L3**	Cell adhesion molecule 2 (Fragment) OS=Heterocephalus glaber OX=10181 GN=GW7_20330 PE=4 SV=1
**G5B0Z2**	Interferon-induced protein with tetratricopeptide repeats 1-like protein OS=Heterocephalus glaber OX=10181 GN=GW7_17423 PE=4 SV=1
**G5AMI2**	U2 small nuclear ribonucleoprotein B OS=Heterocephalus glaber OX=10181 GN=SNRPB2 PE=4 SV=1
**G5AX47**	60S ribosomal protein L9 OS=Heterocephalus glaber OX=10181 GN=GW7_10035 PE=4 SV=1
**G5BH25**	Splicing factor 3B subunit 4 OS=Heterocephalus glaber OX=10181 GN=GW7_05788 PE=4 SV=1
**G5BPV1**	Vomeromodulin OS=Heterocephalus glaber OX=10181 GN=GW7_12307 PE=4 SV=1
**G5AVQ5**	PRA1 family protein OS=Heterocephalus glaber OX=10181 GN=ARL6IP5 PE=3 SV=1
**G5BBG5**	Cleavage and polyadenylation specificity factor subunit 6 OS=Heterocephalus glaber OX=10181 GN=GW7_05464 PE=4 SV=1
**G5BHP8**	Thy-1 membrane glycoprotein OS=Heterocephalus glaber OX=10181 GN=GW7_18692 PE=4 SV=1
**G5BVT2**	Mothers against decapentaplegic homolog OS=Heterocephalus glaber OX=10181 GN=GW7_16263 PE=3 SV=1
**G5BSL5**	Tetratricopeptide repeat protein 35 OS=Heterocephalus glaber OX=10181 GN=GW7_02334 PE=4 SV=1
**G5BP98**	Ankyrin repeat domain-containing protein 16 (Fragment) OS=Heterocephalus glaber OX=10181 GN=GW7_20020 PE=4 SV=1
**G5ARR7**	Nik-related protein kinase OS=Heterocephalus glaber OX=10181 GN=GW7_10189 PE=4 SV=1
**G5AN12**	Heat shock protein HSP 90-alpha OS=Heterocephalus glaber OX=10181 GN=GW7_15795 PE=4 SV=1
**G5BG92**	CAP-Gly domain-containing linker protein 2 OS=Heterocephalus glaber OX=10181 GN=GW7_08910 PE=4 SV=1
**G5BI54**	Armadillo repeat-containing protein 10 OS=Heterocephalus glaber OX=10181 GN=GW7_03899 PE=4 SV=1
**G5BVE2**	26S proteasome non-ATPase regulatory subunit 12 isoform 2 OS=Heterocephalus glaber OX=10181 GN=PSMD12 PE=4 SV=1
**G5AK02**	Selenium-binding protein 1 (Fragment) OS=Heterocephalus glaber OX=10181 GN=GW7_10561 PE=4 SV=1
**G5BAZ4**	40S ribosomal protein S18 (Fragment) OS=Heterocephalus glaber OX=10181 GN=GW7_20354 PE=3 SV=1
**G5BEK8**	Lipid phosphate phosphatase-related protein type 4 OS=Heterocephalus glaber OX=10181 GN=GW7_19429 PE=4 SV=1

**G5AM31**	Heterogeneous nuclear ribonucleoprotein U (Fragment) OS=Heterocephalus glaber OX=10181 GN=GW7_16292 PE=4 SV=1
**G5BBS9**	Annexin OS=Heterocephalus glaber OX=10181 GN=GW7_02904 PE=3 SV=1
**G5AQC9**	V-type proton ATPase proteolipid subunit OS=Heterocephalus glaber OX=10181 GN=ATP6V0C PE=3 SV=1
**G5C2G1**	Cathepsin D (Fragment) OS=Heterocephalus glaber OX=10181 GN=GW7_00398 PE=3 SV=1
**G5AJX1**	Metabotropic glutamate receptor 3 OS=Heterocephalus glaber OX=10181 GN=GW7_15125 PE=3 SV=1
**G5BJ98**	Coiled-coil domain-containing protein 57 OS=Heterocephalus glaber OX=10181 GN=GW7_04113 PE=4 SV=1
**G5C1Y9**	Exportin-1 OS=Heterocephalus glaber OX=10181 GN=GW7_01327 PE=4 SV=1
**G5ALC9**	Sentrin-specific protease 5 OS=Heterocephalus glaber OX=10181 GN=GW7_00073 PE=4 SV=1
**G5BIK5**	Synaptobrevin homolog YKT6 OS=Heterocephalus glaber OX=10181 GN=YKT6 PE=3 SV=1
**G5BK21**	Ras GTPase-activating protein SynGAP OS=Heterocephalus glaber OX=10181 GN=GW7_09861 PE=4 SV=1
**G5BGL1**	3-hydroxyisobutyryl-CoA hydrolase, mitochondrial (Fragment) OS=Heterocephalus glaber OX=10181 GN=GW7_01417 PE=3 SV=1
**G5B3Z0**	Xanthine dehydrogenase/oxidase OS=Heterocephalus glaber OX=10181 GN=GW7_02630 PE=4 SV=1
**G5BNM3**	Multidrug resistance-associated protein 7 OS=Heterocephalus glaber OX=10181 GN=GW7_19146 PE=4 SV=1
**G5BHK5**	FXYD domain-containing ion transport regulator OS=Heterocephalus glaber OX=10181 GN=GW7_18649 PE=3 SV=1
**G5C5Q8**	60S ribosomal protein L8 OS=Heterocephalus glaber OX=10181 GN=GW7_19216 PE=3 SV=1
**G5BTA7**	15 kDa protein A OS=Heterocephalus glaber OX=10181 GN=GW7_17264 PE=3 SV=1
**G5BB91**	40S ribosomal protein S26 OS=Heterocephalus glaber OX=10181 GN=GW7_15770 PE=3 SV=1
**G5AU42**	Voltage-dependent L-type calcium channel subunit beta-3 OS=Heterocephalus glaber OX=10181 GN=GW7_14474 PE=4 SV=1
**G5ASC8**	Endophilin-B2 OS=Heterocephalus glaber OX=10181 GN=GW7_01898 PE=4 SV=1
**G5C1R6**	Transcriptional repressor protein YY1 OS=Heterocephalus glaber OX=10181 GN=GW7_07411 PE=4 SV=1
**G5ATF3**	60S ribosomal protein L23a OS=Heterocephalus glaber OX=10181 GN=GW7_14614 PE=3 SV=1
**G5BPR5**	NADH dehydrogenase [ubiquinone] 1 alpha subcomplex subunit 6 OS=Heterocephalus glaber OX=10181 GN=GW7_19453 PE=3 SV=1
**G5AYI8**	Heterogeneous nuclear ribonucleoprotein U-like protein 2 OS=Heterocephalus glaber OX=10181 GN=GW7_01782 PE=4 SV=1
**G5BMY3**	Golgi SNAP receptor complex member 2 isoform A OS=Heterocephalus glaber OX=10181 GN=GOSR2 PE=3 SV=1
**G5BJM5**	60S ribosomal protein L12 OS=Heterocephalus glaber OX=10181 GN=GW7_00296 PE=3 SV=1
**G5AXN2**	60S ribosomal protein L29 OS=Heterocephalus glaber OX=10181 GN=GW7_07753 PE=4 SV=1
**G5AZL1**	40S ribosomal protein S15 OS=Heterocephalus glaber OX=10181 GN=RPS15 PE=3 SV=1
**G5B5R0**	Purine nucleoside phosphorylase (Fragment) OS=Heterocephalus glaber OX=10181 GN=GW7_14832 PE=4 SV=1
**G5BFU9**	N6-adenosine-methyltransferase 70 kDa subunit OS=Heterocephalus glaber OX=10181 GN=METTL3 PE=3 SV=1
**G5BFK4**	Lysine-specific demethylase 5A OS=Heterocephalus glaber OX=10181 GN=GW7_04041 PE=4 SV=1
**G5AUX0**	Optic atrophy 3 protein OS=Heterocephalus glaber OX=10181 GN=GW7_18095 PE=4 SV=1
**G5CA43**	COP9 signalosome complex subunit 2 OS=Heterocephalus glaber OX=10181 GN=GW7_07415 PE=4 SV=1
**G5B630**	Small nuclear ribonucleoprotein Sm D3 (Fragment) OS=Heterocephalus glaber OX=10181 GN=SNRPD3 PE=3 SV=1
**G5B6F6**	Stress-induced-phosphoprotein 1 OS=Heterocephalus glaber OX=10181 GN=STIP1 PE=4 SV=1
**G5ANG5**	Cytoplasmic dynein 1 intermediate chain 2 OS=Heterocephalus glaber OX=10181 GN=GW7_10798 PE=4 SV=1
**G5AZT3**	40S ribosomal protein S25 OS=Heterocephalus glaber OX=10181 GN=GW7_12498 PE=4 SV=1
**G5B843**	RNA-binding protein 39 OS=Heterocephalus glaber OX=10181 GN=RBM39 PE=4 SV=1
**G5AY62**	Glycine cleavage system P protein (Fragment) OS=Heterocephalus glaber OX=10181 GN=GW7_14031 PE=3 SV=1

**G5BXV7**	WD repeat-containing protein 24 OS=Heterocephalus glaber OX=10181 GN=WDR24 PE=4 SV=1
**G5BSY3**	Zinc finger C3H1 domain-containing protein OS=Heterocephalus glaber OX=10181 GN=GW7_18229 PE=4 SV=1
**G5BYX2**	Hook-like protein 2 OS=Heterocephalus glaber OX=10181 GN=GW7_16092 PE=4 SV=1
**G5C733**	NADH dehydrogenase (Ubiquinone) 1 alpha subcomplex subunit 4 OS=Heterocephalus glaber OX=10181 GN=NDUFA4 PE=4 SV=1
**G5BV08**	TATA-binding protein-associated factor 172 OS=Heterocephalus glaber OX=10181 GN=GW7_21610 PE=4 SV=1
**G5BA34**	Receptor expression-enhancing protein OS=Heterocephalus glaber OX=10181 GN=GW7_10077 PE=3 SV=1
**G5B224**	Hsp90 co-chaperone Cdc37 OS=Heterocephalus glaber OX=10181 GN=CDC37 PE=4 SV=1
**G5AUW3**	Abl interactor 1 OS=Heterocephalus glaber OX=10181 GN=GW7_03487 PE=4 SV=1
**G5ASG4**	60 kDa SS-A/Ro ribonucleoprotein isoform 2 OS=Heterocephalus glaber OX=10181 GN=TROVE2 PE=4 SV=1
**G5B398**	Neuron navigator 1 (Fragment) OS=Heterocephalus glaber OX=10181 GN=GW7_12877 PE=4 SV=1
**G5AQ82**	Structural maintenance of chromosomes protein OS=Heterocephalus glaber OX=10181 GN=GW7_15234 PE=3 SV=1
**G5BYM6**	Proline-rich transmembrane protein 2 (Fragment) OS=Heterocephalus glaber OX=10181 GN=GW7_11901 PE=4 SV=1
**G5C354**	Cytoplasmic aconitate hydratase OS=Heterocephalus glaber OX=10181 GN=GW7_13107 PE=2 SV=1
**G5B6T5**	Regulator of nonsense transcripts 2 OS=Heterocephalus glaber OX=10181 GN=GW7_21508 PE=4 SV=1
**G5C3G3**	Valyl-tRNA synthetase (Fragment) OS=Heterocephalus glaber OX=10181 GN=GW7_04793 PE=3 SV=1
**G5B1U5**	Propionyl-CoA carboxylase alpha chain, mitochondrial OS=Heterocephalus glaber OX=10181 GN=GW7_10944 PE=4 SV=1
**G5BJD7**	40S ribosomal protein S16 OS=Heterocephalus glaber OX=10181 GN=RPS16 PE=3 SV=1
**G5BZX2**	Kinase OS=Heterocephalus glaber OX=10181 GN=GW7_19927 PE=3 SV=1
**G5AL57**	Interphotoreceptor matrix proteoglycan 1 OS=Heterocephalus glaber OX=10181 GN=GW7_21555 PE=4 SV=1
**G5C9D3**	Guanine nucleotide-binding protein subunit beta-5 OS=Heterocephalus glaber OX=10181 GN=GW7_04217 PE=4 SV=1
**G5B580**	Single-stranded DNA-binding protein, mitochondrial (Fragment) OS=Heterocephalus glaber OX=10181 GN=GW7_11856 PE=3 SV=1
**G5BCV4**	Rab-interacting lysosomal protein OS=Heterocephalus glaber OX=10181 GN=GW7_05093 PE=4 SV=1
**G5BJM9**	Disks large-associated protein 3 OS=Heterocephalus glaber OX=10181 GN=GW7_00407 PE=4 SV=1
**G5B304**	SH3 domain and tetratricopeptide repeats-containing protein 1 OS=Heterocephalus glaber OX=10181 GN=GW7_14568 PE=4 SV=1
**G5C420**	Dynein light chain OS=Heterocephalus glaber OX=10181 GN=DYNLL2 PE=3 SV=1
**G5C6S8**	Coronin OS=Heterocephalus glaber OX=10181 GN=GW7_09953 PE=3 SV=1
