# Supplementary figures and images for: Acute Hypoxia Alters Extracellular Vesicle Signatures and the Brain Citrullinome of Naked Mole-Rats (Heterocephalus glaber)
Source: Int J Mol Sci. 2022 Apr 23;23(9):4683. doi: 10.3390/ijms23094683 (PMC9100269; doi:10.3390/ijms23094683)

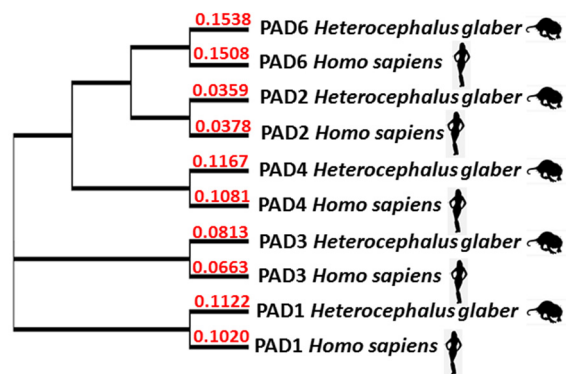

**Figure S1.** Phylogeny PAD (1).

Supplement: Supplementary file 1 [file ijms-23-04683-s001.zip › Supp Fig S1 Phylogeny PAD (1).pdf]
